# Transcriptional Regionalization of the Fruit Fly’s Airway Epithelium

**DOI:** 10.1371/journal.pone.0102534

**Published:** 2014-07-14

**Authors:** Muhammad N. Faisal, Julia Hoffmann, Samar El-Kholy, Kimberley Kallsen, Christina Wagner, Iris Bruchhaus, Christine Fink, Thomas Roeder

**Affiliations:** 1 University of Kiel, Dept. Molecular Physiology, Kiel, Germany; 2 Research Center Borstel, Priority Area Allergy and Asthma, Borstel, Germany; 3 Bernhard-Nocht Institute for Tropical Medicine, Dept. Molecular Parasitology, Hamburg, Germany; 4 German Center for Lung Research (DZL), Airway Research Center North (ARCN), Germany; Stockholm University, Sweden

## Abstract

Although airway epithelia are primarily devoted to gas exchange, they have to fulfil a number of different tasks including organ maintenance and the epithelial immune response to fight airborne pathogens. These different tasks are at least partially accomplished by specialized cell types in the epithelium. In addition, a proximal to distal gradient mirroring the transition from airflow conduction to real gas exchange, is also operative. We analysed the airway system of larval *Drosophila melanogaster* with respect to region-specific expression in the proximal to distal axis. The larval airway system is made of epithelial cells only. We found differential expression between major trunks of the airways and more distal ones comprising primary, secondary and terminal ones. A more detailed analysis was performed using DNA-microarray analysis to identify cohorts of genes that are either predominantly expressed in the dorsal trunks or in the primary/secondary/terminal branches of the airways. Among these differentially expressed genes are especially those involved in signal transduction. *Wnt*-signalling associated genes for example are predominantly found in secondary/terminal airways. In addition, some G-protein coupled receptors are differentially expressed between both regions of the airways, exemplified by those activated by octopamine or tyramine, the invertebrate counterparts of epinephrine and norepinephrine. Whereas the OAMB is predominantly found in terminal airway regions, the oct3βR has higher expression levels in dorsal trunks. In addition, we observed a significant association of both, genes predominantly expressed in dorsal trunks or in primary to terminal branches branches with those regulated by hypoxia. Taken together, this observed differential expression is indicative for a proximal to distal transcriptional regionalization presumably reflecting functional differences in these parts of the fly’s airway system.

## Introduction

Respiratory epithelia are central modules defining the structural and physiological properties of the lung. They represent an important interface with the outside world, contributing about 200 m^2^ of contact surface. Airway epithelial cells are far more than simple physical barriers, they react to different external and internal stimuli to maintain the overall organ structure, an optimal gas exchange and to orchestrate of the organ-specific immune response [1]–[3].

The mammalian airway epithelium is made of different cell types such as squamous ciliated, nonciliated bronchiolar, basal or neuroepithelial cells. In addition, a transcriptional and functional differentiation between proximal and distal airway epithelia has been observed [4], [5]. In contrast to mammals, airway epithelia of simple invertebrates are believed to be of much lesser complexity. Of special interest is the fruit fly *Drosophila melanogaster*, which has served as the most important invertebrate model since more than a century. The airway system of the fly, the tracheal system, is believed to contain epithelial cells only [6]–[8]. Although the tracheal system is not homologous to the mammalian lung, the architecture and physiology of both organs share a huge number of commonalities, presumably because airway epithelial cells are confronted with very special needs. These commonalities have been reported for diverse functional modules such as the epithelial innate immune system or the liquid clearance [7], [9]. It turned out that these similarities are unexpectedly far-reaching, which enabled the development of *Drosophila* models for inflammatory diseases of the lung, such as asthma [10]–[12]. Although much simpler organized and seemingly with a more homogenous structure throughout the entire organ, special cell types have been identified in the *Drosophila* airways. During development, the embryonic airway system is made out of at least four different cell types, giving rise to the different structural elements of the tracheal system [13], [14]. The larval tracheal system is a hierarchically organized system of interconnected tubes with terminal blind endings [6]. Major (dorsal) trunks are connected to the outside via stigmata and primary branches originating from these trunks ones give rise to secondary and terminal branches, which usually terminate in a huge number of blind endings [8], [13]. Whereas epithelial cells in the dorsal trunks are only part of a single layered cell sheet surrounding the central airway, terminal cells usually make various terminal endings in finger-shaped structures [6]. Thus, a transcriptional gradient from proximal to distal that matches the functional differences can be anticipated [13]. Moreover, structurally and functionally different cells are present in the larval tracheal system including hormone producing peritracheal or Inka cells [15], [16] as well as stem cells that repopulate the adult tracheal system. These stem cells have some very unique features and should obviously carry very specific transcriptional signatures [17], [18].

To evaluate if a presumed regional functionalization of the fruit fly’s airways has its equivalent at the transcript level, differential transcription between the dorsal trunks that presumably function in conducting airflow and the terminal structures (primary to terminal branches) that are devoted to gas exchange was addressed. Using promoter::Gal4 lines, we initially found differences in the expression patterns of a number of different genes that tempted us to study this more systematically. Using DNA-microarray analysis of manually isolated dorsal trunks and primary/secondary/terminal ones, we detected sets of genes that are more abundant in either the primary or the secondary/tertiary ones, resembling the differences between proximal and distal airways in this important model system.

## Materials and Methods

### Fly strains and husbandry

Stocks were raised on standard cornmeal-agar medium at 25°C under a 12 h/12 h light dark cycle. The w^1118^ strain was used as the wild-type. The transgenic strains ppk11-Gal4, amon-Gal4, OAMB-Gal4, OA2-Gal4, oct2βR-Gal4, UAS-gfp have been described elsewhere or will be described below [9], [10].

This study was carried out in strict accordance with the recommendations in the Guide for the Care and Use of Laboratory Animals of the german local authorities.

### Molecular biology

Early third-instar larvae were used for all experiments. Following manual isolation of the trachea in ice-cold PBS, the tissue was further divided into dorsal trunks devoid of any other parts of the tracheal system and the remaining parts of the tracheal system comprising primary, secondary and terminal branches. High purity of these tracheal preparations (see Fig. S1) was of prime importance for all downstream experiments. Special attendance was on removing all adhering remnants of other organ (especially fat body) from the tracheal preparations. Only those tracheal parts that were unequivocally devoid of any contaminations were further processed. Thus, only if molecular markers of fat body or hemocytes, as the two most important sources of contamination could not be not found, samples were used for downstream analyses (see below). The purified tracheae were then transferred to denaturation solution (NucleoSpin RNA II, Macherey-Nagel, Dueren, Germany) and homogenized. RNA was used for conventional cDNA synthesis or to produce capfinder cDNA, as described previously [19], [20], for use in qRT-PCR experiments or microarray analysis, respectively. cDNA synthesized from tracheal material was used for downstream applications only if RT-PCR with primers for *hemese* (hemocytes) and *P6* (fat body) revealed no contamination with either of these materials. Quantitative RT-PCR was performed with an Applied Biosystems StepOne Cycler (Applied Biosystems, Darmstadt, Germany) using the DyNAmo Flash SYBR Green Kit (Fisher Scientific, Schwerte, Germany). Probe sets were normalized against the housekeeping gene *rpl 13*. At least three independent biological replicates were analyzed. All statistical analyses were performed with the GraphPad Prism 6 program package.

To generate the promoter-Gal4 lines, the presumptive promoter regions of the genes of interest (usually approximately 1 kb upstream of the transcriptional start site) were amplified by PCR and genomic DNA served as template. After purification the amplicon was cloned into the pPTGal4 vector [21]. The corresponding vectors were used for transformation of w^1118^ flies; injection of oocytes was performed by the BestGene Corp. (China Hills, CA, USA) The corresponding flies were crossed to a UAS-gfp line to visualize expression sites.

### DNA microarray, GO ontology and statistical analysis

Microarray analyses were essentially performed as described earlier [7], [19]. In brief, cDNA synthesis from RNA isolated from primary tracheal branches or secondary and terminal branches was performed with Prime Script RT (Takara Bio Europe, Saint-Germain-en-Laye, France) using the following primers: OdT T7 I (5′-GAG AGA GGA TCC AAG TAC TAA TAC GAC TCA CTA TAG GGA GAT TTT TTT TTT TTT TTT TTT TTT T G/A/C-3′) and CapFinder Sp6rG (5′-CAG CGG CCG CAG ATT TAG GTG ACA CTA TAG A rGrGrG-3′). cDNA was amplified with OdT T7 II (5′-GAG AGA GGA TCC AAG TAC TAA TAC GAC TCA CTA TAG G-3′) and Adaptor Sp6rG (5′-GAC GCC TGC AGG CGA TGA ATT TAG G-3′) and LA Taq polymerase (25–27 cycles; 95°C 20 s, 53°C 20 s, 72°C 2 min 30 s, 5 min final extension). cDNA was transcribed with MEGAscript T7 (including aminoallyl-UTP, 1∶1 mixed with UTP) for 4 h at 37°C. Following purification, the transcribed RNA was labeled with AlexaFlour reactive dyes (Life technologies, Darmstadt, Germany, solved in DMSO) at a pH of 9.0 for 2 h. After labeling, samples were purified and used for hybridization in DIG easy Hyb (Roche Applied Sciences, Mannheim, Germany) supplemented with salmon testes DNA and yeast tRNA at 42°C overnight. Stringency washes were performed in 0.1XSSC at 37°C. Samples were hybridized to *Drosophila* 14 k V2 (Canadian *Drosophila* Microarray Centre, Toronto, Canada) scanned using a GenePix 4000 B Microarray Scanner (Axon Instruments, Science Products, Hofheim, Germany) at a resolution of 10 µm. Data acquisition, normalization and analysis including hierarchical clustering were carried out with GenePix 6.0 and Acuity 4.1 (Molecular Devices, Biberach, Germany). Gene Ontology analysis was performed with the DAVID program package [22].

The primary data have been deposited at the GEO database with the accession number GSE53273.

### Oligonucleotides

All oligonucleotides used for this study ale listed in Table S5.

## Results

**Figure 1:**
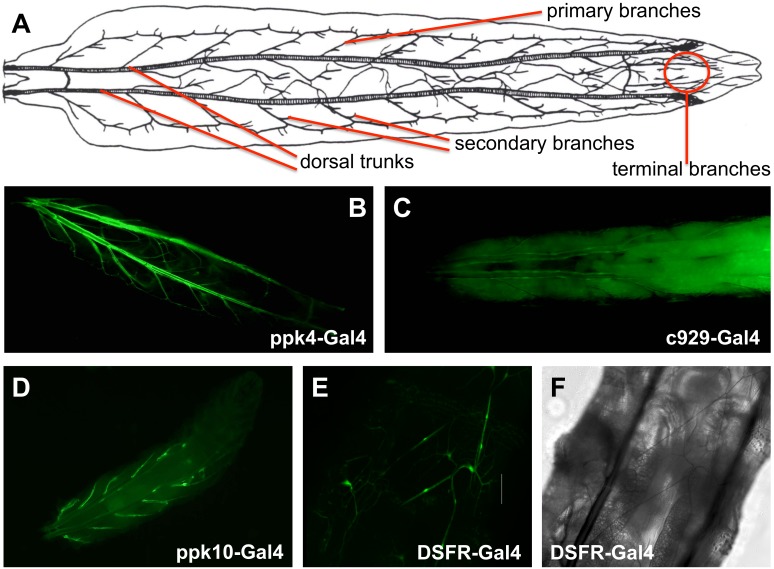
The airway epithelium of *Drosophila* larvae is characterized by different expression domains. The larval airway system is composed of interconnected tubes organized in a hierarchic structure (adapted after [6], A). Dorsal trunks are connected to the outside world and primary branches originate from them. The blind endings of the tracheal system are made from terminal branches (A). Different promoter Gal4 lines direct expression into different parts of the tracheal system. Driver lines such as *ppk4*-Gal4 label the entire airway system (B). In contrast, *c929*-Gal4 is specific for structures in the dorsal trunks (C), *ppk10*-Gal4 is surprisingly specific for primary branches (D) and *DSRF*-Gal4 labels exclusively terminal cells (E fluorescent channel, F transmitted light). Scale bars in B, C, D is 50 µm, in E, F 20 µm.

Pan-tracheal driver lines such as btl-Gal4 or ppk4-Gal4 have been used to visualize and/or manipulate the airway epithelium throughout the entire tracheal system (Fig. 1A, B). The analysis of various promotor-Gal4 lines that can be used to manipulate airway epithelial cells showed region specific expression patterns in the larval tracheal system of the fruit fly *Drosophila melanogaster*. Amon-Gal4 (*amontillado*- or c929-Gal4, [23]) confines ectopic expression to very few cells associated with the dorsal trunk (Fig. 1C). The corresponding protein is required to process peptide hormones and is thus present in peptide producing cells, primarily located in the nervous system, but also in a specific subgroup of cells of the tracheal system, namely the so called Inka cells, known to produce the ecdysis triggering hormone (ETH) that are located at the branching points to the primary branches of the airway system. In contrast, ppk10-Gal4 (coding for a sodium channel of the pickpocket family, [9]) drives expression exclusively into the primary branches of the airway system, almost completely omitting dorsal trunks and secondary and terminal branches (Fig. 1D). The third major compartment of the tracheal system, the terminal branches, are specifically targeted by a number of different Gal4 lines including DSRF-Gal4 [24]. This is demonstrated in Fig. 1E and F, where the fluorescent channel allows visualization of the labelled terminal cells and the matching transmitted light picture shows that neither dorsal trunks, nor primary or secondary branches are labelled.

**Figure 2:**
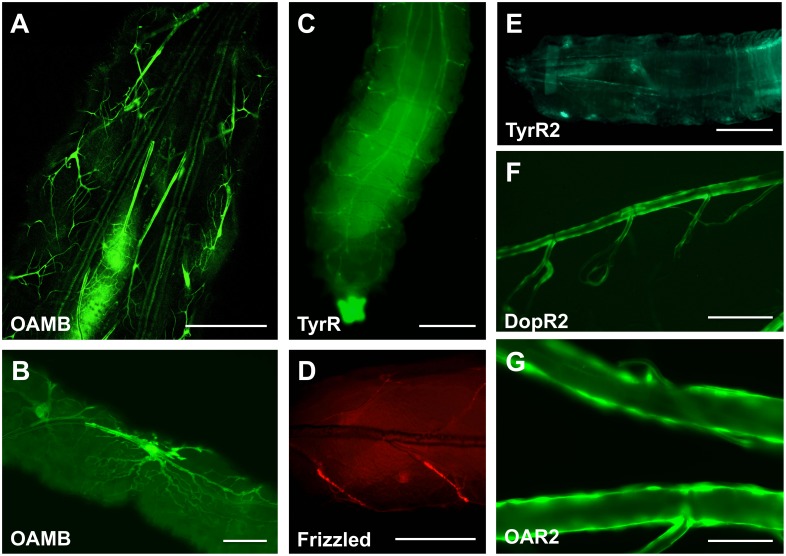
Expression pattern of selected G-protein coupled receptors in the airway system of *Drosophila* larvae. Promotor-Gal4 lines of different G-protein coupled receptors were used to study their expression pattern in the airway system. The promoter of the OAMB receptor drives expression to terminal branches or cells (A overview, B intestine with terminal cell). The *TyrR* show higher expression in primary branches (C), the *frizzled* receptor is also predominantly found in these structures (D) octopamine/tyramine receptors drive expression in different parts of the airway system. The Oct2βR-Gal4 is primarily present in the dorsal trunks (A). The OAMB receptor can be observed in the primary airways (B). Especially the terminal cells located directly adjacent to different organ systems show a strong expression of this receptor (C). The OA2 receptor is also found predominantly in the primary airways (D). In contrast, the TyrRII (E), the DopR2 (F) and the OAR2 (G) show a broader expression pattern. Scale bars in A, C, D, E is 50 µm, in B, F 20 µm and in G 10 µm.

To further evaluate region-specific transcription in these different parts of the tracheal system that might be related to adaptive processes in the airway epithelium to varying physiological conditions, we analysed the expression patterns of genes involved in G-protein signalling in peripheral tissues. One special focus was on receptors for octopamine and tyramine, the invertebrate counterparts of epinephrine and norepinephrine [25], [26], because it has been shown that different alpha- and β-adrenergic receptors control various facets of airway epithelial physiology in man [27], [28]. Thus, we looked at the expression pattern of these receptors using the corresponding promoter-Gal4 lines. These receptors, comprising the OAMB-, the OAR2-, the TyrR- and TyrRII-, but also the DopR2- and the Frizzled receptors (G-protein coupled receptor of the *Wnt*-signaling pathway) were analysed in more detail regarding their differential expression in the proximal to distal axis of the airways. The OAMB receptor shows almost no expression in the major trunks of the airways or in the secondary branches but is highly transcribed in the terminal cells (Fig. 2A), including those that contact internal organs such as the intestine (Fig. 2B). The *TyrR* receptor is also predominantly expressed in the secondary branches of the airway epithelium but in a different pattern as the *OAMB* receptor (Fig. 2C). Other members of the octopamine/tyramine receptor family show a less region specific expression. Both, the *TyrRII*- and the *OAR2* receptors are expressed in both dorsal trunks as well as the primary and secondary branches, with a bias for the dorsal trunks (Fig. 2E, G). This is similar as found for another biogenic amine receptor, the *DoprR2*-receptor (Fig. 2F). On the other hand, the frizzled receptor, which serves as the G-protein coupled receptor for *Wnt* ligands, is specifically found in structures associated with primary and secondary branches, most prominent in regions with stem cell nests (Fig. 2D).

Based on these mosaic observations, we started an analysis aiding to identify the complete sets of genes with enriched expression in the dorsal trunks versus the primary, secondary and terminal ones and *vice versa*. For this, trachea of early 3^rd^ instar larvae were prepared manually, freed from attached cellular material as already described [7], [10] and divided into the dorsal trunks and the remainder of the tracheal system, containing the primary, secondary and terminal branches (Fig. S1). Special attendance was on pure preparations that were clearly devoid of any adhering material such as fat body remnants. These samples were used for DNA-microarray analyses enabling us to evaluate the relative transcription levels in these samples. The primary data have been deposited at the GEO database with the accession number GSE53273.

**Figure 3:**
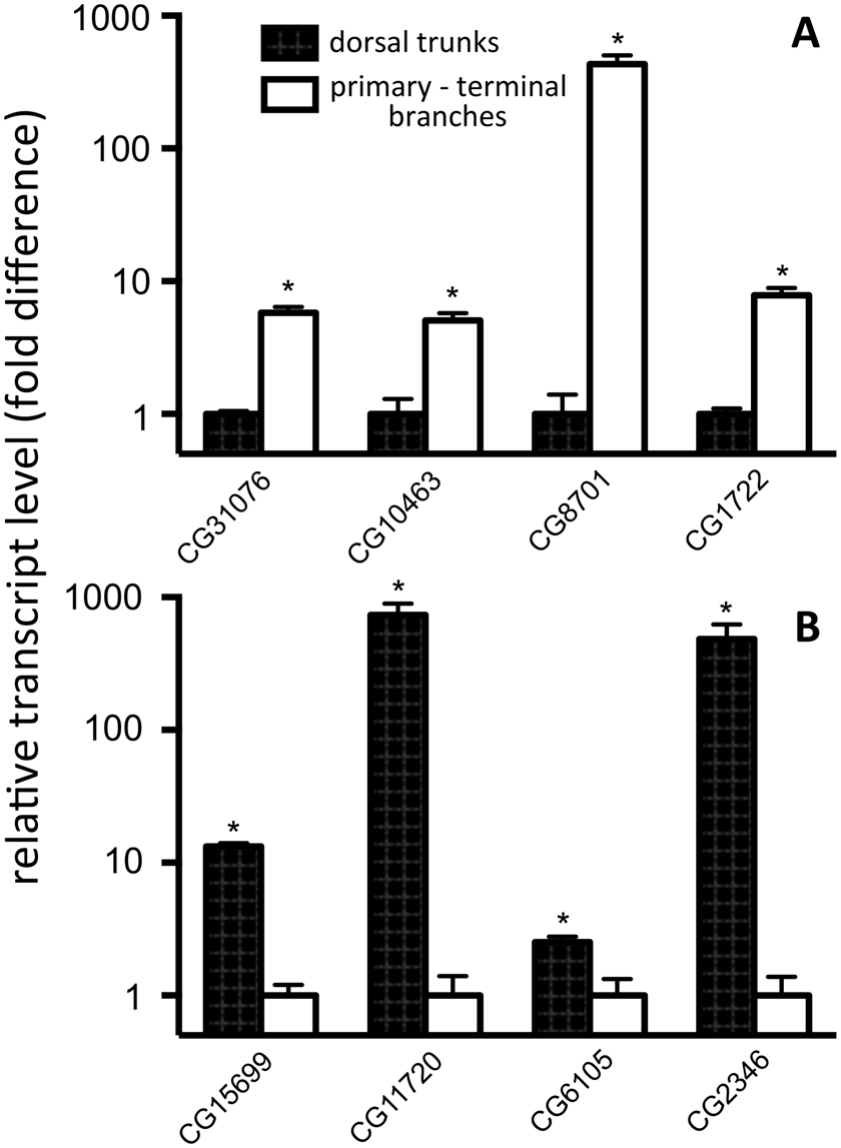
qRT-PCR analyses of randomly selected genes with predicted differential expression between dorsal trunks and primary/secondary/terminal branches. Differential expression of the listed genes was evaluated by qRT-PCR with cDNA derived from dorsal trunks or primary, secondary and terminal airways. Predominant expression was predicted from DNA-microarray studies for 4 transcripts in the dorsal trunks (A) or in the primary, secondary and terminal ones (B). Values are mean results from three different experiments performed in triplicate (±S.D.). Asterisks show statistically significant differences.

Based on these studies, we identified 307 genes with an at least 1.5 fold higher relative transcription in primary branches compared with primary/secondary/terminal branches and 336 genes with an at least 1.5 fold higher transcription in primary/secondary/terminal ones compared with dorsal trunks (Table S1, S2). To verify results obtained from the DNA microarray studies, we randomly selected eight candidate genes enriched either in the dorsal trunks or the primary, secondary and terminal branches respectively. Using qRT-PCR analysis focussed on the corresponding transcripts and cDNA derived from either the primary or the secondary and terminal branches, the relative transcript levels in both parts of the airways were quantified using at least 3 independent biological samples. As predicted from the DNA-micorarray studies, the differential transcription patterns could be verified for all eight genes. Four of them (CG2346, CG6105, CG11720, CG15699) show a significantly higher transcription in dorsal trunks, whereas the other four (CG1722, CG8701, CG10463, CG31076) are transcribed at higher levels in the primary, secondary and terminal branches (p<0.05; Fig. 3). Especially those predominantly present in the dorsal trunks are of interest, as they code for proteins with known function. CG2346 is also known as *FMRFamide*, a peptide hormone found in most invertebrates. CG11720 is on the other hand known as *sgs3*, the salivary gland specific protein 3, which is a major constituent of the saliva, and CG15699 is known as *Strn/Mlck*, which is a myosin light chain kinase relevant for Ca^2+^-mediated signalling. As sgs3 expression is known to be highly specific for the salivary gland [29], we can exclude that our tracheal branch preparations (containing the primary, secondary and terminal tracheal branches) were contaminated by fragments of salivary glands or other tissues. Nevertheless, it has to be mentioned that sgs3 is also expressed in trachea although the level of expression is less than 1% compared to the salivary glands [30].

**Figure 4:**
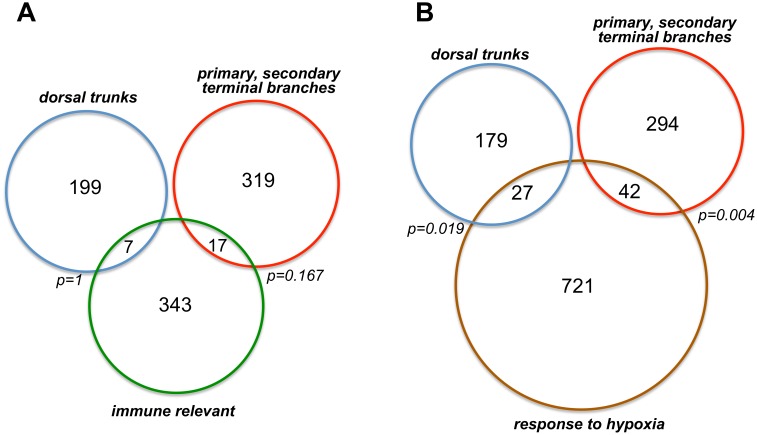
Venn diagram analysis of genes predominantly expressed in dorsal trunks or primary, secondary and terminal branches with those involved in defence responses (A) or regulated by hypoxia (B). The sets of genes predominantly expressed in dorsal trunks or primary, secondary and terminal branches were compared with those linked to immunity (A) or responses to hypoxia (B). The numbers of genes found in both sets is listed in the intersecting regions. Statistical analyses were performed with Fisher’s exact test, the corresponding p-values are listed close to the intersections.

A closer look at the results revealed strong differences between both groups of genes. Bioinformatic analyses using the DAVID program package [22] revealed that few gene ontologies are overrepresented in either the primary or the secondary and terminal branches. Among the ones overrepresented in the dorsal trunks are e.g. the GO terms biological adhesion, asymmetric protein localization, ligase activity, alternative splicing or programmed cell death. Enriched in the secondary and terminal ones are on the other hand: ribloflavin metabolism, oxidoreductase activity or vesicle mediated transport. KEGG pathway analysis revealed that the Glutathione metabolism is overrepresented in primary branches, whereas TGF-beta signalling is overrepresented in secondary and terminal ones. Comparing the sets of regulated genes with those obtained in other experimental setups revealed that the differential expression between primary and secondary as well as terminal airways has no connection to defence related responses. The set of canonical immune genes was obtained from the webpage of Bruno Lemaitre (http://lemaitrelab.epfl.ch/page-7767-en.html) and serves as the best curated source for this purpose. The comparison was performed with simple Venn-diagram analyses (Fig. 4A). Overlaps between the set of immune genes and those primarily present in dorsal trunks or primary, secondary and terminal branches were only marginal. Statistical analysis using Fisher’s exact test revealed that samples correlations are not statistically significant (p values of 1 primary branch specific *vs* immune genes and 0.166 for secondary/terminal branch specific *vs* immune genes). In addition, we compared the sets of genes with those obtained from a transcriptome study where *Drosophila* larval airways were subjected to hypoxia (Kallsen et al. in preparation). Airways of the w^1118^ strain were subjected to hypoxia (1% oxygen) for 24 h and manually isolated immediately after this exposure period. Those genes showing a regulation of more than 1.5 fold or less than 0.67 fold in two out of three biological replicates were included into this list comprising a total of 790 genes. 36 dorsal trunk specific genes and 42 primary, secondary and terminal branch specific ones were also hypoxia regulated (Fig. 4B). The overlaps were statistically significant (dorsal trunks *vs* hypoxia: p = 0.019, primary, secondary and terminal *vs* hypoxia p = 0.004). Among these genes found in dorsal trunks as well as being regulated following hypoxia are e.g stress associated genes such as Metallothionein A (*MtnA*) or heat shock protein cognate 1 (*Hsc-1*), but also those involved in signalling including the octopamine receptor β3 (*octRβ3*) (Table S3). Genes showing a preference for primary, secondary and terminal branches while being regulated by hypoxia comprise those involved in G-protein signalling such as G-protein beta 5 (*Gbeta5*), the adenylate cyclase 3 (*Ac3*) but also stress associated ones such as the superoxide dismutase 2 (*SOD2*) or *Rac2* (Table S4).

A selection of genes belonging to different functional categories is listed in table 1. As already pointed out, a surprisingly small number of these differentially expressed genes can be associated with defence mechanisms. In contrast, both regions of the tracheal system have predominant signatures of genes associated with tracheal development (e.g. *Rac2, armadillo and crooked*, in primary, secondary and terminal branches and *vrille* and *zyxin* in primary ones). As a special interest of our project were on signalling systems that may differ between both areas of the airway epithelium, we focused on G-protein coupled receptor signalling associated genes, as they play a central role for transducing information into the target cells. In primary branches, we have the *Trissin* receptor, the *Octbeta3* receptor, in the secondary and terminal branches, the *FMRFamide* receptor, the *TyrR* receptor, and the *Pyrokinin* receptor. Especially the *TyrR* receptor was of interest, because it was one of the candidates that we analysed in before using promoter-Gal4 lines (Fig. 2C). In these experiments, a predominant expression in the secondary branches became apparent, which was in good accordance with the microarray experiments. Moreover, dorsal trunks express a greater number and diversity of gustatory receptors in comparison to the primary, secondary and terminal ones. Most striking was the regionalization of *Wnt*-signaling. Whereas in dorsal trunks only one *Wnt*-associated (*naked cuticle*) gene was expressed at higher levels, secondary and terminal branches express *twins*, *microtubule stars* and *multiple wing hairs* at higher levels. More important is the higher expression of *armadillo*, the central component of the *Wnt*-signalling pathway. In addition, our promoter line studies revealed that the receptor of *Wnt*-signalling, frizzled is also present in higher amounts in secondary/terminal cells. In addition, we observed stress related genes primarily in this part of the tracheal system including *Turandot A* and *B*. Related to signalling was the observation that proteins that signatures associated with the *JNK*-pathway (*Mkk4, kayak, Rac2*) are overrepresented in the primary, secondary and terminal branches, similar to central molecules of the *Wnt*-signalling pathway, such as the β-catenin homolog *Armadillo*.

**Table 1:** Genes with increased transcription in either the dorsal trunks or the primary/secondary/terminal branches that can be assigned to selected functional categories.

dorsal trunk		primary/secondary/terminal branches	
***Defense***			
*virus-induced RNA1*	Vir-1	*immune induced* *molecule 1*	IM1
*metchnikowin*	mtk	*nimrodB2*	nimB2
*drosmycin-5*	Dro5	*drosomycin-4*	Dro4
*TNF-receptor-associated* *factor 4*	Traf4	*serpent*	srp
*melanization protease 1*	MP1	*Rac2*	Rac2
*lysozyme S*	lysS	*kayak*	kay
	Ntf2	*CG6639*	
*immune response deficient 1*	Ird1	*drosha*	drosha
*Lectin 46cb*			
*Peptidoglycan recognition protein-LD*	PGRP-LD		
			
***tracheal development***			
*Btk family kinase at 29A*	Btk29A	*Rac2*	Rac2
	Cad96C	*mummy*	mmy
*Vrille*	vri	*armadillo*	arm
*Zyxin*	zyx	*formin3*	From3
		*crooked*	crok
			
***GPCR signalling***			
*G protein gamma 1*	Gγ1	*FMRFreceptor*	FR
*trissin receptor*	TrissinR	*pyrokinin receptor*	Pk1R
*Octbeta3R*	Octbeta3R	*tyramine receptor*	TyrR
	CG32447	*G protein beta 5*	Gbeta5
	CG7918	*adenylate cyclase 3*	Ac3
*gustatory receptor 66a*	Gr66a	*hormone receptor* *like in 39*	Hr39
*gustatory receptor 93b*	Gr93b	*gustatory receptor 59e*	Gr59e
*gustatory receptor 97a*	Gr97a		
			
***Wnt-signaling***			
*naked cuticle*	nkd	*armadillo*	arm
		*twins*	tws
		*microtubule star*	mts
		*multiple wing hairs*	mwh
			
***JAK-STAT-/JNK-signalling***			
*CyclinE*	CycE	*kayak*	kay
		*map kinase kinase 4*	Mkk4
			
***Stress response***			
*Cyp313A2*		*Cyp313A1*	
*Cyp6a19*		*Cyp313A4*	
*Cyp9f2*		*thread*	th
*Cyp49A1*		*Hsp70(Hsp90* *organizing protein*	Hop
*heatshock protein 67Bb*	Hsp67Bb	*heatshock protein* *27*	Hsp27
*ferredoxin*	Fdxh	*superoxide dismutase 2*	SOD2
*multidrug resistance like protein*	Mrp	*turandot A*	TotA
*ubiquitin carboxyterminal hydrolase*	Uch	*turandotB*	TotB
*heatshock protein cognate 1*	Hsc70-1	*heatschock protein* *cognate-2*	Hsc70-2
*metallothioneinA*	MtnA		

## Discussion

In the current study, we were able to show a transcriptional regionalization between the primary and the secondary as well as terminal branches in the airway epithelium of larvae of the fruit fly *Drosophila melanogaster*. This type of regionalization has previously been shown for the mammalian lung, were the different parts of this complex organ fulfil different tasks and have different structures, which is reflected by regionalized transcription profiles corresponding to functional differences. Whereas air transportation is dominating in the proximal parts, gas exchange becomes more relevant the more distal the structure is. In the fly, a similar type of proximal to distal regionalization may be operative, showing a similar division of the two major tasks, air conduction and gas exchange. Regarding the accompanying transcriptional differences, especially those that enable physiological adaptations to different functional needs may be of special interest. In addition, the results presented here imply that the architectural similarities between the mammalian lung and the insect airway system go beyond the hierarchical organization with blind ending terminal structures primarily devoted to efficient gas-exchange.

Among the genes with differential expression between primary and secondary/tertiary airway branches various are associated with G-protein coupled receptor (GPCR) signalling. GPCR signalling is obviously the most relevant way to modulate the physiological state of a cell. One of our special foci was on octopamine/tyramine receptor signalling that is of prime importance to adapt central and peripheral tissues to different physiological needs [25], [31]. We found the Octbeta3R as well as the OA2 receptor to be higher expressed in dorsal trunks whereas the OAMB and the TyrR are expressed at higher levels in the primary/secondary/terminal branches. As these receptors are known to show different second-messenger coupling [32], the effects of both important stress hormones [33] should differ substantially between both parts of the airway system. In addition, the *Trissin* receptor is found at higher levels in the dorsal trunks, whereas the *pyrokinin* receptor in primary, secondary and terminal ones. Of special interest is the differential expression of *FMRFamide* and its receptor (*FMRFamide* receptor [34]). Whereas the peptide hormone is found primarily in the large dorsal trunks, its cognate receptor is restricted to the more terminal structures, a distribution that may be related to signalling between dorsal trunks and primary, secondary as well as terminal airways. This type of architecture, where ligand and receptor are segregated has been observed in other systems, e.g. in the EGFR signalling in the human airways, where it enables activation of repair mechanisms only after physical injury of the epithelium is experienced [35]. In addition, the dorsal trunks express a greater number and diversity of olfactory and gustatory receptors in comparison to the secondary and terminal ones, perhaps reflecting a greater probability of odorants being present in dorsal trunks rather than in the other parts of the airway system. We obtained the information about differential expression with both approaches (using of promoter-Gal4 lines and DNA-microarray studies) only for the *TyrR*. GPCRs are usually expressed at relatively low levels and are therefore not the prime candidates to be identified in DNA-microarray studies, which might be the reason for this discrepancy. This is also reflected in data obtained from the *Drosophila* Fly Atlas [30]. Data for expression in larval trachea (not distinguished between dorsal trunks and primary, secondary or terminal branches) were we obtained for none of the octopamine/tyramine receptors tested in promoter-Gal4 studies an unequivocal signal indicative for detectable expression levels in the trachea. This would imply that promoter-Gal4 lines are better suited especially for those genes showing only moderate or low expression levels. Nevertheless, it has to be kept in mind that these lines not necessarily show the complete expression profile of the gene of interest as important parts of the promoter may be missing. Alternative approaches suited for region-specific analysis of expression patterns such as *in situ* hybridization failed for the receptor genes of interest although we have wide experience with this method [36]. Airway epithelial cells are very large and flat, thus giving only low signal strengths, while they show a strong background signal.

In addition to the signalling systems mentioned above, others also differ between these parts of the airways. Proteins that can be attributed to the JNK-pathway (*Mkk4, kayak, Rac2*) are overrepresented in the secondary and terminal branches, similar to the central molecule of the *Wnt*-signalling pathway, the β-catenin Armadillo. Both signalling systems may be associated with the higher structural plasticity that is characteristic for the terminal airways [37]. *Wnt*-signalling has recently been associated with fate decisions in the developing mammalian lung [38]. Additional differences are obvious in the stress response genes specifically expressed in either of both areas indicating differential susceptibility to stressors. In the dorsal trunks, 4 different cytochrome genes are present at higher levels. In the primary, secondary and terminal branches, stress related genes such as TotA and TotB [39], [40], but also the superoxide dismutase 2 as a major ROS scavenger, are found at higher levels.

Dorsal trunks, primary, secondary as well as terminal airways share numerous similarities including their general architecture as single layered epithelia, but are on the other hand different regarding structural, functional and molecular aspects. Some of the transcriptional differences that were described in this project may be accountable to the functional differentiation from proximal to distal parts of the airways. In addition, the presence of very special cell types within the airway epithelium may contribute at least partially to these differences. In the region of the primary branches, very peculiar cell types, such as the Inka- or the fusion cells are present that have very peculiar functions and presumably also peculiar protein compositions enabling these special tasks. On the other hand, stem cells are found in the airway preparation containing secondary and terminal branches, where they reside until they rebuild the airway system during metamorphosis. In addition, the terminal cells of the airway epithelium show very peculiar characteristics, including an extraordinary capacity to react with the building of new branch structures to local hypoxia. The different physiological tasks, air conduction on one hand and gas exchange on the other hand, or differences in the way how to react to infections or stressors may be responsible for the observed differences at the transcript level between the dorsal trunks that might predominantly be devoted to gas transport and the primary, secondary as well as terminal branches that are responsible for gas exchange.

## Supporting Information

Figure S1 **Shown are typical examples of dorsal trunks (A), and primary, secondary, terminal branches (B) manually isolated from 3rd instar larvae that were used for downstream experiments.**
(PPTX)

Table S1 **Genes with 1.5 fold higher expression in dorsal trunks.**
(DOCX)

Table S2 **Genes with 1.5 fold higher expression in primary/secondary/terminal branches.**
(DOCX)

Table S3 **Genes predominantly expressed in dorsal trunks AND regulated by hypoxia.**
(DOCX)

Table S4 **Genes predominantly expressed in primary/secondary/terminal branches AND regulated by hypoxia.**
(DOCX)

Table S5 **Oligonucleotides used in this study.**
(DOCX)

## References

[1] Cheng DS, Han W, Chen SM, Sherrill TP, Chont M, et al (2007) Airway epithelium controls lung inflammation and injury through the NF-kappa B pathway. J Immunol 178: 6504–6513. 17475880 10.4049/jimmunol.178.10.6504

[2] Holgate ST (2007) The epithelium takes centre stage in asthma and atopic dermatitis. Trends Immunol 28: 248–251. 17466594 10.1016/j.it.2007.04.007

[3] Shah AS, Ben-Shahar Y, Moninger TO, Kline JN, Welsh MJ (2009) Motile cilia of human airway epithelia are chemosensory. Science 325: 1131–1134. 19628819 10.1126/science.1173869 PMC2894709

[4] Cardoso WV (2000) Lung morphogenesis revisited: old facts, current ideas. Developmental dynamics: an official publication of the American Association of Anatomists 219: 121–130. 11002333 10.1002/1097-0177(2000)9999:9999<::aid-dvdy1053>3.3.co;2-8

[5] Zhou J, Schmid T, Schnitzer S, Brune B (2006) Tumor hypoxia and cancer progression. Cancer Lett 237: 10–21. 16002209 10.1016/j.canlet.2005.05.028

[6] Ruehle H (1932) Das larvale Tracheensystem von Drosophila melanogaster Meigen und seine Variabilität. Zeitschrift für wissenschaftliche Zoologie 141: 159–245.

[7] Wagner C, Isermann K, Fehrenbach H, Roeder T (2008) Molecular architecture of the fruit fly’s airway epithelial immune system. BMC Genomics 9: 446. 18823557 10.1186/1471-2164-9-446 PMC2566315

[8] Whitten J (1957) The post-embryonic development of the tracheal system in Drosophila melanogaster. Q J Microsc Sci 98.

[9] Liu L, Johnson WA, Welsh MJ (2003) Drosophila DEG/ENaC pickpocket genes are expressed in the tracheal system, where they may be involved in liquid clearance. Proc Natl Acad Sci U S A 100: 2128–2133. 12571352 10.1073/pnas.252785099 PMC149970

[10] Wagner C, Isermann K, Roeder T (2009) Infection induces a survival program and local remodeling in the airway epithelium of the fly. Faseb J 23: 2045–2054. 19237508 10.1096/fj.08-114223

[11] Roeder T, Isermann K, Kabesch M (2009) Drosophila in asthma research. Am J Respir Crit Care Med 179: 979–983. 19342413 10.1164/rccm.200811-1777PP

[12] Roeder T, Isermann K, Kallsen K, Uliczka K, Wagner C (2012) A Drosophila asthma model - what the fly tells us about inflammatory diseases of the lung. Advances in experimental medicine and biology 710: 37–47. 22127884 10.1007/978-1-4419-5638-5_5

[13] Uv A, Cantera R, Samakovlis C (2003) Drosophila tracheal morphogenesis: intricate cellular solutions to basic plumbing problems. Trends in cell biology 13: 301–309. 12791296 10.1016/s0962-8924(03)00083-7

[14] Samakovlis C, Hacohen N, Manning G, Sutherland DC, Guillemin K, et al (1996) Development of the Drosophila tracheal system occurs by a series of morphologically distinct but genetically coupled branching events. Development 122: 1395–1407. 8625828 10.1242/dev.122.5.1395

[15] Wegener C, Gorbashov A (2008) Molecular evolution of neuropeptides in the genus Drosophila. Genome biology 9: R131. 18717992 10.1186/gb-2008-9-8-r131 PMC2575521

[16] Clark AC, del Campo ML, Ewer J (2004) Neuroendocrine control of larval ecdysis behavior in Drosophila: complex regulation by partially redundant neuropeptides. J Neurosci 24: 4283–4292. 15115824 10.1523/JNEUROSCI.4938-03.2004 PMC6729283

[17] Pitsouli C, Perrimon N (2010) Embryonic multipotent progenitors remodel the Drosophila airways during metamorphosis. Development 137: 3615–3624. 20940225 10.1242/dev.056408 PMC2964094

[18] Weaver M, Krasnow MA (2008) Dual origin of tissue-specific progenitor cells in Drosophila tracheal remodeling. Science 321: 1496–1499. 18669822 10.1126/science.1158712 PMC3999966

[19] Abdelsadik A, Roeder T (2010) Chronic activation of the epithelial immune system of the fruit fly’s salivary glands has a negative effect on organismal growth and induces a peculiar set of target genes. BMC genomics 11: 265. 20420686 10.1186/1471-2164-11-265 PMC2874812

[20] Schramm G, Bruchhaus I, Roeder T (2000) A simple and reliable 5′-RACE approach. Nucleic Acids Res 28: E96. 11071950 10.1093/nar/28.22.e96 PMC113888

[21] Sharma Y, Cheung U, Larsen EW, Eberl DF (2002) PPTGAL, a convenient Gal4 P-element vector for testing expression of enhancer fragments in drosophila. Genesis 34: 115–118. 12324963 10.1002/gene.10127 PMC1805626

[22] Huang da W, Sherman BT, Zheng X, Yang J, Imamichi T, et al.. (2009) Extracting biological meaning from large gene lists with DAVID. Curr Protoc Bioinformatics Chapter 13: Unit 13 11. 10.1002/0471250953.bi1311s27 19728287

[23] Wegener C, Herbert H, Kahnt J, Bender M, Rhea JM (2011) Deficiency of prohormone convertase dPC2 (AMONTILLADO) results in impaired production of bioactive neuropeptide hormones in Drosophila. Journal of neurochemistry 118: 581–595. 21138435 10.1111/j.1471-4159.2010.07130.x

[24] Gervais L, Casanova J (2011) The Drosophila homologue of SRF acts as a boosting mechanism to sustain FGF-induced terminal branching in the tracheal system. Development 138: 1269–1274. 21385762 10.1242/dev.059188

[25] Roeder T (2005) Tyramine and octopamine: ruling behavior and metabolism. Annual review of entomology 50: 447–477. 10.1146/annurev.ento.50.071803.130404 15355245

[26] Kida H, Mucenski ML, Thitoff AR, Le Cras TD, Park KS, et al (2008) GP130-STAT3 regulates epithelial cell migration and is required for repair of the bronchiolar epithelium. Am J Pathol 172: 1542–1554. 18467707 10.2353/ajpath.2008.071052 PMC2408415

[27] Liedtke CM (1989) Alpha-adrenergic regulation of Na-Cl cotransport in human airway epithelium. The American journal of physiology 257: L125–129. 2548396 10.1152/ajplung.1989.257.2.L125

[28] Salathe M (2002) Effects of beta-agonists on airway epithelial cells. The Journal of allergy and clinical immunology 110: S275–281. 12464936 10.1067/mai.2002.129412

[29] Costantino BF, Bricker DK, Alexandre K, Shen K, Merriam JR, et al (2008) A novel ecdysone receptor mediates steroid-regulated developmental events during the mid-third instar of Drosophila. PLoS Genet 4: e1000102. 18566664 10.1371/journal.pgen.1000102 PMC2413497

[30] Robinson SW, Herzyk P, Dow JA, Leader DP (2013) FlyAtlas: database of gene expression in the tissues of Drosophila melanogaster. Nucleic acids research 41: D744–750. 23203866 10.1093/nar/gks1141 PMC3531048

[31] Roeder T (2002) Biochemistry and molecular biology of receptors for biogenic amines in locusts. Microsc Res Tech 56: 237–247. 11810725 10.1002/jemt.10027

[32] Roeder T (1999) Octopamine in invertebrates. Prog Neurobiol 59: 533–561. 10515667 10.1016/s0301-0082(99)00016-7

[33] Seiler F, Hellberg J, Lepper PM, Kamyschnikow A, Herr C, et al (2013) FOXO Transcription Factors Regulate Innate Immune Mechanisms in Respiratory Epithelial Cells. J Immunol 190: 1603–1613. 23315071 10.4049/jimmunol.1200596

[34] Meeusen T, Mertens I, Clynen E, Baggerman G, Nichols R, et al (2002) Identification in Drosophila melanogaster of the invertebrate G protein-coupled FMRFamide receptor. Proc Natl Acad Sci U S A 99: 15363–15368. 12438685 10.1073/pnas.252339599 PMC137722

[35] Vermeer PD, Einwalter LA, Moninger TO, Rokhlina T, Kern JA, et al (2003) Segregation of receptor and ligand regulates activation of epithelial growth factor receptor. Nature 422: 322–326. 12646923 10.1038/nature01440

[36] Witte I, Kreienkamp HJ, Gewecke M, Roeder T (2002) Putative histamine-gated chloride channel subunits of the insect visual system and thoracic ganglion. Journal of neurochemistry 83: 504–514. 12390512 10.1046/j.1471-4159.2002.01076.x

[37] Terakado M, Gon Y, Sekiyama A, Takeshita I, Kozu Y, et al (2011) The Rac1/JNK pathway is critical for EGFR-dependent barrier formation in human airway epithelial cells. Am J Physiol Lung Cell Mol Physiol 300: L56–63. 21036915 10.1152/ajplung.00159.2010

[38] Hashimoto S, Chen H, Que J, Brockway BL, Drake JA, et al (2012) beta-Catenin-SOX2 signaling regulates the fate of developing airway epithelium. J Cell Sci 125: 932–942. 22421361 10.1242/jcs.092734 PMC3311930

[39] Brun S, Vidal S, Spellman P, Takahashi K, Tricoire H, et al (2006) The MAPKKK Mekk1 regulates the expression of Turandot stress genes in response to septic injury in Drosophila. Genes Cells 11: 397–407. 16611243 10.1111/j.1365-2443.2006.00953.x

[40] Ekengren S, Hultmark D (2001) A family of Turandot-related genes in the humoral stress response of Drosophila. Biochem Biophys Res Commun 284: 998–1003. 11409894 10.1006/bbrc.2001.5067

