# Supplementary figures and images for: Transcriptional Regionalization of the Fruit Fly’s Airway Epithelium
Source: PLoS One. 2014 Jul 14;9(7):e102534. doi: 10.1371/journal.pone.0102534 (PMC4097054; doi:10.1371/journal.pone.0102534)

## Slide 1
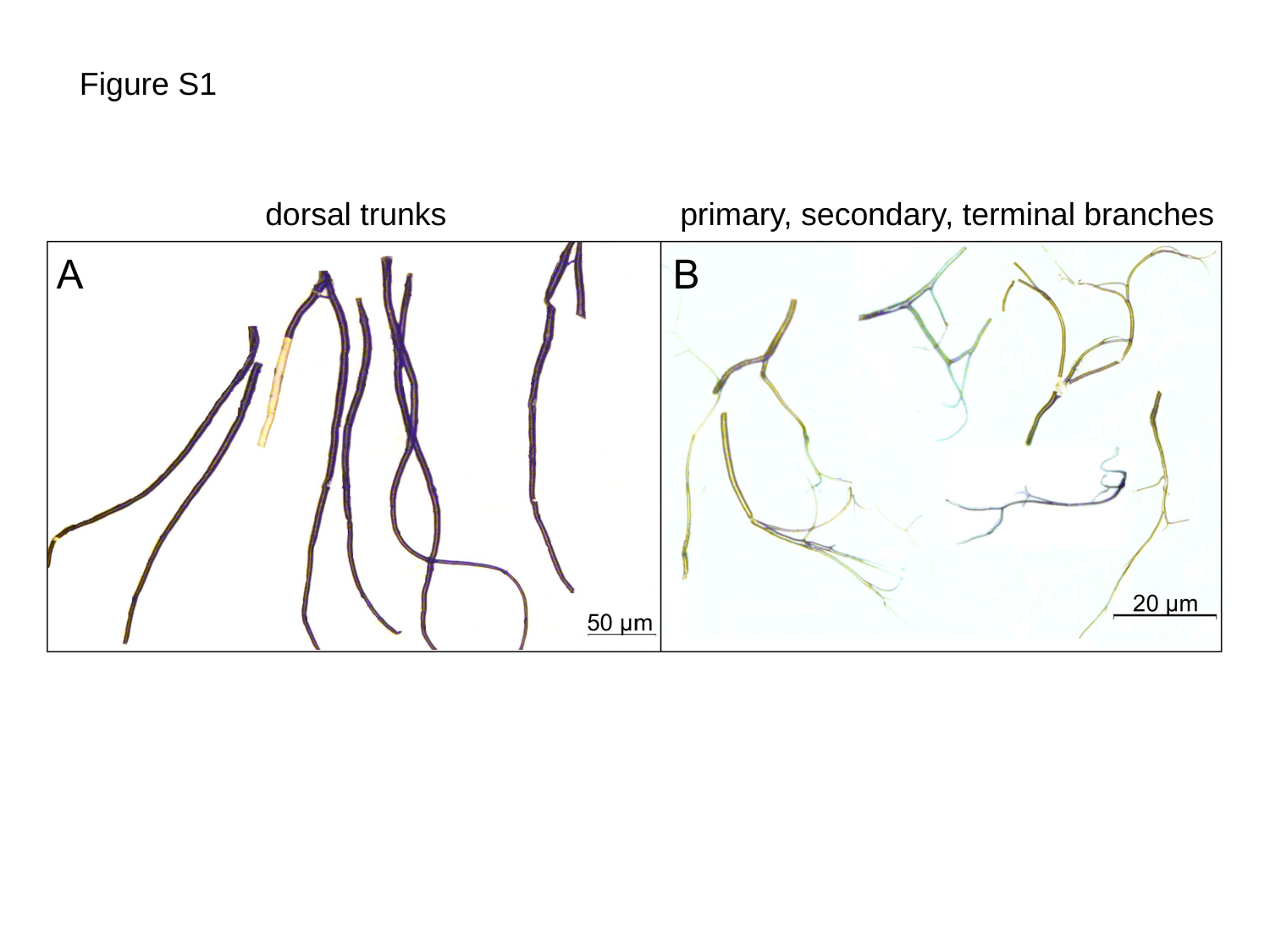

Figure S1
primary, secondary, terminal branches
dorsal trunks

Supplement: Figure S1 — Shown are typical examples of dorsal trunks (A), and primary, secondary, terminal branches (B) manually isolated from 3rd instar larvae that were used for downstream experiments. (PPTX) [file pone.0102534.s001.pptx]
