# Supplementary material for: Transcriptional Regionalization of the Fruit Fly’s Airway Epithelium
Source: PLoS One. 2014 Jul 14;9(7):e102534. doi: 10.1371/journal.pone.0102534 (PMC4097054; doi:10.1371/journal.pone.0102534)
Supplement: Table S1 — Genes with 1.5 fold higher expression in dorsal trunks. (DOCX) [file pone.0102534.s002.docx]

**Table S1:**

**Genes with 1.5 fold higher expression in dorsal trunks (in 2 out of three independent experiments)**

| **FBID KEY** | **NAME** | **SYMBOL** |
| --- | --- | --- |
| [FBgn0000964](http://flybase.org/cgi-bin/fbidq.html?FBgn0000964) | traffic jam | [tj](http://flybase.org/cgi-bin/fbidq.html?FBgn0000964) |
| [FBgn0038006](http://flybase.org/cgi-bin/fbidq.html?FBgn0038006) | Cyp313a2 | [Cyp313a2](http://flybase.org/cgi-bin/fbidq.html?FBgn0038006) |
| [FBgn0035713](http://flybase.org/cgi-bin/fbidq.html?FBgn0035713) | veloren | [velo](http://flybase.org/cgi-bin/fbidq.html?FBgn0035713) |
| [FBgn0037502](http://flybase.org/cgi-bin/fbidq.html?FBgn0037502) | walker cup | [wa-cup](http://flybase.org/cgi-bin/fbidq.html?FBgn0037502) |
| FBgn0005626 | pale | ple |
| [FBgn0028554](http://flybase.org/cgi-bin/fbidq.html?FBgn0028554) | - | [x16](http://flybase.org/cgi-bin/fbidq.html?FBgn0028554) |
| [FBgn0033979](http://flybase.org/cgi-bin/fbidq.html?FBgn0033979) | Cyp6a19 | [Cyp6a19](http://flybase.org/cgi-bin/fbidq.html?FBgn0033979) |
| [FBgn0027596](http://flybase.org/cgi-bin/fbidq.html?FBgn0027596) | - | [CG10249](http://flybase.org/cgi-bin/fbidq.html?FBgn0027596) |
| [FBgn0010282](http://flybase.org/cgi-bin/fbidq.html?FBgn0010282) | Transcription factor IIFalpha | [TfIIFalpha](http://flybase.org/cgi-bin/fbidq.html?FBgn0010282) |
| [FBgn0037412](http://flybase.org/cgi-bin/fbidq.html?FBgn0037412) | Osiris 4 | [Osi4](http://flybase.org/cgi-bin/fbidq.html?FBgn0037412) |
| [FBgn0035678](http://flybase.org/cgi-bin/fbidq.html?FBgn0035678) | - | [CG10469](http://flybase.org/cgi-bin/fbidq.html?FBgn0035678) |
| [FBgn0041775](http://flybase.org/cgi-bin/fbidq.html?FBgn0041775) | trailer hitch | [tral](http://flybase.org/cgi-bin/fbidq.html?FBgn0041775) |
| [FBgn0032845](http://flybase.org/cgi-bin/fbidq.html?FBgn0032845) | - | [CG10747](http://flybase.org/cgi-bin/fbidq.html?FBgn0032845) |
| [FBgn0029656](http://flybase.org/cgi-bin/fbidq.html?FBgn0029656) | - | [CG10793](http://flybase.org/cgi-bin/fbidq.html?FBgn0029656) |
| [FBgn0017551](http://flybase.org/cgi-bin/fbidq.html?FBgn0017551) | Regulator of cyclin A1 | [Rca1](http://flybase.org/cgi-bin/fbidq.html?FBgn0017551) |
| [FBgn0033876](http://flybase.org/cgi-bin/fbidq.html?FBgn0033876) | Synaptogyrin | [Syngr](http://flybase.org/cgi-bin/fbidq.html?FBgn0033876) |
| [FBgn0035434](http://flybase.org/cgi-bin/fbidq.html?FBgn0035434) | Drosomycin-like 5 | [Drsl5](http://flybase.org/cgi-bin/fbidq.html?FBgn0035434) |
| [FBgn0020660](http://flybase.org/cgi-bin/fbidq.html?FBgn0020660) | Eukaryotic initiation factor 4B | [eIF-4B](http://flybase.org/cgi-bin/fbidq.html?FBgn0020660) |
| [FBgn0028858](http://flybase.org/cgi-bin/fbidq.html?FBgn0028858) | - | [CG10839](http://flybase.org/cgi-bin/fbidq.html?FBgn0028858) |
| [FBgn0004587](http://flybase.org/cgi-bin/fbidq.html?FBgn0004587) | B52 | [B52](http://flybase.org/cgi-bin/fbidq.html?FBgn0004587) |
| [FBgn0019686](http://flybase.org/cgi-bin/fbidq.html?FBgn0019686) | loki | [lok](http://flybase.org/cgi-bin/fbidq.html?FBgn0019686) |
| [FBgn0014007](http://flybase.org/cgi-bin/fbidq.html?FBgn0014007) | Protein tyrosine phosphatase 69D | [Ptp69D](http://flybase.org/cgi-bin/fbidq.html?FBgn0014007) |
| [FBgn0027930](http://flybase.org/cgi-bin/fbidq.html?FBgn0027930) | Melanization Protease 1 | [MP1](http://flybase.org/cgi-bin/fbidq.html?FBgn0027930) |
| [FBgn0030241](http://flybase.org/cgi-bin/fbidq.html?FBgn0030241) | fascetto | [feo](http://flybase.org/cgi-bin/fbidq.html?FBgn0030241) |
| [FBgn0020372](http://flybase.org/cgi-bin/fbidq.html?FBgn0020372) | Transmembrane 4 superfamily | [TM4SF](http://flybase.org/cgi-bin/fbidq.html?FBgn0020372) |
| [FBgn0031214](http://flybase.org/cgi-bin/fbidq.html?FBgn0031214) | - | [CG11374](http://flybase.org/cgi-bin/fbidq.html?FBgn0031214) |
| [FBgn0038732](http://flybase.org/cgi-bin/fbidq.html?FBgn0038732) | - | [CG11391](http://flybase.org/cgi-bin/fbidq.html?FBgn0038732) |
| FBgn0003210 | Ruby | rb |
| [FBgn0038037](http://flybase.org/cgi-bin/fbidq.html?FBgn0038037) | Cyp9f2 | [Cyp9f2](http://flybase.org/cgi-bin/fbidq.html?FBgn0038037) |
| FBgn0259994 | - | CG42492 |
| FBgn82582 | Tropomodulin | tmod |
| [FBgn0001170](http://flybase.org/cgi-bin/fbidq.html?FBgn0001170) | Homeodomain protein 2.0 | [H2.0](http://flybase.org/cgi-bin/fbidq.html?FBgn0001170) |
| [FBgn0002945](http://flybase.org/cgi-bin/fbidq.html?FBgn0002945) | naked cuticle | [nkd](http://flybase.org/cgi-bin/fbidq.html?FBgn0002945) |
| [FBgn0004430](http://flybase.org/cgi-bin/fbidq.html?FBgn0004430) | Lysozyme S | [LysS](http://flybase.org/cgi-bin/fbidq.html?FBgn0004430) |
| [FBgn0040648](http://flybase.org/cgi-bin/fbidq.html?FBgn0040648) | - | [CG11666](http://flybase.org/cgi-bin/fbidq.html?FBgn0040648) |
| [FBgn0003373](http://flybase.org/cgi-bin/fbidq.html?FBgn0003373) | Salivary gland secretion 3 | [Sgs3](http://flybase.org/cgi-bin/fbidq.html?FBgn0003373) |
| [FBgn0030294](http://flybase.org/cgi-bin/fbidq.html?FBgn0030294) | PTIP associated 1 | [Pa1](http://flybase.org/cgi-bin/fbidq.html?FBgn0030294) |
| [FBgn0039252](http://flybase.org/cgi-bin/fbidq.html?FBgn0039252) | - | [CG11771](http://flybase.org/cgi-bin/fbidq.html?FBgn0039252) |
| [FBgn0015371](http://flybase.org/cgi-bin/fbidq.html?FBgn0015371) | charlatan | [chn](http://flybase.org/cgi-bin/fbidq.html?FBgn0015371) |
| [FBgn0039271](http://flybase.org/cgi-bin/fbidq.html?FBgn0039271) | - | [CG11839](http://flybase.org/cgi-bin/fbidq.html?FBgn0039271) |
| [FBgn0261268](http://flybase.org/cgi-bin/fbidq.html?FBgn0261268) | Cullin-3 | [Cul-3](http://flybase.org/cgi-bin/fbidq.html?FBgn0261268) |
| [FBgn0033540](http://flybase.org/cgi-bin/fbidq.html?FBgn0033540) | Elongator complex protein 2 | [Elp2](http://flybase.org/cgi-bin/fbidq.html?FBgn0033540) |
| [FBgn0004897](http://flybase.org/cgi-bin/fbidq.html?FBgn0004897) | forkhead domain 96Ca | [fd96Ca](http://flybase.org/cgi-bin/fbidq.html?FBgn0004897) |
| [FBgn0039656](http://flybase.org/cgi-bin/fbidq.html?FBgn0039656) | - | [CG11951](http://flybase.org/cgi-bin/fbidq.html?FBgn0039656) |
| [FBgn0037656](http://flybase.org/cgi-bin/fbidq.html?FBgn0037656) | - | [CG11986](http://flybase.org/cgi-bin/fbidq.html?FBgn0037656) |
| [FBgn0027790](http://flybase.org/cgi-bin/fbidq.html?FBgn0027790) | GV1 | [GV1](http://flybase.org/cgi-bin/fbidq.html?FBgn0027790) |
| [FBgn0027084](http://flybase.org/cgi-bin/fbidq.html?FBgn0027084) | Lysyl-tRNA synthetase | [Aats-lys](http://flybase.org/cgi-bin/fbidq.html?FBgn0027084) |
| [FBgn0016041](http://flybase.org/cgi-bin/fbidq.html?FBgn0016041) | Translocase of outer membrane 40 | [Tom40](http://flybase.org/cgi-bin/fbidq.html?FBgn0016041) |
| [FBgn0003374](http://flybase.org/cgi-bin/fbidq.html?FBgn0003374) | Salivary gland secretion 4 | [Sgs4](http://flybase.org/cgi-bin/fbidq.html?FBgn0003374) |
| [FBgn0086368](http://flybase.org/cgi-bin/fbidq.html?FBgn0086368) | Protein O-mannosyltransferase 2 | [tw](http://flybase.org/cgi-bin/fbidq.html?FBgn0086368) |
| [FBgn0038558](http://flybase.org/cgi-bin/fbidq.html?FBgn0038558) | - | [CG12347](http://flybase.org/cgi-bin/fbidq.html?FBgn0038558) |
| [FBgn0024188](http://flybase.org/cgi-bin/fbidq.html?FBgn0024188) | separation anxiety | [san](http://flybase.org/cgi-bin/fbidq.html?FBgn0024188) |
| [FBgn0031768](http://flybase.org/cgi-bin/fbidq.html?FBgn0031768) | - | [CG12393](http://flybase.org/cgi-bin/fbidq.html?FBgn0031768) |
| [FBgn0033500](http://flybase.org/cgi-bin/fbidq.html?FBgn0033500) | - | [CG12913](http://flybase.org/cgi-bin/fbidq.html?FBgn0033500) |
| [FBgn0037061](http://flybase.org/cgi-bin/fbidq.html?FBgn0037061) | - | [CG12975](http://flybase.org/cgi-bin/fbidq.html?FBgn0037061) |
| [FBgn0036596](http://flybase.org/cgi-bin/fbidq.html?FBgn0036596) | - | [CG13045](http://flybase.org/cgi-bin/fbidq.html?FBgn0036596) |
| [FBgn0036586](http://flybase.org/cgi-bin/fbidq.html?FBgn0036586) | - | [CG13070](http://flybase.org/cgi-bin/fbidq.html?FBgn0036586) |
| [FBgn0036577](http://flybase.org/cgi-bin/fbidq.html?FBgn0036577) | - | [CG13073](http://flybase.org/cgi-bin/fbidq.html?FBgn0036577) |
| [FBgn0032054](http://flybase.org/cgi-bin/fbidq.html?FBgn0032054) | - | [CG13090](http://flybase.org/cgi-bin/fbidq.html?FBgn0032054) |
| [FBgn0260932](http://flybase.org/cgi-bin/fbidq.html?FBgn0260932) | cutoff | [cuff](http://flybase.org/cgi-bin/fbidq.html?FBgn0260932) |
| [FBgn0035931](http://flybase.org/cgi-bin/fbidq.html?FBgn0035931) | - | [CG13312](http://flybase.org/cgi-bin/fbidq.html?FBgn0035931) |
| [FBgn0033863](http://flybase.org/cgi-bin/fbidq.html?FBgn0033863) | - | [CG13337](http://flybase.org/cgi-bin/fbidq.html?FBgn0033863) |
| [FBgn0029529](http://flybase.org/cgi-bin/fbidq.html?FBgn0029529) | - | [CG13365](http://flybase.org/cgi-bin/fbidq.html?FBgn0029529) |
| [FBgn0262002](http://flybase.org/cgi-bin/fbidq.html?FBgn0262002) | - | [CG42820](http://flybase.org/cgi-bin/fbidq.html?FBgn0262002) |
| [FBgn0027780](http://flybase.org/cgi-bin/fbidq.html?FBgn0027780) | U26 | [U26](http://flybase.org/cgi-bin/fbidq.html?FBgn0027780) |
| [FBgn0034546](http://flybase.org/cgi-bin/fbidq.html?FBgn0034546) | - | [CG13442](http://flybase.org/cgi-bin/fbidq.html?FBgn0034546) |
| [FBgn0039239](http://flybase.org/cgi-bin/fbidq.html?FBgn0039239) | - | [CG13641](http://flybase.org/cgi-bin/fbidq.html?FBgn0039239) |
| [FBgn0039294](http://flybase.org/cgi-bin/fbidq.html?FBgn0039294) | Cadherin 96Cb | [Cad96Cb](http://flybase.org/cgi-bin/fbidq.html?FBgn0039294) |
| FBgn0031888 | PDGF- and VEGF-related factor 2 | Pvf2 |
| [FBgn0261556](http://flybase.org/cgi-bin/fbidq.html?FBgn0261556) | - | [CG42674](http://flybase.org/cgi-bin/fbidq.html?FBgn0261556) |
| [FBgn0263117](http://flybase.org/cgi-bin/fbidq.html?FBgn0263117) | - | [CG34377](http://flybase.org/cgi-bin/fbidq.html?FBgn0263117) |
| [FBgn0085410](http://flybase.org/cgi-bin/fbidq.html?FBgn0085410) | Trissin receptor | [TrissinR](http://flybase.org/cgi-bin/fbidq.html?FBgn0085410) |
| [FBgn0016076](http://flybase.org/cgi-bin/fbidq.html?FBgn0016076) | vrille | [vri](http://flybase.org/cgi-bin/fbidq.html?FBgn0016076) |
| [FBgn0036690](http://flybase.org/cgi-bin/fbidq.html?FBgn0036690) | Insulin-like peptide 8 | [Ilp8](http://flybase.org/cgi-bin/fbidq.html?FBgn0036690) |
| [FBgn0036192](http://flybase.org/cgi-bin/fbidq.html?FBgn0036192) | Pallidin ortholog (H. sapiens) | [Pallidin](http://flybase.org/cgi-bin/fbidq.html?FBgn0036192) |
| [FBgn0036193](http://flybase.org/cgi-bin/fbidq.html?FBgn0036193) | - | [CG14135](http://flybase.org/cgi-bin/fbidq.html?FBgn0036193) |
| [FBgn0031062](http://flybase.org/cgi-bin/fbidq.html?FBgn0031062) | - | [CG14230](http://flybase.org/cgi-bin/fbidq.html?FBgn0031062) |
| [FBgn0039428](http://flybase.org/cgi-bin/fbidq.html?FBgn0039428) | - | [CG14237](http://flybase.org/cgi-bin/fbidq.html?FBgn0039428) |
| [FBgn0038596](http://flybase.org/cgi-bin/fbidq.html?FBgn0038596) | - | [CG14312](http://flybase.org/cgi-bin/fbidq.html?FBgn0038596) |
| [FBgn0029880](http://flybase.org/cgi-bin/fbidq.html?FBgn0029880) | - | [CG14443](http://flybase.org/cgi-bin/fbidq.html?FBgn0029880) |
| [FBgn0085414](http://flybase.org/cgi-bin/fbidq.html?FBgn0085414) | dpr12 | [dpr12](http://flybase.org/cgi-bin/fbidq.html?FBgn0085414) |
| [FBgn0038342](http://flybase.org/cgi-bin/fbidq.html?FBgn0038342) | - | [CG14870](http://flybase.org/cgi-bin/fbidq.html?FBgn0038342) |
| [FBgn0035414](http://flybase.org/cgi-bin/fbidq.html?FBgn0035414) | - | [CG14965](http://flybase.org/cgi-bin/fbidq.html?FBgn0035414) |
| [FBgn0035480](http://flybase.org/cgi-bin/fbidq.html?FBgn0035480) | - | [CG14984](http://flybase.org/cgi-bin/fbidq.html?FBgn0035480) |
| [FBgn0035515](http://flybase.org/cgi-bin/fbidq.html?FBgn0035515) | - | [CG14997](http://flybase.org/cgi-bin/fbidq.html?FBgn0035515) |
| [FBgn0260985](http://flybase.org/cgi-bin/fbidq.html?FBgn0260985) | Replication factor C subunit 4 | [RfC4](http://flybase.org/cgi-bin/fbidq.html?FBgn0260985) |
| [FBgn0041171](http://flybase.org/cgi-bin/fbidq.html?FBgn0041171) | archipelago | [ago](http://flybase.org/cgi-bin/fbidq.html?FBgn0041171) |
| [FBgn0035518](http://flybase.org/cgi-bin/fbidq.html?FBgn0035518) | - | [CG15011](http://flybase.org/cgi-bin/fbidq.html?FBgn0035518) |
| [FBgn0035543](http://flybase.org/cgi-bin/fbidq.html?FBgn0035543) | - | [CG15020](http://flybase.org/cgi-bin/fbidq.html?FBgn0035543) |
| [FBgn0034396](http://flybase.org/cgi-bin/fbidq.html?FBgn0034396) | - | [CG15097](http://flybase.org/cgi-bin/fbidq.html?FBgn0034396) |
| [FBgn0032732](http://flybase.org/cgi-bin/fbidq.html?FBgn0032732) | - | [CG15168](http://flybase.org/cgi-bin/fbidq.html?FBgn0032732) |
| [FBgn0032740](http://flybase.org/cgi-bin/fbidq.html?FBgn0032740) | - | [CG15172](http://flybase.org/cgi-bin/fbidq.html?FBgn0032740) |
| [FBgn0053543](http://flybase.org/cgi-bin/fbidq.html?FBgn0053543) | - | [CG33543](http://flybase.org/cgi-bin/fbidq.html?FBgn0053543) |
| [FBgn0040717](http://flybase.org/cgi-bin/fbidq.html?FBgn0040717) | Neuropeptide-like precursor 4 | [Nplp4](http://flybase.org/cgi-bin/fbidq.html?FBgn0040717) |
| [FBgn0031523](http://flybase.org/cgi-bin/fbidq.html?FBgn0031523) | - | [CG15408](http://flybase.org/cgi-bin/fbidq.html?FBgn0031523) |
| [FBgn0033225](http://flybase.org/cgi-bin/fbidq.html?FBgn0033225) | - | [CG1550](http://flybase.org/cgi-bin/fbidq.html?FBgn0033225) |
| [FBgn0261803](http://flybase.org/cgi-bin/fbidq.html?FBgn0261803) | - | [CG42749](http://flybase.org/cgi-bin/fbidq.html?FBgn0261803) |
| [FBgn0040034](http://flybase.org/cgi-bin/fbidq.html?FBgn0040034) | - | [CG15831](http://flybase.org/cgi-bin/fbidq.html?FBgn0040034) |
| FBgn0004910 | Ecdyson-induced protein 63F-1 | Eip63F-1 |
| [FBgn0036909](http://flybase.org/cgi-bin/fbidq.html?FBgn0036909) | - | [CG15881](http://flybase.org/cgi-bin/fbidq.html?FBgn0036909) |
| [FBgn0030985](http://flybase.org/cgi-bin/fbidq.html?FBgn0030985) | Odorant-binding protein 18a | [Obp18a](http://flybase.org/cgi-bin/fbidq.html?FBgn0030985) |
| FBgn0259745 | wech | wech |
| [FBgn0040092](http://flybase.org/cgi-bin/fbidq.html?FBgn0040092) | lectin-46Cb | [lectin-46Cb](http://flybase.org/cgi-bin/fbidq.html?FBgn0040092) |
| [FBgn0030481](http://flybase.org/cgi-bin/fbidq.html?FBgn0030481) | - | [CG1662](http://flybase.org/cgi-bin/fbidq.html?FBgn0030481) |
| [FBgn0037678](http://flybase.org/cgi-bin/fbidq.html?FBgn0037678) | - | [CG16749](http://flybase.org/cgi-bin/fbidq.html?FBgn0037678) |
| [FBgn0032779](http://flybase.org/cgi-bin/fbidq.html?FBgn0032779) | - | [CG16771](http://flybase.org/cgi-bin/fbidq.html?FBgn0032779) |
| [FBgn0032505](http://flybase.org/cgi-bin/fbidq.html?FBgn0032505) | - | [CG16826](http://flybase.org/cgi-bin/fbidq.html?FBgn0032505) |
| [FBgn0085465](http://flybase.org/cgi-bin/fbidq.html?FBgn0085465) | - | [CG34436](http://flybase.org/cgi-bin/fbidq.html?FBgn0085465) |
| FBgn0025637 | skpA | skpA |
| [FBgn0025740](http://flybase.org/cgi-bin/fbidq.html?FBgn0025740) | plexin B | [plexB](http://flybase.org/cgi-bin/fbidq.html?FBgn0025740) |
| FBgn0035626 | lin-28 | lin-28 |
| [FBgn0086446](http://flybase.org/cgi-bin/fbidq.html?FBgn0086446) | lethal (2) 37Ce | [l(2)37Ce](http://flybase.org/cgi-bin/fbidq.html?FBgn0086446) |
| [FBgn0264006](http://flybase.org/cgi-bin/fbidq.html?FBgn0264006) | dyschronic | [dysc](http://flybase.org/cgi-bin/fbidq.html?FBgn0264006) |
| [FBgn0031145](http://flybase.org/cgi-bin/fbidq.html?FBgn0031145) | Nuclear transport factor-2 | [Ntf-2](http://flybase.org/cgi-bin/fbidq.html?FBgn0031145) |
| [FBgn0064225](http://flybase.org/cgi-bin/fbidq.html?FBgn0064225) | Ribosomal protein L5 | [RpL5](http://flybase.org/cgi-bin/fbidq.html?FBgn0064225) |
| [FBgn0040011](http://flybase.org/cgi-bin/fbidq.html?FBgn0040011) | - | [CG17494](http://flybase.org/cgi-bin/fbidq.html?FBgn0040011) |
| [FBgn0032774](http://flybase.org/cgi-bin/fbidq.html?FBgn0032774) | - | [CG17549](http://flybase.org/cgi-bin/fbidq.html?FBgn0032774) |
| [FBgn0031364](http://flybase.org/cgi-bin/fbidq.html?FBgn0031364) | - | [CG17648](http://flybase.org/cgi-bin/fbidq.html?FBgn0031364) |
| [FBgn0034352](http://flybase.org/cgi-bin/fbidq.html?FBgn0034352) | - | [CG17669](http://flybase.org/cgi-bin/fbidq.html?FBgn0034352) |
| [FBgn0038009](http://flybase.org/cgi-bin/fbidq.html?FBgn0038009) | - | [CG17738](http://flybase.org/cgi-bin/fbidq.html?FBgn0038009) |
| [FBgn0004907](http://flybase.org/cgi-bin/fbidq.html?FBgn0004907) | 14-3-3zeta | [14-3-3zeta](http://flybase.org/cgi-bin/fbidq.html?FBgn0004907) |
| [FBgn0264953](http://flybase.org/cgi-bin/fbidq.html?FBgn0264953) | Piezo | [Piezo](http://flybase.org/cgi-bin/fbidq.html?FBgn0264953) |
| [FBgn0036038](http://flybase.org/cgi-bin/fbidq.html?FBgn0036038) | deflated | [defl](http://flybase.org/cgi-bin/fbidq.html?FBgn0036038) |
| FBgn0038470 | - | CG18213 |
| [FBgn0036795](http://flybase.org/cgi-bin/fbidq.html?FBgn0036795) | - | [CG18233](http://flybase.org/cgi-bin/fbidq.html?FBgn0036795) |
| [FBgn0033524](http://flybase.org/cgi-bin/fbidq.html?FBgn0033524) | Cyp49a1 | [Cyp49a1](http://flybase.org/cgi-bin/fbidq.html?FBgn0033524) |
| [FBgn0030351](http://flybase.org/cgi-bin/fbidq.html?FBgn0030351) | - | [CG1840](http://flybase.org/cgi-bin/fbidq.html?FBgn0030351) |
| [FBgn0034217](http://flybase.org/cgi-bin/fbidq.html?FBgn0034217) | Lethal hybrid rescue | [Lhr](http://flybase.org/cgi-bin/fbidq.html?FBgn0034217) |
| [FBgn0034326](http://flybase.org/cgi-bin/fbidq.html?FBgn0034326) | - | [CG18540](http://flybase.org/cgi-bin/fbidq.html?FBgn0034326) |
| [FBgn0250910](http://flybase.org/cgi-bin/fbidq.html?FBgn0250910) | Octopamine beta3 receptor | [Octbeta3R](http://flybase.org/cgi-bin/fbidq.html?FBgn0250910) |
| [FBgn0042135](http://flybase.org/cgi-bin/fbidq.html?FBgn0042135) | - | [CG18812](http://flybase.org/cgi-bin/fbidq.html?FBgn0042135) |
| [FBgn0005648](http://flybase.org/cgi-bin/fbidq.html?FBgn0005648) | Pabp2 | [Pabp2](http://flybase.org/cgi-bin/fbidq.html?FBgn0005648) |
| [FBgn0033273](http://flybase.org/cgi-bin/fbidq.html?FBgn0033273) | GASZ ortholog | [Gasz](http://flybase.org/cgi-bin/fbidq.html?FBgn0033273) |
| [FBgn0035210](http://flybase.org/cgi-bin/fbidq.html?FBgn0035210) | mitotic spindle density 5 | [msd5](http://flybase.org/cgi-bin/fbidq.html?FBgn0035210) |
| [FBgn0011770](http://flybase.org/cgi-bin/fbidq.html?FBgn0011770) | GIP-like | [Gip](http://flybase.org/cgi-bin/fbidq.html?FBgn0011770) |
| [FBgn0000715](http://flybase.org/cgi-bin/fbidq.html?FBgn0000715) | FMRFamide | [FMRFa](http://flybase.org/cgi-bin/fbidq.html?FBgn0000715) |
| [FBgn0030319](http://flybase.org/cgi-bin/fbidq.html?FBgn0030319) | - | [CG2533](http://flybase.org/cgi-bin/fbidq.html?FBgn0030319) |
| [FBgn0015801](http://flybase.org/cgi-bin/fbidq.html?FBgn0015801) | Rhythmically expressed gene 5 | [Reg-5](http://flybase.org/cgi-bin/fbidq.html?FBgn0015801) |
| [FBgn0028665](http://flybase.org/cgi-bin/fbidq.html?FBgn0028665) | Vacuolar H[+] ATPase AC39 subunit 1 | [VhaAC39-1](http://flybase.org/cgi-bin/fbidq.html?FBgn0028665) |
| [FBgn0026238](http://flybase.org/cgi-bin/fbidq.html?FBgn0026238) | gustavus | [gus](http://flybase.org/cgi-bin/fbidq.html?FBgn0026238) |
| [FBgn0030178](http://flybase.org/cgi-bin/fbidq.html?FBgn0030178) | - | [CG2974](http://flybase.org/cgi-bin/fbidq.html?FBgn0030178) |
| [FBgn0050007](http://flybase.org/cgi-bin/fbidq.html?FBgn0050007) | - | [CG30007](http://flybase.org/cgi-bin/fbidq.html?FBgn0050007) |
| [FBgn0050015](http://flybase.org/cgi-bin/fbidq.html?FBgn0050015) | - | [CG30015](http://flybase.org/cgi-bin/fbidq.html?FBgn0050015) |
| [FBgn0050016](http://flybase.org/cgi-bin/fbidq.html?FBgn0050016) | - | [CG30016](http://flybase.org/cgi-bin/fbidq.html?FBgn0050016) |
| [FBgn0050033](http://flybase.org/cgi-bin/fbidq.html?FBgn0050033) | - | [CG30033](http://flybase.org/cgi-bin/fbidq.html?FBgn0050033) |
| [FBgn0264691](http://flybase.org/cgi-bin/fbidq.html?FBgn0264691) | - | [Lst8](http://flybase.org/cgi-bin/fbidq.html?FBgn0264691) |
| [FBgn0050094](http://flybase.org/cgi-bin/fbidq.html?FBgn0050094) | - | [CG30094](http://flybase.org/cgi-bin/fbidq.html?FBgn0050094) |
| [FBgn0050101](http://flybase.org/cgi-bin/fbidq.html?FBgn0050101) | - | [CG30101](http://flybase.org/cgi-bin/fbidq.html?FBgn0050101) |
| [FBgn0050183](http://flybase.org/cgi-bin/fbidq.html?FBgn0050183) | - | [CG30183](http://flybase.org/cgi-bin/fbidq.html?FBgn0050183) |
| [FBgn0050352](http://flybase.org/cgi-bin/fbidq.html?FBgn0050352) | - | [CG30352](http://flybase.org/cgi-bin/fbidq.html?FBgn0050352) |
| [FBgn0050440](http://flybase.org/cgi-bin/fbidq.html?FBgn0050440) | - | [CG30440](http://flybase.org/cgi-bin/fbidq.html?FBgn0050440) |
| [FBgn0026319](http://flybase.org/cgi-bin/fbidq.html?FBgn0026319) | TNF-receptor-associated factor 4 | [Traf4](http://flybase.org/cgi-bin/fbidq.html?FBgn0026319) |
| [FBgn0051104](http://flybase.org/cgi-bin/fbidq.html?FBgn0051104) | - | [CG31104](http://flybase.org/cgi-bin/fbidq.html?FBgn0051104) |
| [FBgn0051122](http://flybase.org/cgi-bin/fbidq.html?FBgn0051122) | - | [CG31122](http://flybase.org/cgi-bin/fbidq.html?FBgn0051122) |
| [FBgn0051171](http://flybase.org/cgi-bin/fbidq.html?FBgn0051171) | - | [CG31171](http://flybase.org/cgi-bin/fbidq.html?FBgn0051171) |
| [FBgn0045470](http://flybase.org/cgi-bin/fbidq.html?FBgn0045470) | Gustatory receptor 93b | [Gr93b](http://flybase.org/cgi-bin/fbidq.html?FBgn0045470) |
| [FBgn0051451](http://flybase.org/cgi-bin/fbidq.html?FBgn0051451) | - | [CR31451](http://flybase.org/cgi-bin/fbidq.html?FBgn0051451) |
| [FBgn0027564](http://flybase.org/cgi-bin/fbidq.html?FBgn0027564) | - | [CG3149](http://flybase.org/cgi-bin/fbidq.html?FBgn0027564) |
| [FBgn0051690](http://flybase.org/cgi-bin/fbidq.html?FBgn0051690) | - | [CG31690](http://flybase.org/cgi-bin/fbidq.html?FBgn0051690) |
| [FBgn0051700](http://flybase.org/cgi-bin/fbidq.html?FBgn0051700) | - | [CR31700](http://flybase.org/cgi-bin/fbidq.html?FBgn0051700) |
| [FBgn0043841](http://flybase.org/cgi-bin/fbidq.html?FBgn0043841) | virus-induced RNA 1 | [vir-1](http://flybase.org/cgi-bin/fbidq.html?FBgn0043841) |
| [FBgn0051851](http://flybase.org/cgi-bin/fbidq.html?FBgn0051851) | - | [CG31851](http://flybase.org/cgi-bin/fbidq.html?FBgn0051851) |
| [FBgn0052000](http://flybase.org/cgi-bin/fbidq.html?FBgn0052000) | - | [CG32000](http://flybase.org/cgi-bin/fbidq.html?FBgn0052000) |
| [FBgn0011642](http://flybase.org/cgi-bin/fbidq.html?FBgn0011642) | Zyxin | [Zyx](http://flybase.org/cgi-bin/fbidq.html?FBgn0011642) |
| [FBgn0001228](http://flybase.org/cgi-bin/fbidq.html?FBgn0001228) | Heat shock gene 67Bb | [Hsp67Bb](http://flybase.org/cgi-bin/fbidq.html?FBgn0001228) |
| [FBgn0264489](http://flybase.org/cgi-bin/fbidq.html?FBgn0264489) | - | [CG43897](http://flybase.org/cgi-bin/fbidq.html?FBgn0264489) |
| [FBgn0052114](http://flybase.org/cgi-bin/fbidq.html?FBgn0052114) | - | [CG32114](http://flybase.org/cgi-bin/fbidq.html?FBgn0052114) |
| [FBgn0052138](http://flybase.org/cgi-bin/fbidq.html?FBgn0052138) | - | [CG32138](http://flybase.org/cgi-bin/fbidq.html?FBgn0052138) |
| [FBgn0052267](http://flybase.org/cgi-bin/fbidq.html?FBgn0052267) | - | [CG32267](http://flybase.org/cgi-bin/fbidq.html?FBgn0052267) |
| [FBgn0052277](http://flybase.org/cgi-bin/fbidq.html?FBgn0052277) | - | [CG32277](http://flybase.org/cgi-bin/fbidq.html?FBgn0052277) |
| [FBgn0052278](http://flybase.org/cgi-bin/fbidq.html?FBgn0052278) | - | [CG32278](http://flybase.org/cgi-bin/fbidq.html?FBgn0052278) |
| [FBgn0052365](http://flybase.org/cgi-bin/fbidq.html?FBgn0052365) | - | [CG32365](http://flybase.org/cgi-bin/fbidq.html?FBgn0052365) |
| [FBgn0004892](http://flybase.org/cgi-bin/fbidq.html?FBgn0004892) | sister of odd and bowl | [sob](http://flybase.org/cgi-bin/fbidq.html?FBgn0004892) |
| [FBgn0052447](http://flybase.org/cgi-bin/fbidq.html?FBgn0052447) | - | [CG32447](http://flybase.org/cgi-bin/fbidq.html?FBgn0052447) |
| [FBgn0026375](http://flybase.org/cgi-bin/fbidq.html?FBgn0026375) | Rho GTPase activating protein p190 | [RhoGAPp190](http://flybase.org/cgi-bin/fbidq.html?FBgn0026375) |
| [FBgn0027066](http://flybase.org/cgi-bin/fbidq.html?FBgn0027066) | Eb1 | [Eb1](http://flybase.org/cgi-bin/fbidq.html?FBgn0027066) |
| [FBgn0052677](http://flybase.org/cgi-bin/fbidq.html?FBgn0052677) | X11Lbeta | [X11Lbeta](http://flybase.org/cgi-bin/fbidq.html?FBgn0052677) |
| [FBgn0052700](http://flybase.org/cgi-bin/fbidq.html?FBgn0052700) | - | [CG32700](http://flybase.org/cgi-bin/fbidq.html?FBgn0052700) |
| [FBgn0260458](http://flybase.org/cgi-bin/fbidq.html?FBgn0260458) | Peptidoglycan recognition protein LD | [PGRP-LD](http://flybase.org/cgi-bin/fbidq.html?FBgn0260458) |
| [FBgn0041224](http://flybase.org/cgi-bin/fbidq.html?FBgn0041224) | Gustatory receptor 97a | [Gr97a](http://flybase.org/cgi-bin/fbidq.html?FBgn0041224) |
| [FBgn0053096](http://flybase.org/cgi-bin/fbidq.html?FBgn0053096) | - | [CG33096](http://flybase.org/cgi-bin/fbidq.html?FBgn0053096) |
| [FBgn0014024](http://flybase.org/cgi-bin/fbidq.html?FBgn0014024) | RNA-binding protein 4F | [Rnp4F](http://flybase.org/cgi-bin/fbidq.html?FBgn0014024) |
| [FBgn0053123](http://flybase.org/cgi-bin/fbidq.html?FBgn0053123) | - | [CG33123](http://flybase.org/cgi-bin/fbidq.html?FBgn0053123) |
| [FBgn0002528](http://flybase.org/cgi-bin/fbidq.html?FBgn0002528) | Laminin B2 | [LanB2](http://flybase.org/cgi-bin/fbidq.html?FBgn0002528) |
| [FBgn0053255](http://flybase.org/cgi-bin/fbidq.html?FBgn0053255) | - | [CG33255](http://flybase.org/cgi-bin/fbidq.html?FBgn0053255) |
| [FBgn0031417](http://flybase.org/cgi-bin/fbidq.html?FBgn0031417) | - | [CG3597](http://flybase.org/cgi-bin/fbidq.html?FBgn0031417) |
| [FBgn0039838](http://flybase.org/cgi-bin/fbidq.html?FBgn0039838) | - | [CG3669](http://flybase.org/cgi-bin/fbidq.html?FBgn0039838) |
| [FBgn0031589](http://flybase.org/cgi-bin/fbidq.html?FBgn0031589) | - | [CG3714](http://flybase.org/cgi-bin/fbidq.html?FBgn0031589) |
| [FBgn0029853](http://flybase.org/cgi-bin/fbidq.html?FBgn0029853) | - | [CG3781](http://flybase.org/cgi-bin/fbidq.html?FBgn0029853) |
| [FBgn0063485](http://flybase.org/cgi-bin/fbidq.html?FBgn0063485) | Lasp | [Lasp](http://flybase.org/cgi-bin/fbidq.html?FBgn0063485) |
| [FBgn0010382](http://flybase.org/cgi-bin/fbidq.html?FBgn0010382) | Cyclin E | [CycE](http://flybase.org/cgi-bin/fbidq.html?FBgn0010382) |
| [FBgn0030418](http://flybase.org/cgi-bin/fbidq.html?FBgn0030418) | - | [CG4004](http://flybase.org/cgi-bin/fbidq.html?FBgn0030418) |
| [FBgn0058057](http://flybase.org/cgi-bin/fbidq.html?FBgn0058057) | - | [CG40057](http://flybase.org/cgi-bin/fbidq.html?FBgn0058057) |
| [FBgn0058198](http://flybase.org/cgi-bin/fbidq.html?FBgn0058198) | - | [CG40198](http://flybase.org/cgi-bin/fbidq.html?FBgn0058198) |
| [FBgn0058221](http://flybase.org/cgi-bin/fbidq.html?FBgn0058221) | - | [CG40221](http://flybase.org/cgi-bin/fbidq.html?FBgn0058221) |
| [FBgn0084049](http://flybase.org/cgi-bin/fbidq.html?FBgn0084049) | - | [CR41440](http://flybase.org/cgi-bin/fbidq.html?FBgn0084049) |
| [FBgn0250816](http://flybase.org/cgi-bin/fbidq.html?FBgn0250816) | Argonaute 3 | [AGO3](http://flybase.org/cgi-bin/fbidq.html?FBgn0250816) |
| [FBgn0058354](http://flybase.org/cgi-bin/fbidq.html?FBgn0058354) | - | [CR40354](http://flybase.org/cgi-bin/fbidq.html?FBgn0058354) |
| FBgn0011288 | Synaptosomal associated protein 25kDa | Snap25 |
| [FBgn0011769](http://flybase.org/cgi-bin/fbidq.html?FBgn0011769) | Ferredoxin | [Fdxh](http://flybase.org/cgi-bin/fbidq.html?FBgn0011769) |
| [FBgn0010288](http://flybase.org/cgi-bin/fbidq.html?FBgn0010288) | Ubiquitin carboxy-terminal hydrolase | [Uch](http://flybase.org/cgi-bin/fbidq.html?FBgn0010288) |
| [FBgn0038795](http://flybase.org/cgi-bin/fbidq.html?FBgn0038795) | - | [CG4335](http://flybase.org/cgi-bin/fbidq.html?FBgn0038795) |
| [FBgn0086711](http://flybase.org/cgi-bin/fbidq.html?FBgn0086711) | moladietz | [mol](http://flybase.org/cgi-bin/fbidq.html?FBgn0086711) |
| [FBgn0035970](http://flybase.org/cgi-bin/fbidq.html?FBgn0035970) | - | [CG4483](http://flybase.org/cgi-bin/fbidq.html?FBgn0035970) |
| [FBgn0031895](http://flybase.org/cgi-bin/fbidq.html?FBgn0031895) | - | [CG4497](http://flybase.org/cgi-bin/fbidq.html?FBgn0031895) |
| [FBgn0011584](http://flybase.org/cgi-bin/fbidq.html?FBgn0011584) | Translocation protein 1 | [Trp1](http://flybase.org/cgi-bin/fbidq.html?FBgn0011584) |
| [FBgn0036614](http://flybase.org/cgi-bin/fbidq.html?FBgn0036614) | - | [CG4925](http://flybase.org/cgi-bin/fbidq.html?FBgn0036614) |
| [FBgn0021750](http://flybase.org/cgi-bin/fbidq.html?FBgn0021750) | Seryl-tRNA synthetase | [Aats-ser](http://flybase.org/cgi-bin/fbidq.html?FBgn0021750) |
| [FBgn0083975](http://flybase.org/cgi-bin/fbidq.html?FBgn0083975) | Neuroligin 4 | [Nlg4](http://flybase.org/cgi-bin/fbidq.html?FBgn0083975) |
| [FBgn0036581](http://flybase.org/cgi-bin/fbidq.html?FBgn0036581) | Mediator complex subunit 10 | [MED10](http://flybase.org/cgi-bin/fbidq.html?FBgn0036581) |
| [FBgn0038331](http://flybase.org/cgi-bin/fbidq.html?FBgn0038331) | Cerebral cavernous malformation 3 ortholog | [Ccm3](http://flybase.org/cgi-bin/fbidq.html?FBgn0038331) |
| [FBgn0266724](http://flybase.org/cgi-bin/fbidq.html?FBgn0266724) | TRAPP subunit 20 ortholog (S. cerevisiae) | [Trs20](http://flybase.org/cgi-bin/fbidq.html?FBgn0266724) |
| [FBgn0038474](http://flybase.org/cgi-bin/fbidq.html?FBgn0038474) | mitochondrial ribosomal protein S11 | [mRpS11](http://flybase.org/cgi-bin/fbidq.html?FBgn0038474) |
| [FBgn0034354](http://flybase.org/cgi-bin/fbidq.html?FBgn0034354) | Glutathione S transferase E11 | [GstE11](http://flybase.org/cgi-bin/fbidq.html?FBgn0034354) |
| [FBgn0037892](http://flybase.org/cgi-bin/fbidq.html?FBgn0037892) | mitochondrial ribosomal protein L40 | [mRpL40](http://flybase.org/cgi-bin/fbidq.html?FBgn0037892) |
| [FBgn0038485](http://flybase.org/cgi-bin/fbidq.html?FBgn0038485) | - | [CG5255](http://flybase.org/cgi-bin/fbidq.html?FBgn0038485) |
| [FBgn0262468](http://flybase.org/cgi-bin/fbidq.html?FBgn0262468) | vibrator | [vib](http://flybase.org/cgi-bin/fbidq.html?FBgn0262468) |
| [FBgn0032243](http://flybase.org/cgi-bin/fbidq.html?FBgn0032243) | Kinesin-like protein at 31E | [Klp31E](http://flybase.org/cgi-bin/fbidq.html?FBgn0032243) |
| [FBgn0015268](http://flybase.org/cgi-bin/fbidq.html?FBgn0015268) | Nucleosome assembly protein 1 | [Nap1](http://flybase.org/cgi-bin/fbidq.html?FBgn0015268) |
| [FBgn0038977](http://flybase.org/cgi-bin/fbidq.html?FBgn0038977) | - | [CG5376](http://flybase.org/cgi-bin/fbidq.html?FBgn0038977) |
| [FBgn0032215](http://flybase.org/cgi-bin/fbidq.html?FBgn0032215) | - | [CG5385](http://flybase.org/cgi-bin/fbidq.html?FBgn0032215) |
| [FBgn0005674](http://flybase.org/cgi-bin/fbidq.html?FBgn0005674) | Glutamyl-prolyl-tRNA synthetase | [Aats-glupro](http://flybase.org/cgi-bin/fbidq.html?FBgn0005674) |
| [FBgn0022959](http://flybase.org/cgi-bin/fbidq.html?FBgn0022959) | ypsilon schachtel | [yps](http://flybase.org/cgi-bin/fbidq.html?FBgn0022959) |
| [FBgn0032200](http://flybase.org/cgi-bin/fbidq.html?FBgn0032200) | - | [CG5676](http://flybase.org/cgi-bin/fbidq.html?FBgn0032200) |
| [FBgn0004449](http://flybase.org/cgi-bin/fbidq.html?FBgn0004449) | Tenascin major | [Ten-m](http://flybase.org/cgi-bin/fbidq.html?FBgn0004449) |
| [FBgn0032193](http://flybase.org/cgi-bin/fbidq.html?FBgn0032193) | - | [CG5727](http://flybase.org/cgi-bin/fbidq.html?FBgn0032193) |
| [FBgn0000083](http://flybase.org/cgi-bin/fbidq.html?FBgn0000083) | Annexin B9 | [AnxB9](http://flybase.org/cgi-bin/fbidq.html?FBgn0000083) |
| [FBgn0028894](http://flybase.org/cgi-bin/fbidq.html?FBgn0028894) | - | [CG5869](http://flybase.org/cgi-bin/fbidq.html?FBgn0028894) |
| [FBgn0036557](http://flybase.org/cgi-bin/fbidq.html?FBgn0036557) | mitochondrial ribosomal protein S31 | [mRpS31](http://flybase.org/cgi-bin/fbidq.html?FBgn0036557) |
| [FBgn0015795](http://flybase.org/cgi-bin/fbidq.html?FBgn0015795) | Rab7 | [Rab7](http://flybase.org/cgi-bin/fbidq.html?FBgn0015795) |
| [FBgn0032593](http://flybase.org/cgi-bin/fbidq.html?FBgn0032593) | Transient receptor potential cation channel gamma | [Trpgamma](http://flybase.org/cgi-bin/fbidq.html?FBgn0032593) |
| [FBgn0000146](http://flybase.org/cgi-bin/fbidq.html?FBgn0000146) | aubergine | [aub](http://flybase.org/cgi-bin/fbidq.html?FBgn0000146) |
| [FBgn0004401](http://flybase.org/cgi-bin/fbidq.html?FBgn0004401) | Protein on ecdysone puffs | [Pep](http://flybase.org/cgi-bin/fbidq.html?FBgn0004401) |
| [FBgn0032256](http://flybase.org/cgi-bin/fbidq.html?FBgn0032256) | RluA-2 | [RluA-2](http://flybase.org/cgi-bin/fbidq.html?FBgn0032256) |
| [FBgn0032456](http://flybase.org/cgi-bin/fbidq.html?FBgn0032456) | Multidrug-Resistance like Protein 1 | [MRP](http://flybase.org/cgi-bin/fbidq.html?FBgn0032456) |
| [FBgn0038316](http://flybase.org/cgi-bin/fbidq.html?FBgn0038316) | - | [CG6276](http://flybase.org/cgi-bin/fbidq.html?FBgn0038316) |
| [FBgn0032646](http://flybase.org/cgi-bin/fbidq.html?FBgn0032646) | - | [CG6412](http://flybase.org/cgi-bin/fbidq.html?FBgn0032646) |
| [FBgn0034259](http://flybase.org/cgi-bin/fbidq.html?FBgn0034259) | - | [P32](http://flybase.org/cgi-bin/fbidq.html?FBgn0034259) |
| [FBgn0034166](http://flybase.org/cgi-bin/fbidq.html?FBgn0034166) | - | [CG6472](http://flybase.org/cgi-bin/fbidq.html?FBgn0034166) |
| [FBgn0034247](http://flybase.org/cgi-bin/fbidq.html?FBgn0034247) | - | [CG6484](http://flybase.org/cgi-bin/fbidq.html?FBgn0034247) |
| [FBgn0032422](http://flybase.org/cgi-bin/fbidq.html?FBgn0032422) | atilla | [atilla](http://flybase.org/cgi-bin/fbidq.html?FBgn0032422) |
| [FBgn0036063](http://flybase.org/cgi-bin/fbidq.html?FBgn0036063) | - | [CG6674](http://flybase.org/cgi-bin/fbidq.html?FBgn0036063) |
| [FBgn0264087](http://flybase.org/cgi-bin/fbidq.html?FBgn0264087) | Slowpoke binding protein | [Slob](http://flybase.org/cgi-bin/fbidq.html?FBgn0264087) |
| [FBgn0039238](http://flybase.org/cgi-bin/fbidq.html?FBgn0039238) | - | [CG7016](http://flybase.org/cgi-bin/fbidq.html?FBgn0039238) |
| [FBgn0037130](http://flybase.org/cgi-bin/fbidq.html?FBgn0037130) | Syntrophin-like 1 | [Syn1](http://flybase.org/cgi-bin/fbidq.html?FBgn0037130) |
| [FBgn0035871](http://flybase.org/cgi-bin/fbidq.html?FBgn0035871) | Bax Inhibitor-1 | [BI-1](http://flybase.org/cgi-bin/fbidq.html?FBgn0035871) |
| [FBgn0035870](http://flybase.org/cgi-bin/fbidq.html?FBgn0035870) | Gustatory receptor 66a | [Gr66a](http://flybase.org/cgi-bin/fbidq.html?FBgn0035870) |
| [FBgn0031940](http://flybase.org/cgi-bin/fbidq.html?FBgn0031940) | - | [CG7214](http://flybase.org/cgi-bin/fbidq.html?FBgn0031940) |
| [FBgn0038569](http://flybase.org/cgi-bin/fbidq.html?FBgn0038569) | - | [CG7218](http://flybase.org/cgi-bin/fbidq.html?FBgn0038569) |
| [FBgn0031968](http://flybase.org/cgi-bin/fbidq.html?FBgn0031968) | - | [CG7231](http://flybase.org/cgi-bin/fbidq.html?FBgn0031968) |
| [FBgn0035689](http://flybase.org/cgi-bin/fbidq.html?FBgn0035689) | - | [CG7376](http://flybase.org/cgi-bin/fbidq.html?FBgn0035689) |
| [FBgn0038098](http://flybase.org/cgi-bin/fbidq.html?FBgn0038098) | - | [CG7381](http://flybase.org/cgi-bin/fbidq.html?FBgn0038098) |
| [FBgn0038533](http://flybase.org/cgi-bin/fbidq.html?FBgn0038533) | - | [CG7523](http://flybase.org/cgi-bin/fbidq.html?FBgn0038533) |
| [FBgn0036153](http://flybase.org/cgi-bin/fbidq.html?FBgn0036153) | - | [CG7573](http://flybase.org/cgi-bin/fbidq.html?FBgn0036153) |
| [FBgn0039681](http://flybase.org/cgi-bin/fbidq.html?FBgn0039681) | - | [CG7582](http://flybase.org/cgi-bin/fbidq.html?FBgn0039681) |
| [FBgn0039687](http://flybase.org/cgi-bin/fbidq.html?FBgn0039687) | - | [CG7593](http://flybase.org/cgi-bin/fbidq.html?FBgn0039687) |
| [FBgn0037093](http://flybase.org/cgi-bin/fbidq.html?FBgn0037093) | - | [Cdk12](http://flybase.org/cgi-bin/fbidq.html?FBgn0037093) |
| [FBgn0036145](http://flybase.org/cgi-bin/fbidq.html?FBgn0036145) | - | [CG7607](http://flybase.org/cgi-bin/fbidq.html?FBgn0036145) |
| [FBgn0030993](http://flybase.org/cgi-bin/fbidq.html?FBgn0030993) | Mec2 | [Mec2](http://flybase.org/cgi-bin/fbidq.html?FBgn0030993) |
| [FBgn0036133](http://flybase.org/cgi-bin/fbidq.html?FBgn0036133) | - | [CG7638](http://flybase.org/cgi-bin/fbidq.html?FBgn0036133) |
| [FBgn0038645](http://flybase.org/cgi-bin/fbidq.html?FBgn0038645) | - | [CG7714](http://flybase.org/cgi-bin/fbidq.html?FBgn0038645) |
| [FBgn0036496](http://flybase.org/cgi-bin/fbidq.html?FBgn0036496) | - | [CG7804](http://flybase.org/cgi-bin/fbidq.html?FBgn0036496) |
| [FBgn0025808](http://flybase.org/cgi-bin/fbidq.html?FBgn0025808) | Rad17 | [Rad17](http://flybase.org/cgi-bin/fbidq.html?FBgn0025808) |
| [FBgn0031003](http://flybase.org/cgi-bin/fbidq.html?FBgn0031003) | - | [CG7889](http://flybase.org/cgi-bin/fbidq.html?FBgn0031003) |
| [FBgn0039736](http://flybase.org/cgi-bin/fbidq.html?FBgn0039736) | - | [CG7912](http://flybase.org/cgi-bin/fbidq.html?FBgn0039736) |
| [FBgn0037546](http://flybase.org/cgi-bin/fbidq.html?FBgn0037546) | muscarinic Acetylcholine Receptor, B-type | [mAChR-B](http://flybase.org/cgi-bin/fbidq.html?FBgn0037546) |
| [FBgn0004228](http://flybase.org/cgi-bin/fbidq.html?FBgn0004228) | midgut expression 1 | [mex1](http://flybase.org/cgi-bin/fbidq.html?FBgn0004228) |
| [FBgn0002626](http://flybase.org/cgi-bin/fbidq.html?FBgn0002626) | Ribosomal protein L32 | [RpL32](http://flybase.org/cgi-bin/fbidq.html?FBgn0002626) |
| [FBgn0003502](http://flybase.org/cgi-bin/fbidq.html?FBgn0003502) | Btk family kinase at 29A | [Btk29A](http://flybase.org/cgi-bin/fbidq.html?FBgn0003502) |
| [FBgn0000568](http://flybase.org/cgi-bin/fbidq.html?FBgn0000568) | Ecdysone-induced protein 75B | [Eip75B](http://flybase.org/cgi-bin/fbidq.html?FBgn0000568) |
| [FBgn0034011](http://flybase.org/cgi-bin/fbidq.html?FBgn0034011) | - | [CG8160](http://flybase.org/cgi-bin/fbidq.html?FBgn0034011) |
| [FBgn0014865](http://flybase.org/cgi-bin/fbidq.html?FBgn0014865) | Metchnikowin | [Mtk](http://flybase.org/cgi-bin/fbidq.html?FBgn0014865) |
| [FBgn0037702](http://flybase.org/cgi-bin/fbidq.html?FBgn0037702) | - | [CG8176](http://flybase.org/cgi-bin/fbidq.html?FBgn0037702) |
| [FBgn0034032](http://flybase.org/cgi-bin/fbidq.html?FBgn0034032) | - | [CG8195](http://flybase.org/cgi-bin/fbidq.html?FBgn0034032) |
| [FBgn0262512](http://flybase.org/cgi-bin/fbidq.html?FBgn0262512) | Vacuolar H[+] ATPase 14kD subunit 1 | [Vha14-1](http://flybase.org/cgi-bin/fbidq.html?FBgn0262512) |
| [FBgn0033357](http://flybase.org/cgi-bin/fbidq.html?FBgn0033357) | Translocase of outer membrane 7 | [Tom7](http://flybase.org/cgi-bin/fbidq.html?FBgn0033357) |
| [FBgn0004921](http://flybase.org/cgi-bin/fbidq.html?FBgn0004921) | G protein gamma 1 | [Ggamma1](http://flybase.org/cgi-bin/fbidq.html?FBgn0004921) |
| [FBgn0015321](http://flybase.org/cgi-bin/fbidq.html?FBgn0015321) | Ubiquitin conjugating enzyme 4 | [UbcD4](http://flybase.org/cgi-bin/fbidq.html?FBgn0015321) |
| [FBgn0010348](http://flybase.org/cgi-bin/fbidq.html?FBgn0010348) | ADP ribosylation factor at 79F | [Arf79F](http://flybase.org/cgi-bin/fbidq.html?FBgn0010348) |
| [FBgn0024542](http://flybase.org/cgi-bin/fbidq.html?FBgn0024542) | Neosin | [Neos](http://flybase.org/cgi-bin/fbidq.html?FBgn0024542) |
| [FBgn0033287](http://flybase.org/cgi-bin/fbidq.html?FBgn0033287) | - | [CG8701](http://flybase.org/cgi-bin/fbidq.html?FBgn0033287) |
| [FBgn0033690](http://flybase.org/cgi-bin/fbidq.html?FBgn0033690) | Endonuclease G | [EndoG](http://flybase.org/cgi-bin/fbidq.html?FBgn0033690) |
| [FBgn0000723](http://flybase.org/cgi-bin/fbidq.html?FBgn0000723) | Fps oncogene analog | [Fps85D](http://flybase.org/cgi-bin/fbidq.html?FBgn0000723) |
| [FBgn0033688](http://flybase.org/cgi-bin/fbidq.html?FBgn0033688) | pre-mRNA processing factor 8 | [Prp8](http://flybase.org/cgi-bin/fbidq.html?FBgn0033688) |
| [FBgn0001092](http://flybase.org/cgi-bin/fbidq.html?FBgn0001092) | Glyceraldehyde 3 phosphate dehydrogenase 2 | [Gapdh2](http://flybase.org/cgi-bin/fbidq.html?FBgn0001092) |
| [FBgn0027529](http://flybase.org/cgi-bin/fbidq.html?FBgn0027529) | - | [CG8920](http://flybase.org/cgi-bin/fbidq.html?FBgn0027529) |
| [FBgn0001216](http://flybase.org/cgi-bin/fbidq.html?FBgn0001216) | Heat shock protein cognate 1 | [Hsc70-1](http://flybase.org/cgi-bin/fbidq.html?FBgn0001216) |
| [FBgn0044020](http://flybase.org/cgi-bin/fbidq.html?FBgn0044020) | Roc2 | [Roc2](http://flybase.org/cgi-bin/fbidq.html?FBgn0044020) |
| [FBgn0030614](http://flybase.org/cgi-bin/fbidq.html?FBgn0030614) | - | [CG9072](http://flybase.org/cgi-bin/fbidq.html?FBgn0030614) |
| [FBgn0001941](http://flybase.org/cgi-bin/fbidq.html?FBgn0001941) | infertile crescent | [ifc](http://flybase.org/cgi-bin/fbidq.html?FBgn0001941) |
| [FBgn0034666](http://flybase.org/cgi-bin/fbidq.html?FBgn0034666) | - | [CG9294](http://flybase.org/cgi-bin/fbidq.html?FBgn0034666) |
| [FBgn0032881](http://flybase.org/cgi-bin/fbidq.html?FBgn0032881) | - | [CG9319](http://flybase.org/cgi-bin/fbidq.html?FBgn0032881) |
| [FBgn0037686](http://flybase.org/cgi-bin/fbidq.html?FBgn0037686) | Ribosomal protein L34b | [RpL34b](http://flybase.org/cgi-bin/fbidq.html?FBgn0037686) |
| [FBgn0002868](http://flybase.org/cgi-bin/fbidq.html?FBgn0002868) | Metallothionein A | [MtnA](http://flybase.org/cgi-bin/fbidq.html?FBgn0002868) |
| [FBgn0013746](http://flybase.org/cgi-bin/fbidq.html?FBgn0013746) | alien | [alien](http://flybase.org/cgi-bin/fbidq.html?FBgn0013746) |
| [FBgn0030777](http://flybase.org/cgi-bin/fbidq.html?FBgn0030777) | - | [CG9672](http://flybase.org/cgi-bin/fbidq.html?FBgn0030777) |
| [FBgn0030775](http://flybase.org/cgi-bin/fbidq.html?FBgn0030775) | - | [CG9673](http://flybase.org/cgi-bin/fbidq.html?FBgn0030775) |
| [FBgn0004108](http://flybase.org/cgi-bin/fbidq.html?FBgn0004108) | Neurotactin | [Nrt](http://flybase.org/cgi-bin/fbidq.html?FBgn0004108) |
| [FBgn0000412](http://flybase.org/cgi-bin/fbidq.html?FBgn0000412) | D1 chromosomal protein | [D1](http://flybase.org/cgi-bin/fbidq.html?FBgn0000412) |
| [FBgn0260935](http://flybase.org/cgi-bin/fbidq.html?FBgn0260935) | immune response deficient 1 | [ird1](http://flybase.org/cgi-bin/fbidq.html?FBgn0260935) |
| [FBgn0264672](http://flybase.org/cgi-bin/fbidq.html?FBgn0264672) | EGF-domain O-GlcNAc transferase | [Eogt](http://flybase.org/cgi-bin/fbidq.html?FBgn0264672) |
| [FBgn0034817](http://flybase.org/cgi-bin/fbidq.html?FBgn0034817) | Arginine methyltransferase 7 | [Art7](http://flybase.org/cgi-bin/fbidq.html?FBgn0034817) |
| [FBgn0019660](http://flybase.org/cgi-bin/fbidq.html?FBgn0019660) | RNA on the X 2 | [roX2](http://flybase.org/cgi-bin/fbidq.html?FBgn0019660) |
| [FBgn0041717](http://flybase.org/cgi-bin/fbidq.html?FBgn0041717) | U6atac snRNA at 29B | [snRNA:U6atac:29B](http://flybase.org/cgi-bin/fbidq.html?FBgn0041717) |
| - | 18SrRNA | CR40456 |
| - | 28SrRNA | CR40459 |
