# Supplementary material for: Transcriptional Regionalization of the Fruit Fly’s Airway Epithelium
Source: PLoS One. 2014 Jul 14;9(7):e102534. doi: 10.1371/journal.pone.0102534 (PMC4097054; doi:10.1371/journal.pone.0102534)
Supplement: Table S2 — Genes with 1.5 fold higher expression in primary/secondary/terminal branches. (DOCX) [file pone.0102534.s003.docx]

**Table S2:**

**Genes with 1.5 fold higher expression in primary/secondary/terminal branches (in 2 out of three independent experiments)**

| **FBID KEY** | **NAME** | **SYMBOL** |
| --- | --- | --- |
| [FBgn0037498](http://flybase.org/cgi-bin/fbidq.html?FBgn0037498) | - | [CG10029](http://flybase.org/cgi-bin/fbidq.html?FBgn0037498) |
| [FBgn0037493](http://flybase.org/cgi-bin/fbidq.html?FBgn0037493) | - | [CG10032](http://flybase.org/cgi-bin/fbidq.html?FBgn0037493) |
| [FBgn0037473](http://flybase.org/cgi-bin/fbidq.html?FBgn0037473) | - | [CG10068](http://flybase.org/cgi-bin/fbidq.html?FBgn0037473) |
| [FBgn0003475](http://flybase.org/cgi-bin/fbidq.html?FBgn0003475) | spire | [spir](http://flybase.org/cgi-bin/fbidq.html?FBgn0003475) |
| [FBgn0035720](http://flybase.org/cgi-bin/fbidq.html?FBgn0035720) | - | [CG10077](http://flybase.org/cgi-bin/fbidq.html?FBgn0035720) |
| FBgn0029506 | Tetraspanin 42Ee | Tsp42Ee |
| [FBgn0016684](http://flybase.org/cgi-bin/fbidq.html?FBgn0016684) | Na[+]-dependent inorganic phosphate cotransporter | [NaPi-T](http://flybase.org/cgi-bin/fbidq.html?FBgn0016684) |
| [FBgn0039112](http://flybase.org/cgi-bin/fbidq.html?FBgn0039112) | - | [CG10219](http://flybase.org/cgi-bin/fbidq.html?FBgn0039112) |
| [FBgn0039280](http://flybase.org/cgi-bin/fbidq.html?FBgn0039280) | Molybdenum cofactor synthesis 2 | [Mocs2](http://flybase.org/cgi-bin/fbidq.html?FBgn0039280) |
| [FBgn0261597](http://flybase.org/cgi-bin/fbidq.html?FBgn0261597) | Ribosomal protein S26 | [RpS26](http://flybase.org/cgi-bin/fbidq.html?FBgn0261597) |
| [FBgn0036271](http://flybase.org/cgi-bin/fbidq.html?FBgn0036271) | Porphobilinogen synthase | [Pbgs](http://flybase.org/cgi-bin/fbidq.html?FBgn0036271) |
| [FBgn0039116](http://flybase.org/cgi-bin/fbidq.html?FBgn0039116) | - | [CG10375](http://flybase.org/cgi-bin/fbidq.html?FBgn0039116) |
| [FBgn0032819](http://flybase.org/cgi-bin/fbidq.html?FBgn0032819) | - | [CG10463](http://flybase.org/cgi-bin/fbidq.html?FBgn0032819) |
| [FBgn0261396](http://flybase.org/cgi-bin/fbidq.html?FBgn0261396) | Regulatory particle non-ATPase 3 | [Rpn3](http://flybase.org/cgi-bin/fbidq.html?FBgn0261396) |
| [FBgn0036289](http://flybase.org/cgi-bin/fbidq.html?FBgn0036289) | - | [CG10657](http://flybase.org/cgi-bin/fbidq.html?FBgn0036289) |
| [FBgn0039147](http://flybase.org/cgi-bin/fbidq.html?FBgn0039147) | - | [CG10694](http://flybase.org/cgi-bin/fbidq.html?FBgn0039147) |
| [FBgn0030011](http://flybase.org/cgi-bin/fbidq.html?FBgn0030011) | Gbeta5 | [Gbeta5](http://flybase.org/cgi-bin/fbidq.html?FBgn0030011) |
| [FBgn0031865](http://flybase.org/cgi-bin/fbidq.html?FBgn0031865) | Na[+]/H[+] hydrogen antiporter 1 | [Nha1](http://flybase.org/cgi-bin/fbidq.html?FBgn0031865) |
| [FBgn0023213](http://flybase.org/cgi-bin/fbidq.html?FBgn0023213) | eukaryotic translation initiation factor 4G | [eIF4G](http://flybase.org/cgi-bin/fbidq.html?FBgn0023213) |
| [FBgn0033830](http://flybase.org/cgi-bin/fbidq.html?FBgn0033830) | - | [CG10814](http://flybase.org/cgi-bin/fbidq.html?FBgn0033830) |
| [FBgn0038845](http://flybase.org/cgi-bin/fbidq.html?FBgn0038845) | - | [CG10827](http://flybase.org/cgi-bin/fbidq.html?FBgn0038845) |
| [FBgn0034253](http://flybase.org/cgi-bin/fbidq.html?FBgn0034253) | - | [CG10936](http://flybase.org/cgi-bin/fbidq.html?FBgn0034253) |
| [FBgn0261592](http://flybase.org/cgi-bin/fbidq.html?FBgn0261592) | Ribosomal protein S6 | [RpS6](http://flybase.org/cgi-bin/fbidq.html?FBgn0261592) |
| [FBgn0000535](http://flybase.org/cgi-bin/fbidq.html?FBgn0000535) | ether a go-go | [eag](http://flybase.org/cgi-bin/fbidq.html?FBgn0000535) |
| [FBgn0053523](http://flybase.org/cgi-bin/fbidq.html?FBgn0053523) | VAMP-associated protein of 33kDa ortholog B | [Vap-33B](http://flybase.org/cgi-bin/fbidq.html?FBgn0053523) |
| [FBgn0084815](http://flybase.org/cgi-bin/fbidq.html?FBgn0084815) | - | [CG11091](http://flybase.org/cgi-bin/fbidq.html?FBgn0084815) |
| [FBgn0037205](http://flybase.org/cgi-bin/fbidq.html?FBgn0037205) | Brother of Yb | [BoYb](http://flybase.org/cgi-bin/fbidq.html?FBgn0037205) |
| [FBgn0034913](http://flybase.org/cgi-bin/fbidq.html?FBgn0034913) | Synaptosomal-associated protein 29kDa | [Snap29](http://flybase.org/cgi-bin/fbidq.html?FBgn0034913) |
| [FBgn0034442](http://flybase.org/cgi-bin/fbidq.html?FBgn0034442) | - | [CG11257](http://flybase.org/cgi-bin/fbidq.html?FBgn0034442) |
| [FBgn0040366](http://flybase.org/cgi-bin/fbidq.html?FBgn0040366) | - | [CG11398](http://flybase.org/cgi-bin/fbidq.html?FBgn0040366) |
| [FBgn0000826](http://flybase.org/cgi-bin/fbidq.html?FBgn0000826) | pan gu | [png](http://flybase.org/cgi-bin/fbidq.html?FBgn0000826) |
| [FBgn0000117](http://flybase.org/cgi-bin/fbidq.html?FBgn0000117) | armadillo | [arm](http://flybase.org/cgi-bin/fbidq.html?FBgn0000117) |
| [FBgn0027334](http://flybase.org/cgi-bin/fbidq.html?FBgn0027334) | lethal (1) G0004 | [l(1)G0004](http://flybase.org/cgi-bin/fbidq.html?FBgn0027334) |
| [FBgn0262617](http://flybase.org/cgi-bin/fbidq.html?FBgn0262617) | - | [CG43143](http://flybase.org/cgi-bin/fbidq.html?FBgn0262617) |
| [FBgn0037643](http://flybase.org/cgi-bin/fbidq.html?FBgn0037643) | skpA associated protein | [skap](http://flybase.org/cgi-bin/fbidq.html?FBgn0037643) |
| [FBgn0035421](http://flybase.org/cgi-bin/fbidq.html?FBgn0035421) | - | [CG12034](http://flybase.org/cgi-bin/fbidq.html?FBgn0035421) |
| [FBgn0014032](http://flybase.org/cgi-bin/fbidq.html?FBgn0014032) | Sepiapterin reductase | [Sptr](http://flybase.org/cgi-bin/fbidq.html?FBgn0014032) |
| [FBgn0030039](http://flybase.org/cgi-bin/fbidq.html?FBgn0030039) | - | [CG12123](http://flybase.org/cgi-bin/fbidq.html?FBgn0030039) |
| [FBgn0261260](http://flybase.org/cgi-bin/fbidq.html?FBgn0261260) | Megalin | [mgl](http://flybase.org/cgi-bin/fbidq.html?FBgn0261260) |
| [FBgn0037354](http://flybase.org/cgi-bin/fbidq.html?FBgn0037354) | - | [CG12171](http://flybase.org/cgi-bin/fbidq.html?FBgn0037354) |
| [FBgn0037970](http://flybase.org/cgi-bin/fbidq.html?FBgn0037970) | - | [CG12201](http://flybase.org/cgi-bin/fbidq.html?FBgn0037970) |
| [FBgn0038080](http://flybase.org/cgi-bin/fbidq.html?FBgn0038080) | - | [CG12279](http://flybase.org/cgi-bin/fbidq.html?FBgn0038080) |
| [FBgn0260635](http://flybase.org/cgi-bin/fbidq.html?FBgn0260635) | thread | [th](http://flybase.org/cgi-bin/fbidq.html?FBgn0260635) |
| [FBgn0001296](http://flybase.org/cgi-bin/fbidq.html?FBgn0001296) | karmoisin | [kar](http://flybase.org/cgi-bin/fbidq.html?FBgn0001296) |
| [FBgn0037370](http://flybase.org/cgi-bin/fbidq.html?FBgn0037370) | - | [CG1236](http://flybase.org/cgi-bin/fbidq.html?FBgn0037370) |
| [FBgn0039552](http://flybase.org/cgi-bin/fbidq.html?FBgn0039552) | - | [CG12426](http://flybase.org/cgi-bin/fbidq.html?FBgn0039552) |
| [FBgn0033770](http://flybase.org/cgi-bin/fbidq.html?FBgn0033770) | Wake-up-call | [wuc](http://flybase.org/cgi-bin/fbidq.html?FBgn0033770) |
| [FBgn0010380](http://flybase.org/cgi-bin/fbidq.html?FBgn0010380) | Adaptor Protein complex 1/2, beta subunit | [AP-1-2beta](http://flybase.org/cgi-bin/fbidq.html?FBgn0010380) |
| [FBgn0033301](http://flybase.org/cgi-bin/fbidq.html?FBgn0033301) | - | [CG12780](http://flybase.org/cgi-bin/fbidq.html?FBgn0033301) |
| [FBgn0028583](http://flybase.org/cgi-bin/fbidq.html?FBgn0028583) | la costa | [lcs](http://flybase.org/cgi-bin/fbidq.html?FBgn0028583) |
| [FBgn0037781](http://flybase.org/cgi-bin/fbidq.html?FBgn0037781) | Fancl | [Fancl](http://flybase.org/cgi-bin/fbidq.html?FBgn0037781) |
| [FBgn0033124](http://flybase.org/cgi-bin/fbidq.html?FBgn0033124) | Tetraspanin 42Ec | [Tsp42Ec](http://flybase.org/cgi-bin/fbidq.html?FBgn0033124) |
| [FBgn0040747](http://flybase.org/cgi-bin/fbidq.html?FBgn0040747) | - | [CG12853](http://flybase.org/cgi-bin/fbidq.html?FBgn0040747) |
| [FBgn0033512](http://flybase.org/cgi-bin/fbidq.html?FBgn0033512) | - | [CG12902](http://flybase.org/cgi-bin/fbidq.html?FBgn0033512) |
| [FBgn0032139](http://flybase.org/cgi-bin/fbidq.html?FBgn0032139) | - | [CG13116](http://flybase.org/cgi-bin/fbidq.html?FBgn0032139) |
| [FBgn0265001](http://flybase.org/cgi-bin/fbidq.html?FBgn0265001) | pickpocket 18 | [ppk18](http://flybase.org/cgi-bin/fbidq.html?FBgn0265001) |
| [FBgn0032175](http://flybase.org/cgi-bin/fbidq.html?FBgn0032175) | - | [CG13131](http://flybase.org/cgi-bin/fbidq.html?FBgn0032175) |
| [FBgn0032609](http://flybase.org/cgi-bin/fbidq.html?FBgn0032609) | - | [CG13280](http://flybase.org/cgi-bin/fbidq.html?FBgn0032609) |
| [FBgn0027094](http://flybase.org/cgi-bin/fbidq.html?FBgn0027094) | Alanyl-tRNA synthetase | [Aats-ala](http://flybase.org/cgi-bin/fbidq.html?FBgn0027094) |
| [FBgn0027490](http://flybase.org/cgi-bin/fbidq.html?FBgn0027490) | D12 | [D12](http://flybase.org/cgi-bin/fbidq.html?FBgn0027490) |
| [FBgn0037363](http://flybase.org/cgi-bin/fbidq.html?FBgn0037363) | - | [Atg17](http://flybase.org/cgi-bin/fbidq.html?FBgn0037363) |
| [FBgn0264740](http://flybase.org/cgi-bin/fbidq.html?FBgn0264740) | - | [CG43998](http://flybase.org/cgi-bin/fbidq.html?FBgn0264740) |
| [FBgn0039256](http://flybase.org/cgi-bin/fbidq.html?FBgn0039256) | - | [CG13647](http://flybase.org/cgi-bin/fbidq.html?FBgn0039256) |
| [FBgn0038961](http://flybase.org/cgi-bin/fbidq.html?FBgn0038961) | - | [CG13850](http://flybase.org/cgi-bin/fbidq.html?FBgn0038961) |
| [FBgn0263117](http://flybase.org/cgi-bin/fbidq.html?FBgn0263117) | - | [CG34377](http://flybase.org/cgi-bin/fbidq.html?FBgn0263117) |
| [FBgn0264707](http://flybase.org/cgi-bin/fbidq.html?FBgn0264707) | Rho guanine nucleotide exchange factor 3 | [RhoGEF3](http://flybase.org/cgi-bin/fbidq.html?FBgn0264707) |
| [FBgn0264272](http://flybase.org/cgi-bin/fbidq.html?FBgn0264272) | multiple wing hairs | [mwh](http://flybase.org/cgi-bin/fbidq.html?FBgn0264272) |
| [FBgn0039444](http://flybase.org/cgi-bin/fbidq.html?FBgn0039444) | TweedleD | [TwdlD](http://flybase.org/cgi-bin/fbidq.html?FBgn0039444) |
| [FBgn0004652](http://flybase.org/cgi-bin/fbidq.html?FBgn0004652) | fruitless | [fru](http://flybase.org/cgi-bin/fbidq.html?FBgn0004652) |
| [FBgn0031301](http://flybase.org/cgi-bin/fbidq.html?FBgn0031301) | - | [CG14339](http://flybase.org/cgi-bin/fbidq.html?FBgn0031301) |
| [FBgn0040353](http://flybase.org/cgi-bin/fbidq.html?FBgn0040353) | - | [CG14417](http://flybase.org/cgi-bin/fbidq.html?FBgn0040353) |
| [FBgn0039403](http://flybase.org/cgi-bin/fbidq.html?FBgn0039403) | Sld5 | [Sld5](http://flybase.org/cgi-bin/fbidq.html?FBgn0039403) |
| [FBgn0040645](http://flybase.org/cgi-bin/fbidq.html?FBgn0040645) | - | [CG14581](http://flybase.org/cgi-bin/fbidq.html?FBgn0040645) |
| [FBgn0037244](http://flybase.org/cgi-bin/fbidq.html?FBgn0037244) | - | [CG14647](http://flybase.org/cgi-bin/fbidq.html?FBgn0037244) |
| [FBgn0037275](http://flybase.org/cgi-bin/fbidq.html?FBgn0037275) | - | [CG14655](http://flybase.org/cgi-bin/fbidq.html?FBgn0037275) |
| [FBgn0003517](http://flybase.org/cgi-bin/fbidq.html?FBgn0003517) | stubarista | [sta](http://flybase.org/cgi-bin/fbidq.html?FBgn0003517) |
| [FBgn0053513](http://flybase.org/cgi-bin/fbidq.html?FBgn0053513) | NMDA receptor 2 | [Nmdar2](http://flybase.org/cgi-bin/fbidq.html?FBgn0053513) |
| [FBgn0053556](http://flybase.org/cgi-bin/fbidq.html?FBgn0053556) | formin 3 | [form3](http://flybase.org/cgi-bin/fbidq.html?FBgn0053556) |
| [FBgn0038217](http://flybase.org/cgi-bin/fbidq.html?FBgn0038217) | - | [CG14840](http://flybase.org/cgi-bin/fbidq.html?FBgn0038217) |
| FBgn0259244 | - | CG42342 |
| [FBgn0023416](http://flybase.org/cgi-bin/fbidq.html?FBgn0023416) | Ac3 | [Ac3](http://flybase.org/cgi-bin/fbidq.html?FBgn0023416) |
| [FBgn0010551](http://flybase.org/cgi-bin/fbidq.html?FBgn0010551) | lethal (2) 03709 | [l(2)03709](http://flybase.org/cgi-bin/fbidq.html?FBgn0010551) |
| [FBgn0023175](http://flybase.org/cgi-bin/fbidq.html?FBgn0023175) | Proteasome alpha7 subunit | [Prosalpha7](http://flybase.org/cgi-bin/fbidq.html?FBgn0023175) |
| [FBgn0266421](http://flybase.org/cgi-bin/fbidq.html?FBgn0266421) | Replication protein A3 | [RPA3](http://flybase.org/cgi-bin/fbidq.html?FBgn0266421) |
| [FBgn0028867](http://flybase.org/cgi-bin/fbidq.html?FBgn0028867) | - | [CR15280](http://flybase.org/cgi-bin/fbidq.html?FBgn0028867) |
| [FBgn0028855](http://flybase.org/cgi-bin/fbidq.html?FBgn0028855) | - | [CG15282](http://flybase.org/cgi-bin/fbidq.html?FBgn0028855) |
| [FBgn0028531](http://flybase.org/cgi-bin/fbidq.html?FBgn0028531) | - | [CG15286](http://flybase.org/cgi-bin/fbidq.html?FBgn0028531) |
| [FBgn0028526](http://flybase.org/cgi-bin/fbidq.html?FBgn0028526) | - | [CG15293](http://flybase.org/cgi-bin/fbidq.html?FBgn0028526) |
| [FBgn0030107](http://flybase.org/cgi-bin/fbidq.html?FBgn0030107) | - | [CG15370](http://flybase.org/cgi-bin/fbidq.html?FBgn0030107) |
| [FBgn0029694](http://flybase.org/cgi-bin/fbidq.html?FBgn0029694) | - | [CG15375](http://flybase.org/cgi-bin/fbidq.html?FBgn0029694) |
| [FBgn0001297](http://flybase.org/cgi-bin/fbidq.html?FBgn0001297) | kayak | [kay](http://flybase.org/cgi-bin/fbidq.html?FBgn0001297) |
| [FBgn0265045](http://flybase.org/cgi-bin/fbidq.html?FBgn0265045) | Stretchin-Mlck | [Strn-Mlck](http://flybase.org/cgi-bin/fbidq.html?FBgn0265045) |
| [FBgn0029993](http://flybase.org/cgi-bin/fbidq.html?FBgn0029993) | - | [CG1571](http://flybase.org/cgi-bin/fbidq.html?FBgn0029993) |
| [FBgn0265597](http://flybase.org/cgi-bin/fbidq.html?FBgn0265597) | radish | [rad](http://flybase.org/cgi-bin/fbidq.html?FBgn0265597) |
| [FBgn0029813](http://flybase.org/cgi-bin/fbidq.html?FBgn0029813) | - | [CG15766](http://flybase.org/cgi-bin/fbidq.html?FBgn0029813) |
| [FBgn0029864](http://flybase.org/cgi-bin/fbidq.html?FBgn0029864) | - | [CG15894](http://flybase.org/cgi-bin/fbidq.html?FBgn0029864) |
| [FBgn0029133](http://flybase.org/cgi-bin/fbidq.html?FBgn0029133) | REG | [REG](http://flybase.org/cgi-bin/fbidq.html?FBgn0029133) |
| [FBgn0030245](http://flybase.org/cgi-bin/fbidq.html?FBgn0030245) | - | [CG1637](http://flybase.org/cgi-bin/fbidq.html?FBgn0030245) |
| [FBgn0031110](http://flybase.org/cgi-bin/fbidq.html?FBgn0031110) | Odorant-binding protein 19b | [Obp19b](http://flybase.org/cgi-bin/fbidq.html?FBgn0031110) |
| [FBgn0031560](http://flybase.org/cgi-bin/fbidq.html?FBgn0031560) | - | [CG16713](http://flybase.org/cgi-bin/fbidq.html?FBgn0031560) |
| [FBgn0034459](http://flybase.org/cgi-bin/fbidq.html?FBgn0034459) | - | [CG16716](http://flybase.org/cgi-bin/fbidq.html?FBgn0034459) |
| [FBgn0034529](http://flybase.org/cgi-bin/fbidq.html?FBgn0034529) | - | [CG16742](http://flybase.org/cgi-bin/fbidq.html?FBgn0034529) |
| [FBgn0035348](http://flybase.org/cgi-bin/fbidq.html?FBgn0035348) | - | [CG16758](http://flybase.org/cgi-bin/fbidq.html?FBgn0035348) |
| [FBgn0032522](http://flybase.org/cgi-bin/fbidq.html?FBgn0032522) | - | [CG16848](http://flybase.org/cgi-bin/fbidq.html?FBgn0032522) |
| [FBgn0028544](http://flybase.org/cgi-bin/fbidq.html?FBgn0028544) | - | [CG16884](http://flybase.org/cgi-bin/fbidq.html?FBgn0028544) |
| [FBgn0262735](http://flybase.org/cgi-bin/fbidq.html?FBgn0262735) | IGF-II mRNA-binding protein | [Imp](http://flybase.org/cgi-bin/fbidq.html?FBgn0262735) |
| [FBgn0040291](http://flybase.org/cgi-bin/fbidq.html?FBgn0040291) | Roc1b | [Roc1b](http://flybase.org/cgi-bin/fbidq.html?FBgn0040291) |
| [FBgn0032281](http://flybase.org/cgi-bin/fbidq.html?FBgn0032281) | - | [CG17107](http://flybase.org/cgi-bin/fbidq.html?FBgn0032281) |
| [FBgn0032421](http://flybase.org/cgi-bin/fbidq.html?FBgn0032421) | crooked | [crok](http://flybase.org/cgi-bin/fbidq.html?FBgn0032421) |
| [FBgn0031168](http://flybase.org/cgi-bin/fbidq.html?FBgn0031168) | - | [CG1722](http://flybase.org/cgi-bin/fbidq.html?FBgn0031168) |
| [FBgn0034202](http://flybase.org/cgi-bin/fbidq.html?FBgn0034202) | - | [CG17287](http://flybase.org/cgi-bin/fbidq.html?FBgn0034202) |
| [FBgn0035640](http://flybase.org/cgi-bin/fbidq.html?FBgn0035640) | mad2 | [mad2](http://flybase.org/cgi-bin/fbidq.html?FBgn0035640) |
| [FBgn0031197](http://flybase.org/cgi-bin/fbidq.html?FBgn0031197) | - | [CG17601](http://flybase.org/cgi-bin/fbidq.html?FBgn0031197) |
| [FBgn0041004](http://flybase.org/cgi-bin/fbidq.html?FBgn0041004) | - | [CG17715](http://flybase.org/cgi-bin/fbidq.html?FBgn0041004) |
| [FBgn0265296](http://flybase.org/cgi-bin/fbidq.html?FBgn0265296) | Down syndrome cell adhesion molecule 2 | [Dscam2](http://flybase.org/cgi-bin/fbidq.html?FBgn0265296) |
| [FBgn0029647](http://flybase.org/cgi-bin/fbidq.html?FBgn0029647) | - | [CG17959](http://flybase.org/cgi-bin/fbidq.html?FBgn0029647) |
| [FBgn0034329](http://flybase.org/cgi-bin/fbidq.html?FBgn0034329) | Immune induced molecule 1 | [IM1](http://flybase.org/cgi-bin/fbidq.html?FBgn0034329) |
| [FBgn0034898](http://flybase.org/cgi-bin/fbidq.html?FBgn0034898) | - | [CG18128](http://flybase.org/cgi-bin/fbidq.html?FBgn0034898) |
| [FBgn0033431](http://flybase.org/cgi-bin/fbidq.html?FBgn0033431) | - | [CG1827](http://flybase.org/cgi-bin/fbidq.html?FBgn0033431) |
| [FBgn0002183](http://flybase.org/cgi-bin/fbidq.html?FBgn0002183) | dre4 | [dre4](http://flybase.org/cgi-bin/fbidq.html?FBgn0002183) |
| [FBgn0030330](http://flybase.org/cgi-bin/fbidq.html?FBgn0030330) | Transport and Golgi organization 10 | [Tango10](http://flybase.org/cgi-bin/fbidq.html?FBgn0030330) |
| [FBgn0033285](http://flybase.org/cgi-bin/fbidq.html?FBgn0033285) | - | [CG18449](http://flybase.org/cgi-bin/fbidq.html?FBgn0033285) |
| [FBgn0038348](http://flybase.org/cgi-bin/fbidq.html?FBgn0038348) | - | [CG18519](http://flybase.org/cgi-bin/fbidq.html?FBgn0038348) |
| [FBgn0042207](http://flybase.org/cgi-bin/fbidq.html?FBgn0042207) | - | [CG18530](http://flybase.org/cgi-bin/fbidq.html?FBgn0042207) |
| [FBgn0031470](http://flybase.org/cgi-bin/fbidq.html?FBgn0031470) | - | [CG18557](http://flybase.org/cgi-bin/fbidq.html?FBgn0031470) |
| [FBgn0036466](http://flybase.org/cgi-bin/fbidq.html?FBgn0036466) | - | [CG18581](http://flybase.org/cgi-bin/fbidq.html?FBgn0036466) |
| [FBgn0262103](http://flybase.org/cgi-bin/fbidq.html?FBgn0262103) | Salt-inducible kinase 3 | [Sik3](http://flybase.org/cgi-bin/fbidq.html?FBgn0262103) |
| [FBgn0040964](http://flybase.org/cgi-bin/fbidq.html?FBgn0040964) | - | [CG18661](http://flybase.org/cgi-bin/fbidq.html?FBgn0040964) |
| [FBgn0015509](http://flybase.org/cgi-bin/fbidq.html?FBgn0015509) | lin-19-like | [lin19](http://flybase.org/cgi-bin/fbidq.html?FBgn0015509) |
| [FBgn0039887](http://flybase.org/cgi-bin/fbidq.html?FBgn0039887) | - | [CG2053](http://flybase.org/cgi-bin/fbidq.html?FBgn0039887) |
| [FBgn0022702](http://flybase.org/cgi-bin/fbidq.html?FBgn0022702) | Chitinase 2 | [Cht2](http://flybase.org/cgi-bin/fbidq.html?FBgn0022702) |
| [FBgn0035385](http://flybase.org/cgi-bin/fbidq.html?FBgn0035385) | FMRFamide Receptor | [FMRFaR](http://flybase.org/cgi-bin/fbidq.html?FBgn0035385) |
| [FBgn0030003](http://flybase.org/cgi-bin/fbidq.html?FBgn0030003) | - | [CG2116](http://flybase.org/cgi-bin/fbidq.html?FBgn0030003) |
| [FBgn0028646](http://flybase.org/cgi-bin/fbidq.html?FBgn0028646) | aralar1 | [aralar1](http://flybase.org/cgi-bin/fbidq.html?FBgn0028646) |
| [FBgn0035213](http://flybase.org/cgi-bin/fbidq.html?FBgn0035213) | - | [CG2199](http://flybase.org/cgi-bin/fbidq.html?FBgn0035213) |
| [FBgn0039668](http://flybase.org/cgi-bin/fbidq.html?FBgn0039668) | Trc8 | [Trc8](http://flybase.org/cgi-bin/fbidq.html?FBgn0039668) |
| [FBgn0010750](http://flybase.org/cgi-bin/fbidq.html?FBgn0010750) | antimeros | [atms](http://flybase.org/cgi-bin/fbidq.html?FBgn0010750) |
| [FBgn0032863](http://flybase.org/cgi-bin/fbidq.html?FBgn0032863) | Cell division cycle 23 ortholog | [Cdc23](http://flybase.org/cgi-bin/fbidq.html?FBgn0032863) |
| [FBgn0010391](http://flybase.org/cgi-bin/fbidq.html?FBgn0010391) | GTP-binding protein | [Gtp-bp](http://flybase.org/cgi-bin/fbidq.html?FBgn0010391) |
| [FBgn0024997](http://flybase.org/cgi-bin/fbidq.html?FBgn0024997) | - | [CG2681](http://flybase.org/cgi-bin/fbidq.html?FBgn0024997) |
| [FBgn0024352](http://flybase.org/cgi-bin/fbidq.html?FBgn0024352) | Hsp70/Hsp90 organizing protein homolog | [Hop](http://flybase.org/cgi-bin/fbidq.html?FBgn0024352) |
| [FBgn0029725](http://flybase.org/cgi-bin/fbidq.html?FBgn0029725) | - | [CG2871](http://flybase.org/cgi-bin/fbidq.html?FBgn0029725) |
| [FBgn0061356](http://flybase.org/cgi-bin/fbidq.html?FBgn0061356) | - | [CG18003](http://flybase.org/cgi-bin/fbidq.html?FBgn0061356) |
| [FBgn0050035](http://flybase.org/cgi-bin/fbidq.html?FBgn0050035) | Trehalose transporter 1-1 | [Tret1-1](http://flybase.org/cgi-bin/fbidq.html?FBgn0050035) |
| [FBgn0050058](http://flybase.org/cgi-bin/fbidq.html?FBgn0050058) | - | [CG30058](http://flybase.org/cgi-bin/fbidq.html?FBgn0050058) |
| [FBgn0050121](http://flybase.org/cgi-bin/fbidq.html?FBgn0050121) | - | [CR30121](http://flybase.org/cgi-bin/fbidq.html?FBgn0050121) |
| [FBgn0034543](http://flybase.org/cgi-bin/fbidq.html?FBgn0034543) | - | [CG30152](http://flybase.org/cgi-bin/fbidq.html?FBgn0034543) |
| [FBgn0050158](http://flybase.org/cgi-bin/fbidq.html?FBgn0050158) | - | [CG30158](http://flybase.org/cgi-bin/fbidq.html?FBgn0050158) |
| [FBgn0050159](http://flybase.org/cgi-bin/fbidq.html?FBgn0050159) | - | [CG30159](http://flybase.org/cgi-bin/fbidq.html?FBgn0050159) |
| [FBgn0263030](http://flybase.org/cgi-bin/fbidq.html?FBgn0263030) | - | [CG43325](http://flybase.org/cgi-bin/fbidq.html?FBgn0263030) |
| [FBgn0050281](http://flybase.org/cgi-bin/fbidq.html?FBgn0050281) | - | [CG30281](http://flybase.org/cgi-bin/fbidq.html?FBgn0050281) |
| [FBgn0050289](http://flybase.org/cgi-bin/fbidq.html?FBgn0050289) | - | [CG30289](http://flybase.org/cgi-bin/fbidq.html?FBgn0050289) |
| [FBgn0029928](http://flybase.org/cgi-bin/fbidq.html?FBgn0029928) | - | [CG3032](http://flybase.org/cgi-bin/fbidq.html?FBgn0029928) |
| [FBgn0000330](http://flybase.org/cgi-bin/fbidq.html?FBgn0000330) | carmine | [cm](http://flybase.org/cgi-bin/fbidq.html?FBgn0000330) |
| [FBgn0050383](http://flybase.org/cgi-bin/fbidq.html?FBgn0050383) | - | [CG30383](http://flybase.org/cgi-bin/fbidq.html?FBgn0050383) |
| [FBgn0050416](http://flybase.org/cgi-bin/fbidq.html?FBgn0050416) | - | [CG30416](http://flybase.org/cgi-bin/fbidq.html?FBgn0050416) |
| [FBgn0259247](http://flybase.org/cgi-bin/fbidq.html?FBgn0259247) | laccase 2 | [laccase2](http://flybase.org/cgi-bin/fbidq.html?FBgn0259247) |
| [FBgn0050460](http://flybase.org/cgi-bin/fbidq.html?FBgn0050460) | - | [CG30460](http://flybase.org/cgi-bin/fbidq.html?FBgn0050460) |
| [FBgn0050463](http://flybase.org/cgi-bin/fbidq.html?FBgn0050463) | - | [CG30463](http://flybase.org/cgi-bin/fbidq.html?FBgn0050463) |
| [FBgn0050485](http://flybase.org/cgi-bin/fbidq.html?FBgn0050485) | - | [CG30485](http://flybase.org/cgi-bin/fbidq.html?FBgn0050485) |
| [FBgn0000147](http://flybase.org/cgi-bin/fbidq.html?FBgn0000147) | aurora | [aur](http://flybase.org/cgi-bin/fbidq.html?FBgn0000147) |
| [FBgn0051182](http://flybase.org/cgi-bin/fbidq.html?FBgn0051182) | - | [CG31182](http://flybase.org/cgi-bin/fbidq.html?FBgn0051182) |
| [FBgn0051226](http://flybase.org/cgi-bin/fbidq.html?FBgn0051226) | - | [CG31226](http://flybase.org/cgi-bin/fbidq.html?FBgn0051226) |
| FBgn0051294 | - | CG31294 |
| [FBgn0051324](http://flybase.org/cgi-bin/fbidq.html?FBgn0051324) | - | [CG31324](http://flybase.org/cgi-bin/fbidq.html?FBgn0051324) |
| [FBgn0051327](http://flybase.org/cgi-bin/fbidq.html?FBgn0051327) | - | [CG31327](http://flybase.org/cgi-bin/fbidq.html?FBgn0051327) |
| [FBgn0051373](http://flybase.org/cgi-bin/fbidq.html?FBgn0051373) | - | [CG31373](http://flybase.org/cgi-bin/fbidq.html?FBgn0051373) |
| [FBgn0028396](http://flybase.org/cgi-bin/fbidq.html?FBgn0028396) | Turandot A | [TotA](http://flybase.org/cgi-bin/fbidq.html?FBgn0028396) |
| [FBgn0010263](http://flybase.org/cgi-bin/fbidq.html?FBgn0010263) | RNA-binding protein 9 | [Rbp9](http://flybase.org/cgi-bin/fbidq.html?FBgn0010263) |
| [FBgn0046876](http://flybase.org/cgi-bin/fbidq.html?FBgn0046876) | Odorant-binding protein 83ef | [Obp83ef](http://flybase.org/cgi-bin/fbidq.html?FBgn0046876) |
| [FBgn0033079](http://flybase.org/cgi-bin/fbidq.html?FBgn0033079) | Flavin-containing monooxygenase 2 | [Fmo-2](http://flybase.org/cgi-bin/fbidq.html?FBgn0033079) |
| [FBgn0051815](http://flybase.org/cgi-bin/fbidq.html?FBgn0051815) | - | [CG31815](http://flybase.org/cgi-bin/fbidq.html?FBgn0051815) |
| [FBgn0028543](http://flybase.org/cgi-bin/fbidq.html?FBgn0028543) | Nimrod B2 | [NimB2](http://flybase.org/cgi-bin/fbidq.html?FBgn0028543) |
| [FBgn0051870](http://flybase.org/cgi-bin/fbidq.html?FBgn0051870) | - | [CG31870](http://flybase.org/cgi-bin/fbidq.html?FBgn0051870) |
| [FBgn0260750](http://flybase.org/cgi-bin/fbidq.html?FBgn0260750) | Multi-substrate lipid kinase | [Mulk](http://flybase.org/cgi-bin/fbidq.html?FBgn0260750) |
| [FBgn0051909](http://flybase.org/cgi-bin/fbidq.html?FBgn0051909) | - | [CG31909](http://flybase.org/cgi-bin/fbidq.html?FBgn0051909) |
| [FBgn0051988](http://flybase.org/cgi-bin/fbidq.html?FBgn0051988) | - | [CG31988](http://flybase.org/cgi-bin/fbidq.html?FBgn0051988) |
| FBgn0052058 | \|  \| Ionotropic receptor 67c \| \| --- \| --- \| | Ir67c |
| [FBgn0052282](http://flybase.org/cgi-bin/fbidq.html?FBgn0052282) | Drosomycin-like 4 | [Drsl4](http://flybase.org/cgi-bin/fbidq.html?FBgn0052282) |
| [FBgn0052284](http://flybase.org/cgi-bin/fbidq.html?FBgn0052284) | - | [CG32284](http://flybase.org/cgi-bin/fbidq.html?FBgn0052284) |
| [FBgn0040298](http://flybase.org/cgi-bin/fbidq.html?FBgn0040298) | Myt1 | [Myt1](http://flybase.org/cgi-bin/fbidq.html?FBgn0040298) |
| [FBgn0052568](http://flybase.org/cgi-bin/fbidq.html?FBgn0052568) | - | [CG32568](http://flybase.org/cgi-bin/fbidq.html?FBgn0052568) |
| [FBgn0052640](http://flybase.org/cgi-bin/fbidq.html?FBgn0052640) | - | [CG32640](http://flybase.org/cgi-bin/fbidq.html?FBgn0052640) |
| [FBgn0052692](http://flybase.org/cgi-bin/fbidq.html?FBgn0052692) | - | [CG32692](http://flybase.org/cgi-bin/fbidq.html?FBgn0052692) |
| [FBgn0052834](http://flybase.org/cgi-bin/fbidq.html?FBgn0052834) | - | [CG32834](http://flybase.org/cgi-bin/fbidq.html?FBgn0052834) |
| [FBgn0041233](http://flybase.org/cgi-bin/fbidq.html?FBgn0041233) | Gustatory receptor 59e | [Gr59e](http://flybase.org/cgi-bin/fbidq.html?FBgn0041233) |
| [FBgn0069056](http://flybase.org/cgi-bin/fbidq.html?FBgn0069056) | - | [CG33226](http://flybase.org/cgi-bin/fbidq.html?FBgn0069056) |
| [FBgn0053281](http://flybase.org/cgi-bin/fbidq.html?FBgn0053281) | - | [CG33281](http://flybase.org/cgi-bin/fbidq.html?FBgn0053281) |
| [FBgn0067311](http://flybase.org/cgi-bin/fbidq.html?FBgn0067311) | Chemosensory protein B 38b | [CheB38b](http://flybase.org/cgi-bin/fbidq.html?FBgn0067311) |
| [FBgn0067629](http://flybase.org/cgi-bin/fbidq.html?FBgn0067629) | - | [CG33332](http://flybase.org/cgi-bin/fbidq.html?FBgn0067629) |
| [FBgn0053335](http://flybase.org/cgi-bin/fbidq.html?FBgn0053335) | - | [CG33335](http://flybase.org/cgi-bin/fbidq.html?FBgn0053335) |
| [FBgn0053467](http://flybase.org/cgi-bin/fbidq.html?FBgn0053467) | - | [CG33467](http://flybase.org/cgi-bin/fbidq.html?FBgn0053467) |
| [FBgn0031513](http://flybase.org/cgi-bin/fbidq.html?FBgn0031513) | - | [CG3347](http://flybase.org/cgi-bin/fbidq.html?FBgn0031513) |
| [FBgn0038236](http://flybase.org/cgi-bin/fbidq.html?FBgn0038236) | Cyp313a1 | [Cyp313a1](http://flybase.org/cgi-bin/fbidq.html?FBgn0038236) |
| [FBgn0054010](http://flybase.org/cgi-bin/fbidq.html?FBgn0054010) | - | [CG34010](http://flybase.org/cgi-bin/fbidq.html?FBgn0054010) |
| [FBgn0035005](http://flybase.org/cgi-bin/fbidq.html?FBgn0035005) | - | [CG3483](http://flybase.org/cgi-bin/fbidq.html?FBgn0035005) |
| [FBgn0027571](http://flybase.org/cgi-bin/fbidq.html?FBgn0027571) | - | [CG3523](http://flybase.org/cgi-bin/fbidq.html?FBgn0027571) |
| [FBgn0031562](http://flybase.org/cgi-bin/fbidq.html?FBgn0031562) | - | [CG3604](http://flybase.org/cgi-bin/fbidq.html?FBgn0031562) |
| [FBgn0040397](http://flybase.org/cgi-bin/fbidq.html?FBgn0040397) | - | [CG3655](http://flybase.org/cgi-bin/fbidq.html?FBgn0040397) |
| [FBgn0010078](http://flybase.org/cgi-bin/fbidq.html?FBgn0010078) | Ribosomal protein L23 | [RpL23](http://flybase.org/cgi-bin/fbidq.html?FBgn0010078) |
| [FBgn0014010](http://flybase.org/cgi-bin/fbidq.html?FBgn0014010) | Rab5 | [Rab5](http://flybase.org/cgi-bin/fbidq.html?FBgn0014010) |
| \|  \| FBgn0040350 \| \| --- \| --- \| | - | CG3690 |
| [FBgn0028507](http://flybase.org/cgi-bin/fbidq.html?FBgn0028507) | - | [CG3793](http://flybase.org/cgi-bin/fbidq.html?FBgn0028507) |
| [FBgn0027524](http://flybase.org/cgi-bin/fbidq.html?FBgn0027524) | - | [CG3909](http://flybase.org/cgi-bin/fbidq.html?FBgn0027524) |
| [FBgn0003507](http://flybase.org/cgi-bin/fbidq.html?FBgn0003507) | serpent | [srp](http://flybase.org/cgi-bin/fbidq.html?FBgn0003507) |
| [FBgn0025117](http://flybase.org/cgi-bin/fbidq.html?FBgn0025117) | uninitiated | [und](http://flybase.org/cgi-bin/fbidq.html?FBgn0025117) |
| [FBgn0000042](http://flybase.org/cgi-bin/fbidq.html?FBgn0000042) | Actin 5C | [Act5C](http://flybase.org/cgi-bin/fbidq.html?FBgn0000042) |
| [FBgn0020385](http://flybase.org/cgi-bin/fbidq.html?FBgn0020385) | pugilist | [pug](http://flybase.org/cgi-bin/fbidq.html?FBgn0020385) |
| [FBgn0025558](http://flybase.org/cgi-bin/fbidq.html?FBgn0025558) | - | [CG4101](http://flybase.org/cgi-bin/fbidq.html?FBgn0025558) |
| [FBgn0036640](http://flybase.org/cgi-bin/fbidq.html?FBgn0036640) | nuclear RNA export factor 2 | [nxf2](http://flybase.org/cgi-bin/fbidq.html?FBgn0036640) |
| [FBgn0001986](http://flybase.org/cgi-bin/fbidq.html?FBgn0001986) | lethal (2) 35Df | [l(2)35Df](http://flybase.org/cgi-bin/fbidq.html?FBgn0001986) |
| [FBgn0029763](http://flybase.org/cgi-bin/fbidq.html?FBgn0029763) | - | [CG4165](http://flybase.org/cgi-bin/fbidq.html?FBgn0029763) |
| [FBgn0028926](http://flybase.org/cgi-bin/fbidq.html?FBgn0028926) | Negative Cofactor 2beta | [NC2beta](http://flybase.org/cgi-bin/fbidq.html?FBgn0028926) |
| FBgn0003416 | \|  \| small wing \| \| --- \| --- \| | sl |
| [FBgn0264855](http://flybase.org/cgi-bin/fbidq.html?FBgn0264855) | Adaptor Protein complex 2, alpha subunit | [AP-2alpha](http://flybase.org/cgi-bin/fbidq.html?FBgn0264855) |
| [FBgn0031407](http://flybase.org/cgi-bin/fbidq.html?FBgn0031407) | - | [CG4270](http://flybase.org/cgi-bin/fbidq.html?FBgn0031407) |
| [FBgn0032132](http://flybase.org/cgi-bin/fbidq.html?FBgn0032132) | - | [CG4382](http://flybase.org/cgi-bin/fbidq.html?FBgn0032132) |
| FBgn0032129 | \|  \| junctophilin \| \| --- \| --- \| | jp |
| [FBgn0030431](http://flybase.org/cgi-bin/fbidq.html?FBgn0030431) | - | [CG4407](http://flybase.org/cgi-bin/fbidq.html?FBgn0030431) |
| [FBgn0001226](http://flybase.org/cgi-bin/fbidq.html?FBgn0001226) | Heat shock protein 27 | [Hsp27](http://flybase.org/cgi-bin/fbidq.html?FBgn0001226) |
| [FBgn0020415](http://flybase.org/cgi-bin/fbidq.html?FBgn0020415) | Imaginal disc growth factor 2 | [Idgf2](http://flybase.org/cgi-bin/fbidq.html?FBgn0020415) |
| [FBgn0035971](http://flybase.org/cgi-bin/fbidq.html?FBgn0035971) | - | [CG4477](http://flybase.org/cgi-bin/fbidq.html?FBgn0035971) |
| [FBgn0264000](http://flybase.org/cgi-bin/fbidq.html?FBgn0264000) | Glutamate receptor IB | [GluRIB](http://flybase.org/cgi-bin/fbidq.html?FBgn0264000) |
| [FBgn0038369](http://flybase.org/cgi-bin/fbidq.html?FBgn0038369) | Actin-related protein 2/3 complex, subunit 3A | [Arpc3A](http://flybase.org/cgi-bin/fbidq.html?FBgn0038369) |
| [FBgn0038739](http://flybase.org/cgi-bin/fbidq.html?FBgn0038739) | - | [CG4686](http://flybase.org/cgi-bin/fbidq.html?FBgn0038739) |
| [FBgn0033820](http://flybase.org/cgi-bin/fbidq.html?FBgn0033820) | - | [CG4716](http://flybase.org/cgi-bin/fbidq.html?FBgn0033820) |
| [FBgn0039355](http://flybase.org/cgi-bin/fbidq.html?FBgn0039355) | - | [CG4730](http://flybase.org/cgi-bin/fbidq.html?FBgn0039355) |
| [FBgn0039357](http://flybase.org/cgi-bin/fbidq.html?FBgn0039357) | - | [CG4743](http://flybase.org/cgi-bin/fbidq.html?FBgn0039357) |
| [FBgn0037010](http://flybase.org/cgi-bin/fbidq.html?FBgn0037010) | - | [CG4825](http://flybase.org/cgi-bin/fbidq.html?FBgn0037010) |
| [FBgn0004117](http://flybase.org/cgi-bin/fbidq.html?FBgn0004117) | Tropomyosin 2 | [Tm2](http://flybase.org/cgi-bin/fbidq.html?FBgn0004117) |
| FBgn0035049 | Matrix metalloproteinase 1 | Mmp1 |
| [FBgn0011693](http://flybase.org/cgi-bin/fbidq.html?FBgn0011693) | Photoreceptor dehydrogenase | [Pdh](http://flybase.org/cgi-bin/fbidq.html?FBgn0011693) |
| [FBgn0020503](http://flybase.org/cgi-bin/fbidq.html?FBgn0020503) | Cytoplasmic linker protein 190 | [CLIP-190](http://flybase.org/cgi-bin/fbidq.html?FBgn0020503) |
| [FBgn0064237](http://flybase.org/cgi-bin/fbidq.html?FBgn0064237) | Imaginal disc growth factor 5 | [Idgf5](http://flybase.org/cgi-bin/fbidq.html?FBgn0064237) |
| [FBgn0013347](http://flybase.org/cgi-bin/fbidq.html?FBgn0013347) | Transcription-factor-IIA-S | [TfIIA-S](http://flybase.org/cgi-bin/fbidq.html?FBgn0013347) |
| [FBgn0038038](http://flybase.org/cgi-bin/fbidq.html?FBgn0038038) | - | [CG5167](http://flybase.org/cgi-bin/fbidq.html?FBgn0038038) |
| [FBgn0031907](http://flybase.org/cgi-bin/fbidq.html?FBgn0031907) | - | [CG5171](http://flybase.org/cgi-bin/fbidq.html?FBgn0031907) |
| [FBgn0031908](http://flybase.org/cgi-bin/fbidq.html?FBgn0031908) | - | [CG5177](http://flybase.org/cgi-bin/fbidq.html?FBgn0031908) |
| [FBgn0085486](http://flybase.org/cgi-bin/fbidq.html?FBgn0085486) | - | [CG34457](http://flybase.org/cgi-bin/fbidq.html?FBgn0085486) |
| [FBgn0032253](http://flybase.org/cgi-bin/fbidq.html?FBgn0032253) | - | [CG5322](http://flybase.org/cgi-bin/fbidq.html?FBgn0032253) |
| [FBgn0032407](http://flybase.org/cgi-bin/fbidq.html?FBgn0032407) | Peroxin 19 | [Pex19](http://flybase.org/cgi-bin/fbidq.html?FBgn0032407) |
| [FBgn0266671](http://flybase.org/cgi-bin/fbidq.html?FBgn0266671) | Sec6 ortholog (S. cerevisiae) | [Sec6](http://flybase.org/cgi-bin/fbidq.html?FBgn0266671) |
| [FBgn0262601](http://flybase.org/cgi-bin/fbidq.html?FBgn0262601) | Small ribonucleoprotein particle protein SmB | [SmB](http://flybase.org/cgi-bin/fbidq.html?FBgn0262601) |
| [FBgn0038943](http://flybase.org/cgi-bin/fbidq.html?FBgn0038943) | - | [CG5391](http://flybase.org/cgi-bin/fbidq.html?FBgn0038943) |
| [FBgn0031327](http://flybase.org/cgi-bin/fbidq.html?FBgn0031327) | - | [CG5397](http://flybase.org/cgi-bin/fbidq.html?FBgn0031327) |
| [FBgn0034914](http://flybase.org/cgi-bin/fbidq.html?FBgn0034914) | - | [CG5554](http://flybase.org/cgi-bin/fbidq.html?FBgn0034914) |
| [FBgn0036759](http://flybase.org/cgi-bin/fbidq.html?FBgn0036759) | - | [CG5577](http://flybase.org/cgi-bin/fbidq.html?FBgn0036759) |
| [FBgn0038838](http://flybase.org/cgi-bin/fbidq.html?FBgn0038838) | Turandot B | [TotB](http://flybase.org/cgi-bin/fbidq.html?FBgn0038838) |
| [FBgn0039529](http://flybase.org/cgi-bin/fbidq.html?FBgn0039529) | - | [CG5612](http://flybase.org/cgi-bin/fbidq.html?FBgn0039529) |
| [FBgn0032454](http://flybase.org/cgi-bin/fbidq.html?FBgn0032454) | - | [CG5787](http://flybase.org/cgi-bin/fbidq.html?FBgn0032454) |
| [FBgn0039130](http://flybase.org/cgi-bin/fbidq.html?FBgn0039130) | - | [CG5854](http://flybase.org/cgi-bin/fbidq.html?FBgn0039130) |
| [FBgn0027586](http://flybase.org/cgi-bin/fbidq.html?FBgn0027586) | - | [CG5867](http://flybase.org/cgi-bin/fbidq.html?FBgn0027586) |
| [FBgn0039380](http://flybase.org/cgi-bin/fbidq.html?FBgn0039380) | - | [CG5890](http://flybase.org/cgi-bin/fbidq.html?FBgn0039380) |
| [FBgn0004867](http://flybase.org/cgi-bin/fbidq.html?FBgn0004867) | Ribosomal protein S2 | [RpS2](http://flybase.org/cgi-bin/fbidq.html?FBgn0004867) |
| [FBgn0032447](http://flybase.org/cgi-bin/fbidq.html?FBgn0032447) | PICK1 | [PICK1](http://flybase.org/cgi-bin/fbidq.html?FBgn0032447) |
| [FBgn0004889](http://flybase.org/cgi-bin/fbidq.html?FBgn0004889) | twins | [tws](http://flybase.org/cgi-bin/fbidq.html?FBgn0004889) |
| [FBgn0034118](http://flybase.org/cgi-bin/fbidq.html?FBgn0034118) | Nucleoporin 62kD | [Nup62](http://flybase.org/cgi-bin/fbidq.html?FBgn0034118) |
| [FBgn0017579](http://flybase.org/cgi-bin/fbidq.html?FBgn0017579) | Ribosomal protein L14 | [RpL14](http://flybase.org/cgi-bin/fbidq.html?FBgn0017579) |
| [FBgn0036735](http://flybase.org/cgi-bin/fbidq.html?FBgn0036735) | Enhancer of decapping 3 | [Edc3](http://flybase.org/cgi-bin/fbidq.html?FBgn0036735) |
| [FBgn0019957](http://flybase.org/cgi-bin/fbidq.html?FBgn0019957) | NADH:ubiquinone reductase 42kD subunit precursor | [ND42](http://flybase.org/cgi-bin/fbidq.html?FBgn0019957) |
| [FBgn0266581](http://flybase.org/cgi-bin/fbidq.html?FBgn0266581) | pitchoune | [pit](http://flybase.org/cgi-bin/fbidq.html?FBgn0266581) |
| [FBgn0039453](http://flybase.org/cgi-bin/fbidq.html?FBgn0039453) | - | [CG6403](http://flybase.org/cgi-bin/fbidq.html?FBgn0039453) |
| [FBgn0036085](http://flybase.org/cgi-bin/fbidq.html?FBgn0036085) | - | [CG6527](http://flybase.org/cgi-bin/fbidq.html?FBgn0036085) |
| [FBgn0037842](http://flybase.org/cgi-bin/fbidq.html?FBgn0037842) | - | [CG6567](http://flybase.org/cgi-bin/fbidq.html?FBgn0037842) |
| [FBgn0034210](http://flybase.org/cgi-bin/fbidq.html?FBgn0034210) | - | [CG6568](http://flybase.org/cgi-bin/fbidq.html?FBgn0034210) |
| [FBgn0038303](http://flybase.org/cgi-bin/fbidq.html?FBgn0038303) | Shal Interactor of Di-Leucine Motif | [SIDL](http://flybase.org/cgi-bin/fbidq.html?FBgn0038303) |
| [FBgn0032638](http://flybase.org/cgi-bin/fbidq.html?FBgn0032638) | - | [CG6639](http://flybase.org/cgi-bin/fbidq.html?FBgn0032638) |
| [FBgn0039056](http://flybase.org/cgi-bin/fbidq.html?FBgn0039056) | Centaurin beta 1A | [CenB1A](http://flybase.org/cgi-bin/fbidq.html?FBgn0039056) |
| [FBgn0040475](http://flybase.org/cgi-bin/fbidq.html?FBgn0040475) | SH3PX1 | [SH3PX1](http://flybase.org/cgi-bin/fbidq.html?FBgn0040475) |
| [FBgn0038076](http://flybase.org/cgi-bin/fbidq.html?FBgn0038076) | Cyp313a4 | [Cyp313a4](http://flybase.org/cgi-bin/fbidq.html?FBgn0038076) |
| [FBgn0038294](http://flybase.org/cgi-bin/fbidq.html?FBgn0038294) | Myofilin | [Mf](http://flybase.org/cgi-bin/fbidq.html?FBgn0038294) |
| [FBgn0037921](http://flybase.org/cgi-bin/fbidq.html?FBgn0037921) | - | [CG6808](http://flybase.org/cgi-bin/fbidq.html?FBgn0037921) |
| [FBgn0036825](http://flybase.org/cgi-bin/fbidq.html?FBgn0036825) | Ribosomal protein L26 | [RpL26](http://flybase.org/cgi-bin/fbidq.html?FBgn0036825) |
| [FBgn0036952](http://flybase.org/cgi-bin/fbidq.html?FBgn0036952) | - | [CG6933](http://flybase.org/cgi-bin/fbidq.html?FBgn0036952) |
| [FBgn0263352](http://flybase.org/cgi-bin/fbidq.html?FBgn0263352) | Upstream of N-ras | [Unr](http://flybase.org/cgi-bin/fbidq.html?FBgn0263352) |
| [FBgn0030093](http://flybase.org/cgi-bin/fbidq.html?FBgn0030093) | dalao | [dalao](http://flybase.org/cgi-bin/fbidq.html?FBgn0030093) |
| [FBgn0004177](http://flybase.org/cgi-bin/fbidq.html?FBgn0004177) | microtubule star | [mts](http://flybase.org/cgi-bin/fbidq.html?FBgn0004177) |
| [FBgn0265102](http://flybase.org/cgi-bin/fbidq.html?FBgn0265102) | Outer segment 1 | [Oseg1](http://flybase.org/cgi-bin/fbidq.html?FBgn0265102) |
| [FBgn0003961](http://flybase.org/cgi-bin/fbidq.html?FBgn0003961) | Urate oxidase | [Uro](http://flybase.org/cgi-bin/fbidq.html?FBgn0003961) |
| [FBgn0037098](http://flybase.org/cgi-bin/fbidq.html?FBgn0037098) | WNK homolog | [Wnk](http://flybase.org/cgi-bin/fbidq.html?FBgn0037098) |
| [FBgn0011723](http://flybase.org/cgi-bin/fbidq.html?FBgn0011723) | brachyenteron | [byn](http://flybase.org/cgi-bin/fbidq.html?FBgn0011723) |
| [FBgn0036501](http://flybase.org/cgi-bin/fbidq.html?FBgn0036501) | - | [CG7272](http://flybase.org/cgi-bin/fbidq.html?FBgn0036501) |
| [FBgn0031713](http://flybase.org/cgi-bin/fbidq.html?FBgn0031713) | - | [CG7277](http://flybase.org/cgi-bin/fbidq.html?FBgn0031713) |
| [FBgn0037074](http://flybase.org/cgi-bin/fbidq.html?FBgn0037074) | - | [CG7324](http://flybase.org/cgi-bin/fbidq.html?FBgn0037074) |
| [FBgn0023076](http://flybase.org/cgi-bin/fbidq.html?FBgn0023076) | Clock | [Clk](http://flybase.org/cgi-bin/fbidq.html?FBgn0023076) |
| [FBgn0027085](http://flybase.org/cgi-bin/fbidq.html?FBgn0027085) | Leucyl-tRNA synthetase | [Aats-leu](http://flybase.org/cgi-bin/fbidq.html?FBgn0027085) |
| [FBgn0004514](http://flybase.org/cgi-bin/fbidq.html?FBgn0004514) | Octopamine-Tyramine receptor | [Oct-TyrR](http://flybase.org/cgi-bin/fbidq.html?FBgn0004514) |
| [FBgn0261535](http://flybase.org/cgi-bin/fbidq.html?FBgn0261535) | lethal (2) 34Fd | [l(2)34Fd](http://flybase.org/cgi-bin/fbidq.html?FBgn0261535) |
| [FBgn0038523](http://flybase.org/cgi-bin/fbidq.html?FBgn0038523) | - | [CG7587](http://flybase.org/cgi-bin/fbidq.html?FBgn0038523) |
| [FBgn0027518](http://flybase.org/cgi-bin/fbidq.html?FBgn0027518) | - | [CG7609](http://flybase.org/cgi-bin/fbidq.html?FBgn0027518) |
| [FBgn0024956](http://flybase.org/cgi-bin/fbidq.html?FBgn0024956) | Menage a trois 1 ortholog (H. sapiens) | [Mat1](http://flybase.org/cgi-bin/fbidq.html?FBgn0024956) |
| [FBgn0001217](http://flybase.org/cgi-bin/fbidq.html?FBgn0001217) | Heat shock protein cognate 2 | [Hsc70-2](http://flybase.org/cgi-bin/fbidq.html?FBgn0001217) |
| [FBgn0032021](http://flybase.org/cgi-bin/fbidq.html?FBgn0032021) | - | [CG7781](http://flybase.org/cgi-bin/fbidq.html?FBgn0032021) |
| [FBgn0010424](http://flybase.org/cgi-bin/fbidq.html?FBgn0010424) | Troponin C at 73F | [TpnC73F](http://flybase.org/cgi-bin/fbidq.html?FBgn0010424) |
| [FBgn0038890](http://flybase.org/cgi-bin/fbidq.html?FBgn0038890) | - | [CG7956](http://flybase.org/cgi-bin/fbidq.html?FBgn0038890) |
| [FBgn0020887](http://flybase.org/cgi-bin/fbidq.html?FBgn0020887) | Su(z)12 | [Su(z)12](http://flybase.org/cgi-bin/fbidq.html?FBgn0020887) |
| [FBgn0036916](http://flybase.org/cgi-bin/fbidq.html?FBgn0036916) | Mtr3 | [Mtr3](http://flybase.org/cgi-bin/fbidq.html?FBgn0036916) |
| [FBgn0037614](http://flybase.org/cgi-bin/fbidq.html?FBgn0037614) | - | [CG8116](http://flybase.org/cgi-bin/fbidq.html?FBgn0037614) |
| [FBgn0011660](http://flybase.org/cgi-bin/fbidq.html?FBgn0011660) | Pms2 | [Pms2](http://flybase.org/cgi-bin/fbidq.html?FBgn0011660) |
| [FBgn0026666](http://flybase.org/cgi-bin/fbidq.html?FBgn0026666) | lethal (1) G0136 | [l(1)G0136](http://flybase.org/cgi-bin/fbidq.html?FBgn0026666) |
| [FBgn0027513](http://flybase.org/cgi-bin/fbidq.html?FBgn0027513) | anastral spindle 2 | [ana2](http://flybase.org/cgi-bin/fbidq.html?FBgn0027513) |
| [FBgn0035823](http://flybase.org/cgi-bin/fbidq.html?FBgn0035823) | eIF4E-5 | [eIF4E-5](http://flybase.org/cgi-bin/fbidq.html?FBgn0035823) |
| [FBgn0037718](http://flybase.org/cgi-bin/fbidq.html?FBgn0037718) | P58IPK | [P58IPK](http://flybase.org/cgi-bin/fbidq.html?FBgn0037718) |
| [FBgn0034059](http://flybase.org/cgi-bin/fbidq.html?FBgn0034059) | - | [CG8320](http://flybase.org/cgi-bin/fbidq.html?FBgn0034059) |
| [FBgn0015754](http://flybase.org/cgi-bin/fbidq.html?FBgn0015754) | Lissencephaly-1 | [Lis-1](http://flybase.org/cgi-bin/fbidq.html?FBgn0015754) |
| [FBgn0033725](http://flybase.org/cgi-bin/fbidq.html?FBgn0033725) | Cuticular protein 49Ac | [Cpr49Ac](http://flybase.org/cgi-bin/fbidq.html?FBgn0033725) |
| [FBgn0035711](http://flybase.org/cgi-bin/fbidq.html?FBgn0035711) | - | [CG8519](http://flybase.org/cgi-bin/fbidq.html?FBgn0035711) |
| [FBgn0014011](http://flybase.org/cgi-bin/fbidq.html?FBgn0014011) | Rac2 | [Rac2](http://flybase.org/cgi-bin/fbidq.html?FBgn0014011) |
| [FBgn0261239](http://flybase.org/cgi-bin/fbidq.html?FBgn0261239) | Hormone receptor-like in 39 | [Hr39](http://flybase.org/cgi-bin/fbidq.html?FBgn0261239) |
| [FBgn0026722](http://flybase.org/cgi-bin/fbidq.html?FBgn0026722) | drosha | [drosha](http://flybase.org/cgi-bin/fbidq.html?FBgn0026722) |
| [FBgn0010213](http://flybase.org/cgi-bin/fbidq.html?FBgn0010213) | Superoxide dismutase 2 (Mn) | [Sod2](http://flybase.org/cgi-bin/fbidq.html?FBgn0010213) |
| [FBgn0030685](http://flybase.org/cgi-bin/fbidq.html?FBgn0030685) | GTPase regulator associated with focal adhesion kinase ortholog (H. sapiens) | [Graf](http://flybase.org/cgi-bin/fbidq.html?FBgn0030685) |
| [FBgn0030691](http://flybase.org/cgi-bin/fbidq.html?FBgn0030691) | EFHC1 homologue 1 | [Efhc1.1](http://flybase.org/cgi-bin/fbidq.html?FBgn0030691) |
| [FBgn0004828](http://flybase.org/cgi-bin/fbidq.html?FBgn0004828) | Histone H3.3B | [His3.3B](http://flybase.org/cgi-bin/fbidq.html?FBgn0004828) |
| [FBgn0035203](http://flybase.org/cgi-bin/fbidq.html?FBgn0035203) | - | [CG9149](http://flybase.org/cgi-bin/fbidq.html?FBgn0035203) |
| [FBgn0032908](http://flybase.org/cgi-bin/fbidq.html?FBgn0032908) | - | [CG9270](http://flybase.org/cgi-bin/fbidq.html?FBgn0032908) |
| \|  \| FBgn0032059 \| \| --- \| --- \| | Prenyl-binding protein | PrBP |
| [FBgn0034681](http://flybase.org/cgi-bin/fbidq.html?FBgn0034681) | - | [CG9308](http://flybase.org/cgi-bin/fbidq.html?FBgn0034681) |
| [FBgn0036891](http://flybase.org/cgi-bin/fbidq.html?FBgn0036891) | - | [CG9372](http://flybase.org/cgi-bin/fbidq.html?FBgn0036891) |
| [FBgn0036877](http://flybase.org/cgi-bin/fbidq.html?FBgn0036877) | - | [CG9452](http://flybase.org/cgi-bin/fbidq.html?FBgn0036877) |
| [FBgn0265137](http://flybase.org/cgi-bin/fbidq.html?FBgn0265137) | Serpin 42Da | [Spn42Da](http://flybase.org/cgi-bin/fbidq.html?FBgn0265137) |
| [FBgn0085427](http://flybase.org/cgi-bin/fbidq.html?FBgn0085427) | - | [CG34398](http://flybase.org/cgi-bin/fbidq.html?FBgn0085427) |
| [FBgn0259715](http://flybase.org/cgi-bin/fbidq.html?FBgn0259715) | - | [CG42369](http://flybase.org/cgi-bin/fbidq.html?FBgn0259715) |
| [FBgn0259749](http://flybase.org/cgi-bin/fbidq.html?FBgn0259749) | mummy | [mmy](http://flybase.org/cgi-bin/fbidq.html?FBgn0259749) |
| [FBgn0031821](http://flybase.org/cgi-bin/fbidq.html?FBgn0031821) | Kynurenine formamidase | [KFase](http://flybase.org/cgi-bin/fbidq.html?FBgn0031821) |
| [FBgn0031086](http://flybase.org/cgi-bin/fbidq.html?FBgn0031086) | forkhead domain 19B | [fd19B](http://flybase.org/cgi-bin/fbidq.html?FBgn0031086) |
| [FBgn0067861](http://flybase.org/cgi-bin/fbidq.html?FBgn0067861) | Sperm-specific dynein intermediate chain 1 | [Sdic1](http://flybase.org/cgi-bin/fbidq.html?FBgn0067861) |
| [FBgn0038360](http://flybase.org/cgi-bin/fbidq.html?FBgn0038360) | - | [CG9590](http://flybase.org/cgi-bin/fbidq.html?FBgn0038360) |
| [FBgn0265101](http://flybase.org/cgi-bin/fbidq.html?FBgn0265101) | suppressor-of-G2-allele-of-skp1 | [Sgt1](http://flybase.org/cgi-bin/fbidq.html?FBgn0265101) |
| [FBgn0024326](http://flybase.org/cgi-bin/fbidq.html?FBgn0024326) | MAP kinase kinase 4 | [Mkk4](http://flybase.org/cgi-bin/fbidq.html?FBgn0024326) |
| [FBgn0003060](http://flybase.org/cgi-bin/fbidq.html?FBgn0003060) | - | [CG9757](http://flybase.org/cgi-bin/fbidq.html?FBgn0003060) |
| [FBgn0037261](http://flybase.org/cgi-bin/fbidq.html?FBgn0037261) | - | [CG9775](http://flybase.org/cgi-bin/fbidq.html?FBgn0037261) |
| [FBgn0027360](http://flybase.org/cgi-bin/fbidq.html?FBgn0027360) | Translocase of inner membrane 10 | [Tim10](http://flybase.org/cgi-bin/fbidq.html?FBgn0027360) |
| [FBgn0038201](http://flybase.org/cgi-bin/fbidq.html?FBgn0038201) | Pyrokinin 1 receptor | [PK1-R](http://flybase.org/cgi-bin/fbidq.html?FBgn0038201) |
| [FBgn0003346](http://flybase.org/cgi-bin/fbidq.html?FBgn0003346) | Ran GTPase activating protein | [RanGAP](http://flybase.org/cgi-bin/fbidq.html?FBgn0003346) |
| [FBgn0263396](http://flybase.org/cgi-bin/fbidq.html?FBgn0263396) | squid | [sqd](http://flybase.org/cgi-bin/fbidq.html?FBgn0263396) |
