# Supplementary material for: Transcriptional Regionalization of the Fruit Fly’s Airway Epithelium
Source: PLoS One. 2014 Jul 14;9(7):e102534. doi: 10.1371/journal.pone.0102534 (PMC4097054; doi:10.1371/journal.pone.0102534)
Supplement: Table S3 — Genes predominantly expressed in dorsal trunks AND regulated by hypoxia. (DOCX) [file pone.0102534.s004.docx]

| **CG** | **Flybase ID** | **name** | **Symbol** |
| --- | --- | --- | --- |
| [CG17245](http://flybase.org/cgi-bin/fbidq.html?FBgn0025740) | [FBgn0025740](http://flybase.org/cgi-bin/fbidq.html?FBgn0025740) | plexin B | [plexB](http://flybase.org/cgi-bin/fbidq.html?FBgn0025740) |
| [CG17648](http://flybase.org/cgi-bin/fbidq.html?FBgn0031364) | [FBgn0031364](http://flybase.org/cgi-bin/fbidq.html?FBgn0031364) | - | [CG17648](http://flybase.org/cgi-bin/fbidq.html?FBgn0031364) |
| [CG18468](http://flybase.org/cgi-bin/fbidq.html?FBgn0034217) | [FBgn0034217](http://flybase.org/cgi-bin/fbidq.html?FBgn0034217) | Lethal hybrid rescue | [Lhr](http://flybase.org/cgi-bin/fbidq.html?FBgn0034217) |
| [CG18540](http://flybase.org/cgi-bin/fbidq.html?FBgn0034326) | [FBgn0034326](http://flybase.org/cgi-bin/fbidq.html?FBgn0034326) | - | [CG18540](http://flybase.org/cgi-bin/fbidq.html?FBgn0034326) |
| [CG18554](http://flybase.org/cgi-bin/fbidq.html?FBgn0250910) | [FBgn0250910](http://flybase.org/cgi-bin/fbidq.html?FBgn0250910) | Octopamine beta3 receptor | [Octbeta3R](http://flybase.org/cgi-bin/fbidq.html?FBgn0250910) |
| [CG3004](http://flybase.org/cgi-bin/fbidq.html?FBgn0264691) | [FBgn0264691](http://flybase.org/cgi-bin/fbidq.html?FBgn0264691) | - | [Lst8](http://flybase.org/cgi-bin/fbidq.html?FBgn0264691) |
| [CG31104](http://flybase.org/cgi-bin/fbidq.html?FBgn0051104) | [FBgn0051104](http://flybase.org/cgi-bin/fbidq.html?FBgn0051104) | - | [CG31104](http://flybase.org/cgi-bin/fbidq.html?FBgn0051104) |
| [CG31122](http://flybase.org/cgi-bin/fbidq.html?FBgn0051122) | [FBgn0051122](http://flybase.org/cgi-bin/fbidq.html?FBgn0051122) | - | [CG31122](http://flybase.org/cgi-bin/fbidq.html?FBgn0051122) |
| [CG31171](http://flybase.org/cgi-bin/fbidq.html?FBgn0051171) | [FBgn0051171](http://flybase.org/cgi-bin/fbidq.html?FBgn0051171) | - | [CG31171](http://flybase.org/cgi-bin/fbidq.html?FBgn0051171) |
| [CG32138](http://flybase.org/cgi-bin/fbidq.html?FBgn0052138) | [FBgn0052138](http://flybase.org/cgi-bin/fbidq.html?FBgn0052138) | - | [CG32138](http://flybase.org/cgi-bin/fbidq.html?FBgn0052138) |
| [CG3265](http://flybase.org/cgi-bin/fbidq.html?FBgn0027066) | [FBgn0027066](http://flybase.org/cgi-bin/fbidq.html?FBgn0027066) | Eb1 | [Eb1](http://flybase.org/cgi-bin/fbidq.html?FBgn0027066) |
| [CG32700](http://flybase.org/cgi-bin/fbidq.html?FBgn0052700) | [FBgn0052700](http://flybase.org/cgi-bin/fbidq.html?FBgn0052700) | - | [CG32700](http://flybase.org/cgi-bin/fbidq.html?FBgn0052700) |
| [CG40300](http://flybase.org/cgi-bin/fbidq.html?FBgn0250816) | [FBgn0250816](http://flybase.org/cgi-bin/fbidq.html?FBgn0250816) | Argonaute 3 | [AGO3](http://flybase.org/cgi-bin/fbidq.html?FBgn0250816) |
| [CG4925](http://flybase.org/cgi-bin/fbidq.html?FBgn0036614) | [FBgn0036614](http://flybase.org/cgi-bin/fbidq.html?FBgn0036614) | - | [CG4925](http://flybase.org/cgi-bin/fbidq.html?FBgn0036614) |
| [CG5269](http://flybase.org/cgi-bin/fbidq.html?FBgn0262468) | [FBgn0262468](http://flybase.org/cgi-bin/fbidq.html?FBgn0262468) | vibrator | [vib](http://flybase.org/cgi-bin/fbidq.html?FBgn0262468) |
| [CG5676](http://flybase.org/cgi-bin/fbidq.html?FBgn0032200) | [FBgn0032200](http://flybase.org/cgi-bin/fbidq.html?FBgn0032200) | - | [CG5676](http://flybase.org/cgi-bin/fbidq.html?FBgn0032200) |
| [CG5730](http://flybase.org/cgi-bin/fbidq.html?FBgn0000083) | [FBgn0000083](http://flybase.org/cgi-bin/fbidq.html?FBgn0000083) | Annexin B9 | [AnxB9](http://flybase.org/cgi-bin/fbidq.html?FBgn0000083) |
| [CG5915](http://flybase.org/cgi-bin/fbidq.html?FBgn0015795) | [FBgn0015795](http://flybase.org/cgi-bin/fbidq.html?FBgn0015795) | Rab7 | [Rab7](http://flybase.org/cgi-bin/fbidq.html?FBgn0015795) |
| [CG5996](http://flybase.org/cgi-bin/fbidq.html?FBgn0032593) | [FBgn0032593](http://flybase.org/cgi-bin/fbidq.html?FBgn0032593) | Transient receptor potential cation channel | [Trpgamma](http://flybase.org/cgi-bin/fbidq.html?FBgn0032593) |
| [CG7573](http://flybase.org/cgi-bin/fbidq.html?FBgn0036153) | [FBgn0036153](http://flybase.org/cgi-bin/fbidq.html?FBgn0036153) | - | [CG7573](http://flybase.org/cgi-bin/fbidq.html?FBgn0036153) |
| [CG7638](http://flybase.org/cgi-bin/fbidq.html?FBgn0036133) | [FBgn0036133](http://flybase.org/cgi-bin/fbidq.html?FBgn0036133) | - | [CG7638](http://flybase.org/cgi-bin/fbidq.html?FBgn0036133) |
| [CG8127](http://flybase.org/cgi-bin/fbidq.html?FBgn0000568) | [FBgn0000568](http://flybase.org/cgi-bin/fbidq.html?FBgn0000568) | Ecdysone-induced protein 75B | [Eip75B](http://flybase.org/cgi-bin/fbidq.html?FBgn0000568) |
| [CG8176](http://flybase.org/cgi-bin/fbidq.html?FBgn0037702) | [FBgn0037702](http://flybase.org/cgi-bin/fbidq.html?FBgn0037702) | - | [CG8176](http://flybase.org/cgi-bin/fbidq.html?FBgn0037702) |
| [CG8195](http://flybase.org/cgi-bin/fbidq.html?FBgn0034032) | [FBgn0034032](http://flybase.org/cgi-bin/fbidq.html?FBgn0034032) | - | [CG8195](http://flybase.org/cgi-bin/fbidq.html?FBgn0034032) |
| [CG8937](http://flybase.org/cgi-bin/fbidq.html?FBgn0001216) | [FBgn0001216](http://flybase.org/cgi-bin/fbidq.html?FBgn0001216) | Heat shock protein cognate 1 | [Hsc70-1](http://flybase.org/cgi-bin/fbidq.html?FBgn0001216) |
| [CG9470](http://flybase.org/cgi-bin/fbidq.html?FBgn0002868) | [FBgn0002868](http://flybase.org/cgi-bin/fbidq.html?FBgn0002868) | Metallothionein A | [MtnA](http://flybase.org/cgi-bin/fbidq.html?FBgn0002868) |
| [CG9745](http://flybase.org/cgi-bin/fbidq.html?FBgn0000412) | [FBgn0000412](http://flybase.org/cgi-bin/fbidq.html?FBgn0000412) | D1 chromosomal protein | [D1](http://flybase.org/cgi-bin/fbidq.html?FBgn0000412) |

**Table S3**

**Genes predominantly expressed in dorsal trunks AND regulated by hypoxia**
