# Supplementary material for: Transcriptional Regionalization of the Fruit Fly’s Airway Epithelium
Source: PLoS One. 2014 Jul 14;9(7):e102534. doi: 10.1371/journal.pone.0102534 (PMC4097054; doi:10.1371/journal.pone.0102534)
Supplement: Table S4 — Genes predominantly expressed in primary/secondary/terminal branches AND regulated by hypoxia. (DOCX) [file pone.0102534.s005.docx]

| **CG** | **Flybase ID** | **name** | **Symbol** |
| --- | --- | --- | --- |
| [CG10305](http://flybase.org/cgi-bin/fbidq.html?FBgn0261597) | [FBgn0261597](http://flybase.org/cgi-bin/fbidq.html?FBgn0261597) | Ribosomal protein S26 | [RpS26](http://flybase.org/cgi-bin/fbidq.html?FBgn0261597) |
| [CG10763](http://flybase.org/cgi-bin/fbidq.html?FBgn0030011) | [FBgn0030011](http://flybase.org/cgi-bin/fbidq.html?FBgn0030011) | Gbeta5 | [Gbeta5](http://flybase.org/cgi-bin/fbidq.html?FBgn0030011) |
| [CG11870](http://flybase.org/cgi-bin/fbidq.html?FBgn0262617) | [FBgn0262617](http://flybase.org/cgi-bin/fbidq.html?FBgn0262617) | - | [CG43143](http://flybase.org/cgi-bin/fbidq.html?FBgn0262617) |
| [CG12279](http://flybase.org/cgi-bin/fbidq.html?FBgn0038080) | [FBgn0038080](http://flybase.org/cgi-bin/fbidq.html?FBgn0038080) | - | [CG12279](http://flybase.org/cgi-bin/fbidq.html?FBgn0038080) |
| [CG12286](http://flybase.org/cgi-bin/fbidq.html?FBgn0001296) | [FBgn0001296](http://flybase.org/cgi-bin/fbidq.html?FBgn0001296) | karmoisin | [kar](http://flybase.org/cgi-bin/fbidq.html?FBgn0001296) |
| [CG13120](http://flybase.org/cgi-bin/fbidq.html?FBgn0265001) | [FBgn0265001](http://flybase.org/cgi-bin/fbidq.html?FBgn0265001) | pickpocket 18 | [ppk18](http://flybase.org/cgi-bin/fbidq.html?FBgn0265001) |
| [CG13131](http://flybase.org/cgi-bin/fbidq.html?FBgn0032175) | [FBgn0032175](http://flybase.org/cgi-bin/fbidq.html?FBgn0032175) | - | [CG13131](http://flybase.org/cgi-bin/fbidq.html?FBgn0032175) |
| [CG13280](http://flybase.org/cgi-bin/fbidq.html?FBgn0032609) | [FBgn0032609](http://flybase.org/cgi-bin/fbidq.html?FBgn0032609) | - | [CG13280](http://flybase.org/cgi-bin/fbidq.html?FBgn0032609) |
| [CG14243](http://flybase.org/cgi-bin/fbidq.html?FBgn0039444) | [FBgn0039444](http://flybase.org/cgi-bin/fbidq.html?FBgn0039444) | TweedleD | [TwdlD](http://flybase.org/cgi-bin/fbidq.html?FBgn0039444) |
| [CG1506](http://flybase.org/cgi-bin/fbidq.html?FBgn0023416) | [FBgn0023416](http://flybase.org/cgi-bin/fbidq.html?FBgn0023416) | Ac3 | [Ac3](http://flybase.org/cgi-bin/fbidq.html?FBgn0023416) |
| [CG15280](http://flybase.org/cgi-bin/fbidq.html?FBgn0028867) | [FBgn0028867](http://flybase.org/cgi-bin/fbidq.html?FBgn0028867) | - | [CR15280](http://flybase.org/cgi-bin/fbidq.html?FBgn0028867) |
| [CG15282](http://flybase.org/cgi-bin/fbidq.html?FBgn0028855) | [FBgn0028855](http://flybase.org/cgi-bin/fbidq.html?FBgn0028855) | - | [CG15282](http://flybase.org/cgi-bin/fbidq.html?FBgn0028855) |
| [CG15286](http://flybase.org/cgi-bin/fbidq.html?FBgn0028531) | [FBgn0028531](http://flybase.org/cgi-bin/fbidq.html?FBgn0028531) | - | [CG15286](http://flybase.org/cgi-bin/fbidq.html?FBgn0028531) |
| [CG15375](http://flybase.org/cgi-bin/fbidq.html?FBgn0029694) | [FBgn0029694](http://flybase.org/cgi-bin/fbidq.html?FBgn0029694) | - | [CG15375](http://flybase.org/cgi-bin/fbidq.html?FBgn0029694) |
| [CG16713](http://flybase.org/cgi-bin/fbidq.html?FBgn0031560) | [FBgn0031560](http://flybase.org/cgi-bin/fbidq.html?FBgn0031560) | - | [CG16713](http://flybase.org/cgi-bin/fbidq.html?FBgn0031560) |
| [CG1722](http://flybase.org/cgi-bin/fbidq.html?FBgn0031168) | [FBgn0031168](http://flybase.org/cgi-bin/fbidq.html?FBgn0031168) | - | [CG1722](http://flybase.org/cgi-bin/fbidq.html?FBgn0031168) |
| [CG1877](http://flybase.org/cgi-bin/fbidq.html?FBgn0015509) | [FBgn0015509](http://flybase.org/cgi-bin/fbidq.html?FBgn0015509) | lin-19-like | [lin19](http://flybase.org/cgi-bin/fbidq.html?FBgn0015509) |
| [CG2053](http://flybase.org/cgi-bin/fbidq.html?FBgn0039887) | [FBgn0039887](http://flybase.org/cgi-bin/fbidq.html?FBgn0039887) | - | [CG2053](http://flybase.org/cgi-bin/fbidq.html?FBgn0039887) |
| [CG2522](http://flybase.org/cgi-bin/fbidq.html?FBgn0010391) | [FBgn0010391](http://flybase.org/cgi-bin/fbidq.html?FBgn0010391) | GTP-binding protein | [Gtp-bp](http://flybase.org/cgi-bin/fbidq.html?FBgn0010391) |
| [CG30035](http://flybase.org/cgi-bin/fbidq.html?FBgn0050035) | [FBgn0050035](http://flybase.org/cgi-bin/fbidq.html?FBgn0050035) | Trehalose transporter 1-1 | [Tret1-1](http://flybase.org/cgi-bin/fbidq.html?FBgn0050035) |
| [CG30121](http://flybase.org/cgi-bin/fbidq.html?FBgn0050121) | [FBgn0050121](http://flybase.org/cgi-bin/fbidq.html?FBgn0050121) | - | [CR30121](http://flybase.org/cgi-bin/fbidq.html?FBgn0050121) |
| [CG3032](http://flybase.org/cgi-bin/fbidq.html?FBgn0029928) | [FBgn0029928](http://flybase.org/cgi-bin/fbidq.html?FBgn0029928) | - | [CG3032](http://flybase.org/cgi-bin/fbidq.html?FBgn0029928) |
| [CG32692](http://flybase.org/cgi-bin/fbidq.html?FBgn0052692) | [FBgn0052692](http://flybase.org/cgi-bin/fbidq.html?FBgn0052692) | - | [CG32692](http://flybase.org/cgi-bin/fbidq.html?FBgn0052692) |
| [CG3655](http://flybase.org/cgi-bin/fbidq.html?FBgn0040397) | [FBgn0040397](http://flybase.org/cgi-bin/fbidq.html?FBgn0040397) | - | [CG3655](http://flybase.org/cgi-bin/fbidq.html?FBgn0040397) |
| [CG4067](http://flybase.org/cgi-bin/fbidq.html?FBgn0020385) | [FBgn0020385](http://flybase.org/cgi-bin/fbidq.html?FBgn0020385) | pugilist | [pug](http://flybase.org/cgi-bin/fbidq.html?FBgn0020385) |
| [CG5163](http://flybase.org/cgi-bin/fbidq.html?FBgn0013347) | [FBgn0013347](http://flybase.org/cgi-bin/fbidq.html?FBgn0013347) | Transcription-factor-IIA-S | [TfIIA-S](http://flybase.org/cgi-bin/fbidq.html?FBgn0013347) |
| [CG5352](http://flybase.org/cgi-bin/fbidq.html?FBgn0262601) | [FBgn0262601](http://flybase.org/cgi-bin/fbidq.html?FBgn0262601) | Small ribonucleoprotein particle protein SmB | [SmB](http://flybase.org/cgi-bin/fbidq.html?FBgn0262601) |
| [CG5397](http://flybase.org/cgi-bin/fbidq.html?FBgn0031327) | [FBgn0031327](http://flybase.org/cgi-bin/fbidq.html?FBgn0031327) | - | [CG5397](http://flybase.org/cgi-bin/fbidq.html?FBgn0031327) |
| [CG5554](http://flybase.org/cgi-bin/fbidq.html?FBgn0034914) | [FBgn0034914](http://flybase.org/cgi-bin/fbidq.html?FBgn0034914) | - | [CG5554](http://flybase.org/cgi-bin/fbidq.html?FBgn0034914) |
| [CG5867](http://flybase.org/cgi-bin/fbidq.html?FBgn0027586) | [FBgn0027586](http://flybase.org/cgi-bin/fbidq.html?FBgn0027586) | - | [CG5867](http://flybase.org/cgi-bin/fbidq.html?FBgn0027586) |
| [CG6742](http://flybase.org/cgi-bin/fbidq.html?FBgn0039056) | [FBgn0039056](http://flybase.org/cgi-bin/fbidq.html?FBgn0039056) | Centaurin beta 1A | [CenB1A](http://flybase.org/cgi-bin/fbidq.html?FBgn0039056) |
| [CG6808](http://flybase.org/cgi-bin/fbidq.html?FBgn0037921) | [FBgn0037921](http://flybase.org/cgi-bin/fbidq.html?FBgn0037921) | - | [CG6808](http://flybase.org/cgi-bin/fbidq.html?FBgn0037921) |
| [CG6933](http://flybase.org/cgi-bin/fbidq.html?FBgn0036952) | [FBgn0036952](http://flybase.org/cgi-bin/fbidq.html?FBgn0036952) | - | [CG6933](http://flybase.org/cgi-bin/fbidq.html?FBgn0036952) |
| [CG7177](http://flybase.org/cgi-bin/fbidq.html?FBgn0037098) | [FBgn0037098](http://flybase.org/cgi-bin/fbidq.html?FBgn0037098) | WNK homolog | [Wnk](http://flybase.org/cgi-bin/fbidq.html?FBgn0037098) |
| [CG8440](http://flybase.org/cgi-bin/fbidq.html?FBgn0015754) | [FBgn0015754](http://flybase.org/cgi-bin/fbidq.html?FBgn0015754) | Lissencephaly-1 | [Lis-1](http://flybase.org/cgi-bin/fbidq.html?FBgn0015754) |
| [CG8556](http://flybase.org/cgi-bin/fbidq.html?FBgn0014011) | [FBgn0014011](http://flybase.org/cgi-bin/fbidq.html?FBgn0014011) | Rac2 | [Rac2](http://flybase.org/cgi-bin/fbidq.html?FBgn0014011) |
| [CG8905](http://flybase.org/cgi-bin/fbidq.html?FBgn0010213) | [FBgn0010213](http://flybase.org/cgi-bin/fbidq.html?FBgn0010213) | Superoxide dismutase 2 (Mn) | [Sod2](http://flybase.org/cgi-bin/fbidq.html?FBgn0010213) |
| [CG8948](http://flybase.org/cgi-bin/fbidq.html?FBgn0030685) | [FBgn0030685](http://flybase.org/cgi-bin/fbidq.html?FBgn0030685) | GTPase regulator associated with focal adhesion kinase ortholog | [Graf](http://flybase.org/cgi-bin/fbidq.html?FBgn0030685) |
| [CG9149](http://flybase.org/cgi-bin/fbidq.html?FBgn0035203) | [FBgn0035203](http://flybase.org/cgi-bin/fbidq.html?FBgn0035203) | - | [CG9149](http://flybase.org/cgi-bin/fbidq.html?FBgn0035203) |
| [CG9453](http://flybase.org/cgi-bin/fbidq.html?FBgn0265137) | [FBgn0265137](http://flybase.org/cgi-bin/fbidq.html?FBgn0265137) | Serpin 42Da | [Spn42Da](http://flybase.org/cgi-bin/fbidq.html?FBgn0265137) |
| [CG9775](http://flybase.org/cgi-bin/fbidq.html?FBgn0037261) | [FBgn0037261](http://flybase.org/cgi-bin/fbidq.html?FBgn0037261) | - | [CG9775](http://flybase.org/cgi-bin/fbidq.html?FBgn0037261) |
| [CG9878](http://flybase.org/cgi-bin/fbidq.html?FBgn0027360) | [FBgn0027360](http://flybase.org/cgi-bin/fbidq.html?FBgn0027360) | Translocase of inner membrane 10 | [Tim10](http://flybase.org/cgi-bin/fbidq.html?FBgn0027360) |

**Table S4**

**Genes predominantly expressed in primary/secondary/terminal branches AND regulated by hypoxia**
