# Supplementary material for: Transcriptional Regionalization of the Fruit Fly’s Airway Epithelium
Source: PLoS One. 2014 Jul 14;9(7):e102534. doi: 10.1371/journal.pone.0102534 (PMC4097054; doi:10.1371/journal.pone.0102534)
Supplement: Table S5 — Oligonucleotides used in this study. (DOCX) [file pone.0102534.s006.docx]

**Table S5**

**Oligonucleotides used in this study**

**Oligonucleotide used to generate promoter Gal4 lines**

***Oa2***-sense: 5`GACGAC CTC CAT TTA ACA CC-3`

***Oa2*-**antisense: 5`TTC CTA GAA CCC AAC ATGCC-3`

***Oamb***-sense: 5`GAG GCG GCC GCT GTA TGA GTG GTG CCA CAGC-3`

***Oamb*-**antisense: 5`GAG AAGATCTAATTGTT CGTT CCGGAACG-3`

***Octβ2R*-**sense: 5`GAG AGG ATC CGG CAT CTT CGG TCC AAT ACC-3`

***Octβ2R***-antisense: 5`GAG AGC GGC CGC GAG TCT CGT TGT CTG GTT-3`

***TyrR***-sense: 5`GAG AGC GGC CGC CTT GCT GAT GCT CTG GTT GG-3`

***TyrR***-antisense : 5`GAG AAG ATC TAA CTT TAG CCA CCC GCA AC -3`

***TyrRII***-sense: 5`GAG AGC GGC CGC CTA ATG CCG GAG ATG AGT GC-3`

***TyRII*-**antisense: 5`GAG AGG ATC CGG TTC ACA ATC GAG TGT GG-3`

**Oligonucleotides for qRT-PCR**

***CG2346***-sense: 5`CATGACGGAGCTGTTCAAGA-3`

***CG2346***-antisense: 5`GACTACCTCACTGGCCGAAG-3`

***CG11720***-sense: 5`TCCATGCACGACATCTAAGC-3`

***CG11720***-antisense: 5`AGTGGTGGTGGCTTTAGTGG-3`

***CG31076***-sense: 5`CCGGTGAGGTATCTCGCTAA-3`

***CG31076***-antisense: 5` CTGGGGCAGTGTTCGTAAAT-3`

***CG1722***-sense: 5`AAGCCGAGACTAGGGCTACC-3`

***CG1722***-antisense: 5`TGCCAATCTTCCCTCAAATC-3`

***CG15699***-sense: 5`GCGCCCTAATCCTACACAGA-3`

***CG15699***-antisense: 5`CTCGGGTCTCAGGTCTCACT-3`

***CG8701***-sense: 5`GAGGACCCTGCCCTAGAATC-3`

***CG8701***-antisense:5`ACGCAGTCGGTCTTACGTCT-3`

***CG10463***-sense: 5`TGGCGACATACTCAGCTACG-3`

***CG10463***-antisense: 5`ACCAGGCCTGTTTCTCCTTT-3`

***CG6105***-sense: 5`TGAACAGGCTCCTCACACAG-3`

***CG6105***-antisense: 5`GATGATGTTGCCCAGTCCTT-3`
